# Ligand-Directed Labeling of the Adenosine A_1_ Receptor in Living Cells

**DOI:** 10.1021/acs.jmedchem.4c00835

**Published:** 2024-07-12

**Authors:** Eleonora Comeo, Joëlle Goulding, Chia-Yang Lin, Marleen Groenen, Jeanette Woolard, Nicholas D. Kindon, Clare R. Harwood, Simon Platt, Stephen J. Briddon, Laura E. Kilpatrick, Peter J. Scammells, Stephen J. Hill, Barrie Kellam

**Affiliations:** † Division of Biomolecular Sciences and Medicinal Chemistry, School of Pharmacy, Biodiscovery Institute, University of Nottingham, Nottingham NG7 2RD, U.K.; ‡ Division of Physiology, Pharmacology and Neuroscience, School of Life Sciences, Queens Medical Centre, University of Nottingham, Nottingham NG7 2UH, U.K.; § Centre of Membrane Proteins and Receptors (COMPARE), University of Birmingham and University of Nottingham, The Midlands NG7 2UH, U.K.; ∥ Medicinal Chemistry, Monash Institute of Pharmaceutical Sciences, Monash University, Parkville, Victoria 3052, Australia

## Abstract

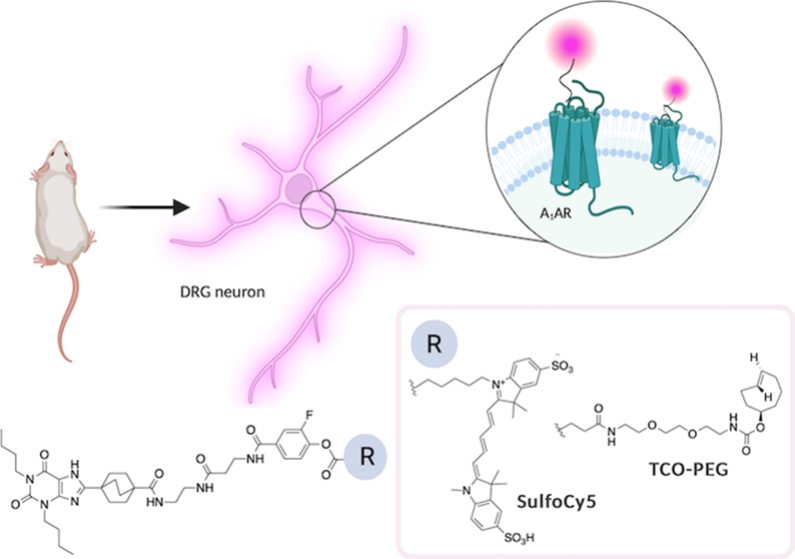

The study of protein function and dynamics in their native cellular environment is essential for progressing fundamental science. To overcome the requirement of genetic modification of the protein or the limitations of dissociable fluorescent ligands, ligand-directed (LD) chemistry has most recently emerged as a complementary, bioorthogonal approach for labeling native proteins. Here, we describe the rational design, development, and application of the first ligand-directed chemistry approach for labeling the A_1_AR in living cells. We pharmacologically demonstrate covalent labeling of A_1_AR expressed in living cells while the orthosteric binding site remains available. The probes were imaged using confocal microscopy and fluorescence correlation spectroscopy to study A_1_AR localization and dynamics in living cells. Additionally, the probes allowed visualization of the specific localization of A_1_ARs endogenously expressed in dorsal root ganglion (DRG) neurons. LD probes developed here hold promise for illuminating ligand-binding, receptor signaling, and trafficking of the A_1_AR in more physiologically relevant environments.

## Introduction

**Figure 1:**
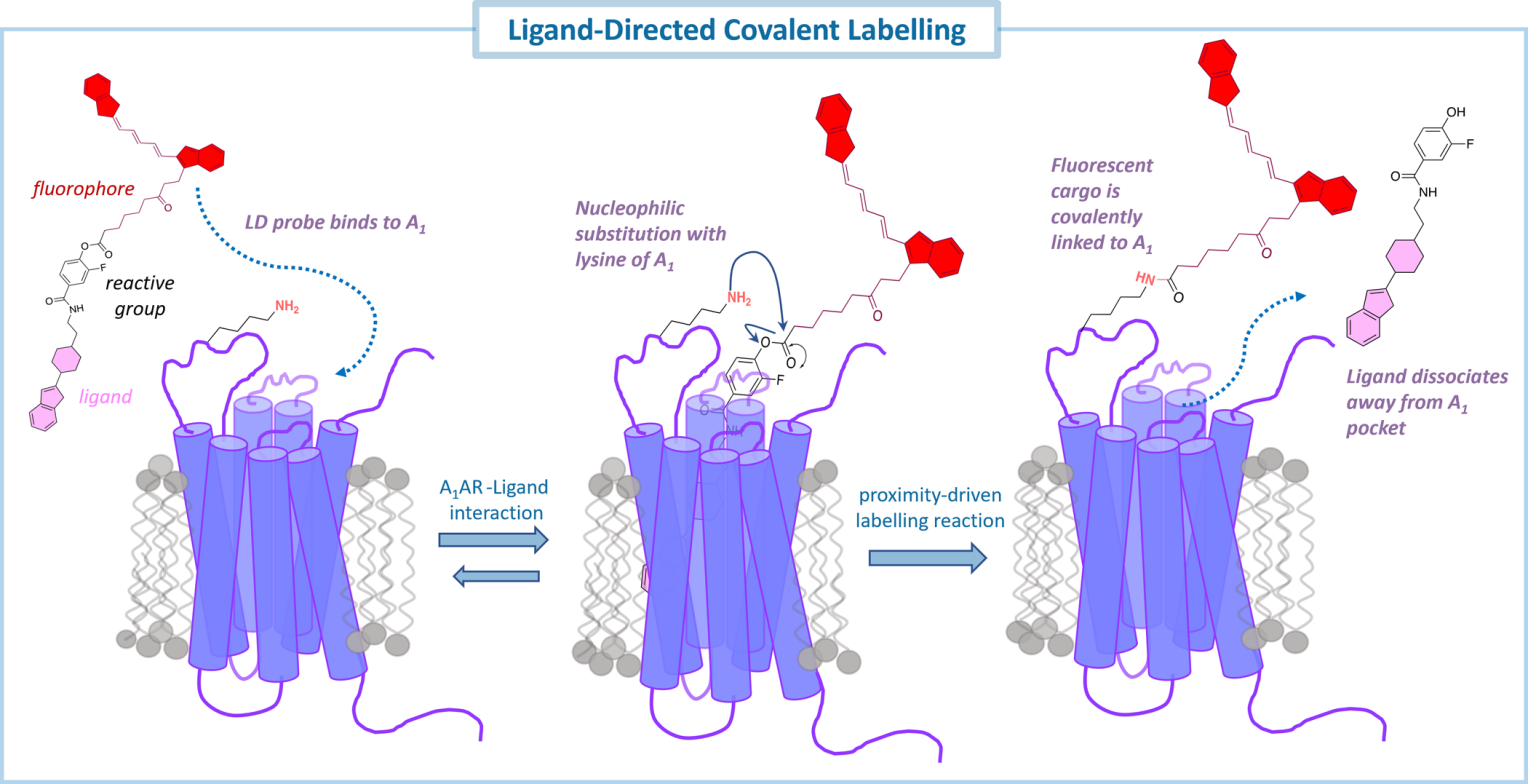
Schematic representation of ligand-directed labeling of a GPCR. Upon ligand binding, the electrophilic carbonyl group undergoes nucleophilic attack by a nucleophilic residue (here represented as lysine) side chain in proximity to the binding site; the functional probe (here represented by a red-shifted fluorophore) is covalently attached to the receptor, and the affinity guide ligand freely dissociates (dashed line) leaving the binding site available for additional ligands to bind to the target protein receptor.

The study of protein function and dynamics in native cellular environments is essential for progressing fundamental science, which can potentially benefit through the advancement of more selective and efficient therapeutic interventions. Over the past 2 decades, scientists have made enormous progress in tackling the challenges associated with the complexity of cellular systems, by developing new technologies to allow the study of proteins in their native cellular settings, such as in live cells or in vivo.^1,2^ In the context of G protein-coupled receptor (GPCR) research for example, the genetic fusion of a fluorescent protein tag such as green fluorescent protein (GFP) or self-labeling tag proteins such as SNAP-tag^3^ and Halo-tag^4^ have revolutionized the ways by which GPCR localization, dynamics, and functions can be monitored.^5^ Although powerful, these techniques possess some limitations: the tag size and the need to genetically engineer the protein to express the tag moiety may disturb or alter the native functions of the protein, thereby introducing artifacts that can cause misinterpretation of biological data. Moreover, these methods cannot be readily applied to studies in endogenous expressing systems.^5,6^ Fluorescent ligands offer an alternative and powerful avenue to investigate the real-time function of receptors in their native cellular environments.^7^ Yet, the inherent, reversible nature of ligand binding may still hamper the extent to which many temporal and spatial aspects of GPCR pharmacology can be investigated.^8^ In this regard, ligand-directed (LD) chemistry^9,10^ has most recently emerged as a complementary, bioorthogonal approach for labeling native proteins, whereby an affinity “guide” ligand for the protein of interest is conjugated to a reporter functional group via an electrophilic reactive linker; upon ligand binding, the close proximity of this electrophilic region with a nucleophilic amino acid side chain (e.g., lysine) in the binding site effectively increases the rate of the substitution reaction with the reactive group, resulting in covalent transfer of the chemical cargo to the protein. The guide ligand therefore acts as a leaving group, which can then freely dissociate and leave the binding site of the protein intact (Figure 1).

The adenosine A_1_AR receptor is a subtype of the four adenosine receptors, a class of GPCRs that are activated by the endogenous purine nucleoside adenosine.^11,12^ Modulation of the A_1_AR pharmacology provides therapeutic opportunities for chronic and acute disease conditions, including cardiovascular and respiratory diseases, central nervous system (CNS) disorders, and inflammatory diseases such as cancer and neuropathic pain.^13^ Recent studies have shown that targeting A_1_AR signaling through allosteric modulators^14^ and biased agonists^15^ could represent a promising therapeutic strategy for treating chronic pain with nonopioid analgesic agents.^16^ However, despite their significant potential, the clinical translation of A_1_AR-targeting small molecules has suffered from a limited understanding of the function of A_1_AR and the mechanisms and differences that regulate the spatial (e.g., specific cellular localization and environment) and temporal (e.g., signaling duration) dynamics of its biology in living cells.^13,17^ To address these problems, the development of a ligand-directed labeling approach for the permanent bioconjugation of the A_1_AR with a functional probe could provide a noninvasive approach for monitoring and studying receptor function and dynamics in its native health and disease conditions without genetic manipulation. Indeed, a similar approach was leveraged to enable the covalent bioconjugation of another adenosine receptor subtype, namely, the A_2A_AR, with a SulfoCy5 fluorophore in living cells.^18^ The ligand-directed compound could selectively label endogenous A_2A_AR, thereby enabling their detection in human monocyte-derived macrophages by FACS and visualization on a human breast cancer cell line using confocal microscopy.

The utility of ligand-directed technology for labeling membrane receptors in living cells has also been described for a small number of other GPCRs: the bradykinin B2 receptor^19^ with biotin and the μ opioid^20^ and dopamine D1^21^ and cannabinoid CB_2_^22^ receptors with a fluorescent group.

In the present study, we describe the design and application of a ligand-directed chemistry technology that allows selective labeling of endogenous A_1_ARs with functional probes and monitors its localization and dynamics in living cells.

## Results

### Rational Design of Ligand-Directed Probes

**Figure 2:**
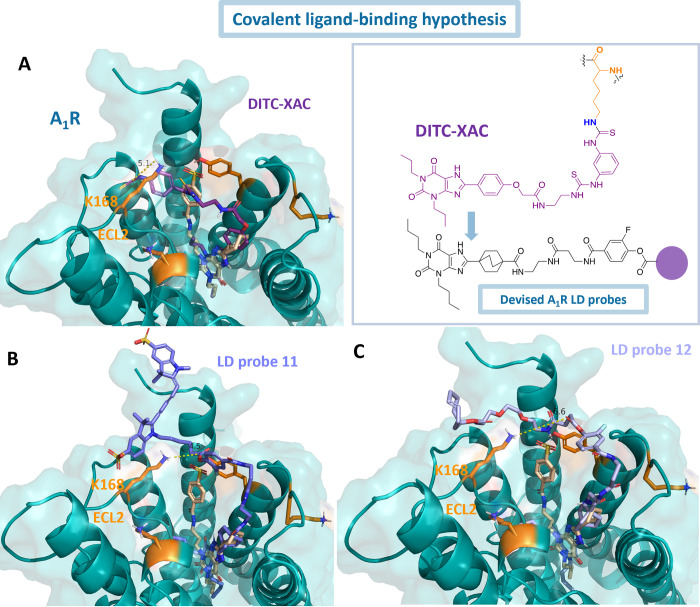
Molecular modeling of DITC-XAC (A), ligand-directed (LD) probe 11 (B) and LD probe 12 (C) to the crystal structure of the hA_1_AR (PDB: 5UEN) performed with Schrödinger’s Glide (Schrödinger release 2022-2). Figures show a focused view of the orthosteric binding site of the hA_1_AR with cocrystallized DU172. DITC-XAC is illustrated in violet-purple sticks, LD probe **11** is rendered in steel blue sticks, while LD probe **12** is depicted in light blue sticks. The hA_1_AR is shown in ribbon with the surface set at 80% transparency, both colored in aquamarine. Hydrogen bonds are shown as yellow-dashed lines and the extracellular loop (ECL) 2 and lysine 168 (K168) are labeled for clarity. Images were generated using PyMOL (2.5.4). The panel (framed within a light blue solid line) on the right of panel A depicts a schematic representation of the covalent ligand-binding hypothesis of DITC-XAC, which inspired the design of our ligand-directed probes. The devised LD A_1_AR probe is shown with a general structure whereby the phenoxy acyl group, depicted here as a purple-colored circle, may represent a different array of functional cargo (e.g., fluorophore, clickable ligand) depending on the final application of the LD compound.

To develop ligand-directed (LD) probes capable of labeling the A_1_AR in living cells, we designed labeling reagents comprising an A_1_AR-recognition element and a functional cargo connected via a reactive cleavable linker. We applied a structure-based design approach, whereby available A_1_AR structural data were leveraged to guide close proximity between the reactive cleavable linker and a nucleophilic residue distal to the binding site of the A_1_AR (Figure 2).

The design of A_1_AR LD probes was inspired by combining features of reported reversible subtype-selective fluorescent A_1_AR antagonists^23^ and an irreversible A_1_AR antagonist, *p*-DITC-XAC.^24^ Moreover, the A_1_AR-recognition element of the targeted A_1_LD probes features a xanthine-based scaffold substituted with *N*^1^- and *N*^3^-butyl alkyl chains and a C8 bicyclo[2.2.2]octane group as these structural features proved advantageous for achieving higher A_1_AR ligand-binding affinity and selectivity.^23,25,26^*p*-DITC-XAC^24,27^ is an irreversible analogue of xanthine amine congener (XAC)^28^ integrating an electrophilic isothiocyanate group at the *para* position of the aromatic ring distal to the xanthine core. When this molecule was first described and characterized, neither structural information nor sequence analysis was available for the targeted A_1_AR.^11^ With the recent availability of A_1_AR structural data,^29−32^ we performed molecular docking simulations to investigate possible covalent ligand-A_1_AR binding interactions (Figure 2A) established between *p*-DITC-XAC and A_1_AR (PDB: 5UEN, cocrystal with DU172)^30^ and to explore close proximity between the electrophile isothiocyanate group and nucleophilic residues distal to the binding site of the A_1_AR, which could enable probe anchoring. From in silico studies, *p*-DITC-XAC was predicted to engage with A_1_AR in a mode comparable to DU172 and reported cocrystallized xanthine-based analogues, whereby the xanthine motif is embedded within the binding pocket of A_1_AR and the substituents at the 8-position of the xanthine scaffold oriented toward the extracellular face of the receptor.^30,31,33^ Moreover, the *p*-isothiocyanate-phenyl group was predicted to occupy the solvent-exposed region of the A_1_AR, with the electrophilic moiety being in close proximity to the ε-amino group of residue K168^ECL2^. We reasoned that the small phenyl ester group substituted at the 3-position with a fluorine atom, the most electronegative element, would be a suitable reactive functionality to allow the bioconjugation of a functional probe to A_1_AR as this activating group was successfully applied for ligand-directed labeling of the A_2A_AR in living cells.^18^ Moreover, the fluoro-substituted phenyl ester reactive group complements previously reported ligand-directed chemistries pioneered by Hamachi’s group, such as acyl imidazole (LDAI),^10^ bromo benzoate (LDBB),^34^ and *N*-acyl-*N*-alkyl sulfonamide (LDNASA)^35^ labeling approaches. However, the fluoro-substituted phenyl ester reactive group displayed additional advantages, including smaller size, with the possibility of tuning the reactivity of the ester by the removal or introduction of additional electron-withdrawing fluorine atoms into the phenyl ring. In addition, many fluorinated phenolic benzoic acid starting materials are readily available and they can be easily, synthetically incorporated into specific affinity ligands for a protein under investigation. Accordingly, the small phenyl ester warhead was introduced as a chemo-reactive group between the orthosteric moiety (guide ligand) and the functional probe of the targeted A_1_AR ligand-directed labels, with the phenyl ester constituting part of the orthosteric binding moiety. Moreover, as we aimed to develop LD probes that could be applied to study the molecular pharmacology of the A_1_AR across multiple experimental settings, we tethered the probes with two different functional cargoes, the water-soluble sulfonated cyanine5 (SulfoCy5) dye and the reactive, strained alkene *trans*-cyclooctene (TCO) group, respectively. SulfoCy5 is a far-red emitting fluorophore that displays advantageous photochemical properties for advanced imaging studies, including high absorption coefficient (ε) and molecular brightness (ε × ϕ, a parameter defining the efficiencies of the amount of light absorbed and fluorescence)^36^ in addition to emitting in the region of the spectrum (650–670 nm) where the degree of background autofluorescence from living cells is minimized.^37^ Moreover, owing to its favorable water solubility, the application of the SulfoCy5 fluorophore leads to a decreased degree of nonspecific membrane accumulation compared to more lipophilic dyes. The *trans*-cyclooctene (TCO) group encompasses a particularly versatile functional group for ligand-directed chemistries as this reactive olefin (electron-rich dienophile) undergoes ultrafast inverse electron demand Diels–Alder (IEDDA)^38,39^ click reaction with tetrazines (electron-poor diene) to produce a dihydropyridazine cycloadduct upon the loss of N_2_.^40^ In principle, this methodology offers the opportunity to use a single ligand-directed probe bearing a reactive handle that can be used for labeling the protein under study with a different range of organic dyes or probes as required.^6,40^ Furthermore, tetrazine-conjugated fluorophores are fluorogenic, meaning that the fluorescence intensity of the conjugated fluorophore increases upon reaction with the corresponding dienophile (e.g., TCO) via the IEDDA reaction. This “turn-on” system is particularly advantageous for imaging studies in living cells owing to the higher signal-to-noise (S/N) ratio.^41^

**Scheme 1:**
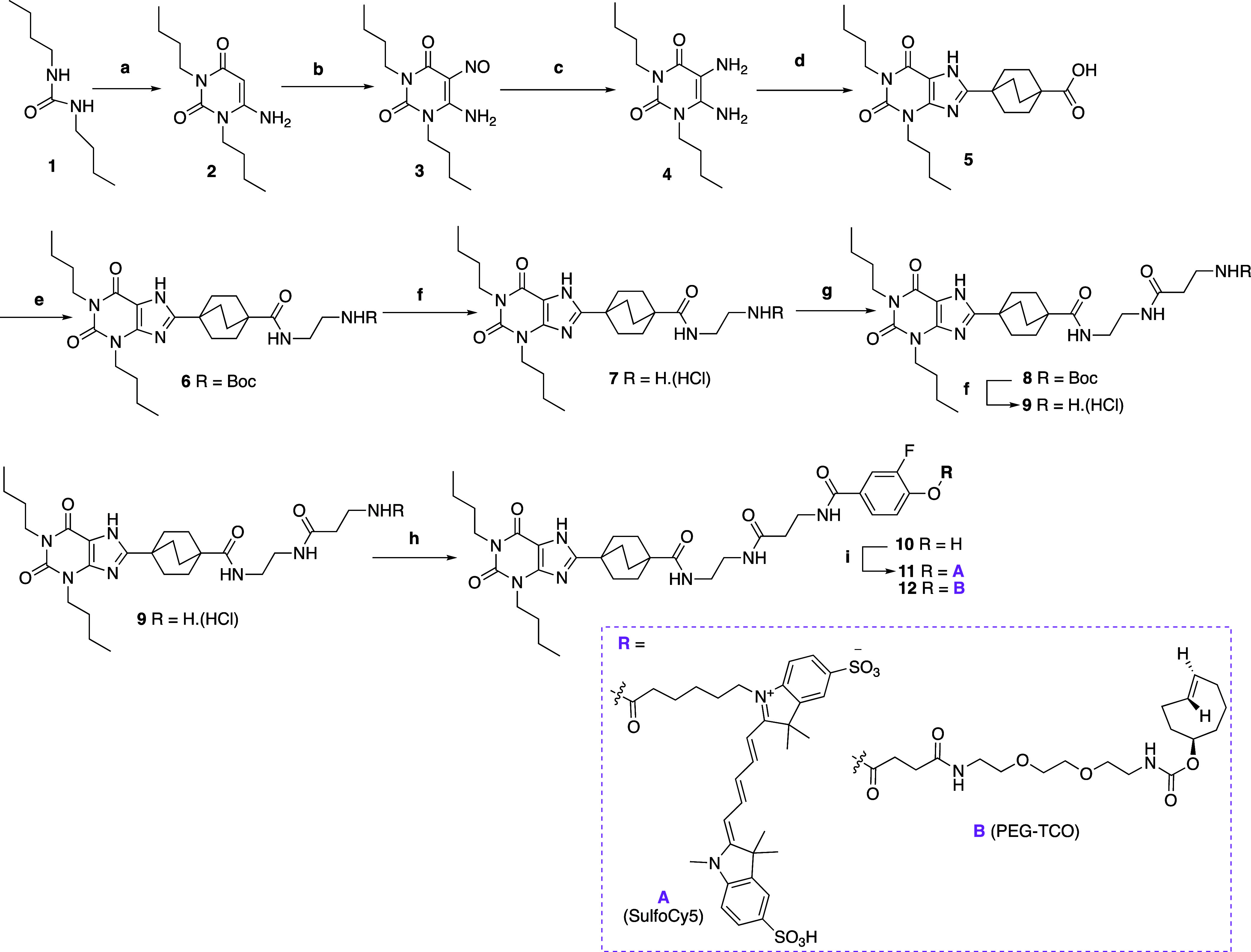
Synthesis of 3-Fluorophenyl Ester-Linked Ligand-Directed Probes Reagents and conditions: (a) (i) cyanoacetic acid, Ac_2_O, 83 °C, 2 h, (ii) H_2_O, 70% NaOH, room temperature (rt), 64%; (b) NaNO_2_, 50% AcOH, 57 °C, 1 h, 41%; (c) Na_2_S_2_O_4_, 12.5% NH_4_OH, 60 °C, 30 min, 80%; (d) (i) 4-(methoxycarbonyl)bicyclo[2.2.2]octane-1-carboxylic acid, *N*,*N*-diisopropylethylamine (DIPEA), COMU, dimethylformamide (DMF), rt, 15 min (ii) 1.0 M KOH, propan-2-ol, reflux, 2 h, 68% (over two steps); (e) *N*-Boc ethylenediamine, COMU, DIPEA, DMF, rt 53%; (f) 4 M HCl in dioxane, rt, 1 h, quantitative; (g) Boc-β-Ala-OH, COMU, DIPEA, DMF, rt, 88%; (h) 3-fluoro-4-hydroxybenzoic acid, COMU, DIPEA, DMF, 1 h, 90 °C, 53%; (i) respective probe (−CO_2_H), 2-bromo-1-ethyl-pyridinium tetrafluoroborate (BEP), DIPEA, DMF, 15 min, rt, then amine, overnight 29–75%.

To understand their covalent ligand–receptor interactions, the designed LD probes **11** and **12** (Scheme 1) were also modeled to the crystal structure of the A_1_AR (Figure 2B,C). Molecular docking studies revealed a close proximity of the electrophilic 3-fluorophenolic ester and nucleophilic residues on the surface of A_1_AR, particularly to K168^ECL2^, where the approximate molecular distances from the carbonyl of the phenyl ester and the nucleophilic ε-amino group of K168^ECL2^ were found to be consistently 4–6 Å.

### Chemistry

The ligand-directed probes were designed and synthesized based on the 8-bicyclo[2.2.2]octylxanthine scaffold, the synthetic route of which is illustrated in Scheme 1. First, we embarked on the synthesis of the core xanthine scaffold following established procedures.^42−44^ This involved reacting *N*-butylamine with butyl isocyanate to yield the corresponding 1,3-dibutylurea (**1**). This was readily reacted with cyanoacetic acid in the presence of acetic anhydride at 83 °C to afford intermediate 2-cyano-*N*-butyl-*N*-(butylcarbamoyl) acetamide, which was cyclized to the corresponding 1,3-dibutyl-6-amino uracil (**2**)^45^ upon the addition of few drops of 70% NaOH. A nitroso group was introduced via electrophilic substitution at the 5-position by means of sodium nitrite in aqueous acetic acid to afford the corresponding 6-amino-5-nitroso-1,3-dibutylpyrimidine-2,4(1*H*,3*H*)-dione (**3**) as a pink solid.^43,46^ Reduction of **3** with sodium dithionite in aqueous ammonia yielded the desired 5,6-diamino-1,3-dibuylpyrimidine-2,4(1*H*,3*H*)-dione (**4**).^42,43^ This was immediately coupled with commercially available 4-(methoxycarbonyl) bicyclo[2.2.2]octane-1-carboxylic acid, employing COMU as the coupling reagent in the presence of DIPEA at rt. Removal of water-soluble products by means of sequential acidic and basic workups, with 10% citric acid and sat. NaHCO_3_, respectively, yielded the crude 6-amino-5-carboxamidouracil intermediate; subsequent dehydrative ring-closure to the corresponding 8-bicyclo[2.2.2]octylxanthine derivative (**5**) was achieved in a solution of 1 M KOH and propran-2-ol under reflux for 2 h. Carboxylic acid **5** was coupled with commercially available *N*-Boc ethylenediamine to afford intermediate **6**. Intermediate **6** was subjected to acidolytic *N*-Boc-removal to afford the corresponding amine **7** as its HCl salt. This was coupled to Boc-β-Ala-OH in the presence of COMU to obtain carboxamide intermediate **8**. Following *N*-Boc deprotection, amine **9** was reacted with 3-fluoro-4-hydroxybenzoic acid to yield xanthine-based phenol **10**. Activation of the given functional probe, namely, SulfoCy5 (**A**) and PEG-TCO (**B**), respectively, in the presence of BEP and DIPEA, followed by reaction with phenol **10**, furnished the title 3-fluorophenyl benzoate conjugates **11** and **12**. The novel synthesized ligand-directed labels were purified by reversed phase high-performance liquid chromatography (RP-HPLC) and the high purity of each final probe was assessed and confirmed by analytical RP-HPLC with a photodiode array detector and was confirmed as >98% homogeneity. Furthermore, the chemical identity of the final compounds was confirmed by NMR and high-resolution mass spectroscopy (HRMS).

### Pharmacology

The ligand-binding properties of the newly synthesized probes were assessed in a series of pharmacological assays. Initially, a NanoBRET^47,48^ saturation binding experiment was used to evaluate the binding affinity of ligand-directed probes functionally labeled with the SulfoCy5 fluorophore (**11**) to the NanoLuc(NL)-tagged^49^ hA_1_AR expressed in HEK293T cells. NanoBRET competition ligand-binding experiments were performed with **12**, which was functionalized with the clickable group *trans*-cyclooctene (TCO) coupled with a polyethylene (PEG) linker with the known A_1_AR selective antagonist DPCPX used as the control.

**Figure 3:**
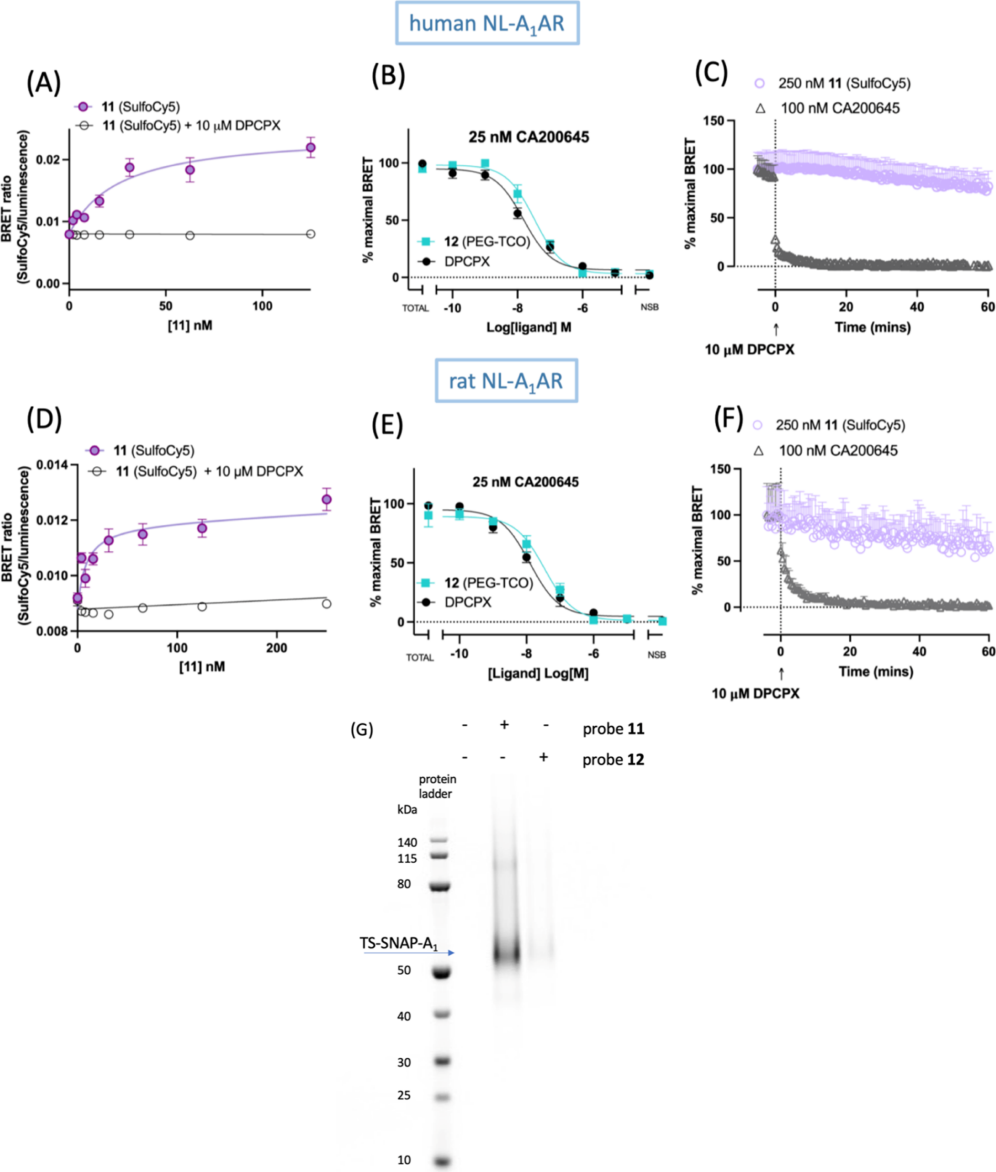
Molecular pharmacology and biochemical characterization of **11** and **12**. NanoBRET ligand-binding data were measured in HEK293 cells stably expressing the human NL-A_1_AR (A–C) or rat NL-A_1_AR (D–F). (A, D) NanoBRET saturation ligand-binding curves obtained by treating the cells with increasing concentration of **11** (0–125 nM (A), human NL-A_1_AR) and (0–250 nM (D), rat NL-A_1_AR) for 1 h at 37 °C in the absence (closed purple circles) and presence (open circles) of 10 μM A_1_AR selective competitive antagonist DPCPX, where the latter was used to determine nonspecific binding. (B, E) Inhibition of CA200645 specific binding (25 nM) in the presence of increasing concentrations of competitive ligands (**12** and DPCPX). Data were normalized to maximal BRET signals in the absence of unlabeled competing ligands (total binding, TB). (C, F) NanoBRET dissociation kinetics experiments with **11** (SulfoCy5) performed in human and rat NL-A_1_AR HEK293T cells, respectively. Cells were treated with 250 nM **11** (SulfoCy5, lilac open circles) for 5 h and CA200645 (black open triangles) for 2 h. After baseline BRET was read every 30 s for 5 min, 10 μM DPCPX was added and BRET measurements were taken every 30 s over the subsequent 1 h. Specific BRET was calculated after subtraction of nonspecific binding component, determined in the presence of 10 μM DPCPX, from the total binding. Each data point represents the combined mean ± SEM from *n* = 5 (A, C), *n* = 4 (B, D), and *n* = 3 (E, F) experiments, each one performed in triplicate. (G) HEK293G cells stably expressing TS-SNAP-A_1_AR were treated with 300 nM **11** or 130 nM **12** in serum-free media for 2 h at 37 °C and 5% CO_2_. For samples treated with compound **12**, the labeling solution was removed and immediately replaced with 10 mL of serum-free media containing 1 μM **Met-Tet-Cy5** and incubated at 37 °C and 5% CO_2_ for a further 15 min. Untreated cells were used as a control. TS-SNAP-A_1_AR was purified and separated on an SDS-PAGEgel gel, and direct Cy5 fluorescence was visualized using in-gel fluorescence. The gel shown is representative of three independent experiments.

A concentration-dependent increase in bioluminescence resonance energy transfer (BRET) signal was observed with the fluorescently labeled ligand **11** (SulfoCy5) (Figure 3). This could be inhibited by cotreatment of the cells with 10 μM DPCPX, indicating that the binding was specific to NL-hA_1_AR. From these curves, it was therefore possible to measure apparent equilibrium dissociation constants (*K*_D_), subsequently converted to p*K*_D_= 7.51 ± 0.12, *n* = 5 for **11** (SulfoCy5). In parallel, nonfluorescent phenyl ester **12** (PEG-TCO) produced a concentration-dependent inhibition of the binding of the reversible nonselective adenosine receptor fluorescent antagonist CA200645^55^ (25 nM) to the NL-hA_1_AR, with a p*K*_i_ value of 7.87 ± 0.16, *n* = 4 (Figure 3 and Table 1).

**Table 1:** Apparent Binding Affinities of the 8-Bicyclo[2.2.2]octylxanthine-Based Phenyl Esters **11** (SulfoCy5) and **12** (TCO-PEG)

		affinity values (log *M*) ± SEM^a^				
cmpd	probe	NL-hA_1_AR	NL-rA_1_AR	NL-hA_2A_AR	NL-hA_2B_AR	NL-hA_3_AR
**11**^a^	SulfoCy5	7.51 ± 0.12 (5)^d^	8.04 ± 0.13 (4)	6.61 ± 0.26 (4)	<6 (4)	<6 (3)
**12**^b^	PEG-TCO	7.87 ± 0.16 (4)	7.99 ± 0.15 (3)	5.86 ± 0.03 (4)	6.09 ± 0.06 (4)	<5 (3)
DPCPX^b^,^c^		8.21 ± 0.09 (4)	8.38 ± 0.17 (3)			

a p*K*_D_ values were calculated as the negative logarithm of the equilibrium dissociation constant (*K*_D_ in nM) measured by NanoBRET saturation ligand-binding assay.

b p*K*_i_ value calculated from the negative logarithm of the equilibrium inhibitory constant (*K*_i_ in nM) measured by NanoBRET competition ligand-binding assay using CA200645 (25 nM) as fluorescent tracer in HEK293T cells stably expressing the human or rat NanoLuc-A_1_AR, the human NanoLuc-A_2B_AR, NanoLuc-A_3_AR or transiently expressing the human NanoLuc-A_2A_AR, respectively. Ligand-binding measurements of subtype-selective ligands used as controls for binding studies at each subtype of adenosine receptor: ZM241385 p*K*_i_(NL-hA_2A_AR) = 8.20 ± 0.08 (4), PSB603 p*K*_i_(NL-hA_2B_AR) = 8.30 ± 0.16 (4) and MRS1220 p*K*_i_(NL-hA_3_AR) = 8.57 ± 0.18 (3), values similar to those quantified in previous studies.^48,50−53^

c p*K*_i_ values for DPCPX comparable to values measured by Cooper^54^ et al., and Stoddart et al.^48^

d p*K*_i_ values obtained significantly differ between human NL-A_1_AR and rat NL-A_1_AR species (*P < 0.05; unpaired *t* test). Values represent the mean ± standard error of the mean (SEM) from *n* separate experiments (value in parentheses) performed in triplicate.

To assess the ability of **11** (SulfoCy5) to covalently transfer the fluorophore to the hA_1_AR, NanoBRET dissociation experiments were performed, whereby an excess (10 μM) of A_1_AR-selective antagonist DPCPX was added to NL-hA_1_AR HEK293T cells previously labeled with 250 nM **11** for 5 h. BRET was monitored over the subsequent 60 min, and very little change in the BRET signal was observed following the addition of DPCPX. The small reduction in the BRET signal observed toward the end of the reads could be mostly likely ascribed to substrate (furimazine) consumption. These results indicate that the fluorophore (SulfoCy5) remained in close proximity to the NL-hA_1_AR and could not be displaced by DPCPX, suggesting a covalent transfer of the fluorophore to the receptor. In contrast, when the reversible fluorescent antagonist CA200645 was subjected to the same experimental conditions, following the addition of 10 μM DPCPX, the measured BRET signal returned to baseline within 4.7 min (Figure 3C). A comparable fast 1/*k*_off_ for CA200645 was also measured in SNAP-A_2_AR HEK293Glosensor cells.^18^ To confirm covalent labeling of A_1_AR by **11** and **12**, HEK293G cells stably expressing a Twin-Strep-SNAP-A_1_AR(TS-SNAP-A_1_R) construct were treated with **11** or **12** prior to purification, separation by sodium dodecyl sulfate-polyacrylamide gel electrophoresis (SDS-PAGE) and visualization of labeled samples by in-gel fluorescence (Figure 3G). In the case of **12**, 2 h after incubation with cells, the click chemistry reagent Met-Tet-SulfoCy5 (1 μM) was added to label the receptor with SulfoCy5 prior to separation by SDS-PAGE. For both **11** and **12**, a strong band corresponding to SulfoCy5-labeled TS-SNAP-A_1_AR was observed at ca. 59.1 kDa, the expected molecular weight of a monomeric SNAP-A_1_AR.^24,56^

As the first step toward the preclinical validation of drug candidates consists of their evaluation in animal models and interspecies differences of a compound’s potency and selectivity have been reported,^54,57,58^ we also set out to quantify the ligand-binding properties of **11** (SulfoCy5) and **12** (PEG-TCO) at rat NL-A_1_AR (Figure 3D–F; Table 1). LD probe **12** showed similar affinities between human and rat species (Table 1), while LD probe **11** exhibited significantly higher affinity for the rat NL-A_1_AR compared to the human species (Table 1). Importantly, the NanoBRET dissociation experiment performed in rat NL-A_1_AR HEK293 cells prelabeled with 250 nM **11** suggested that the ability to covalently transfer the fluorophore to the A_1_AR by LD probe **11** could be retained across species (Figure 3F).

**Figure 4:**
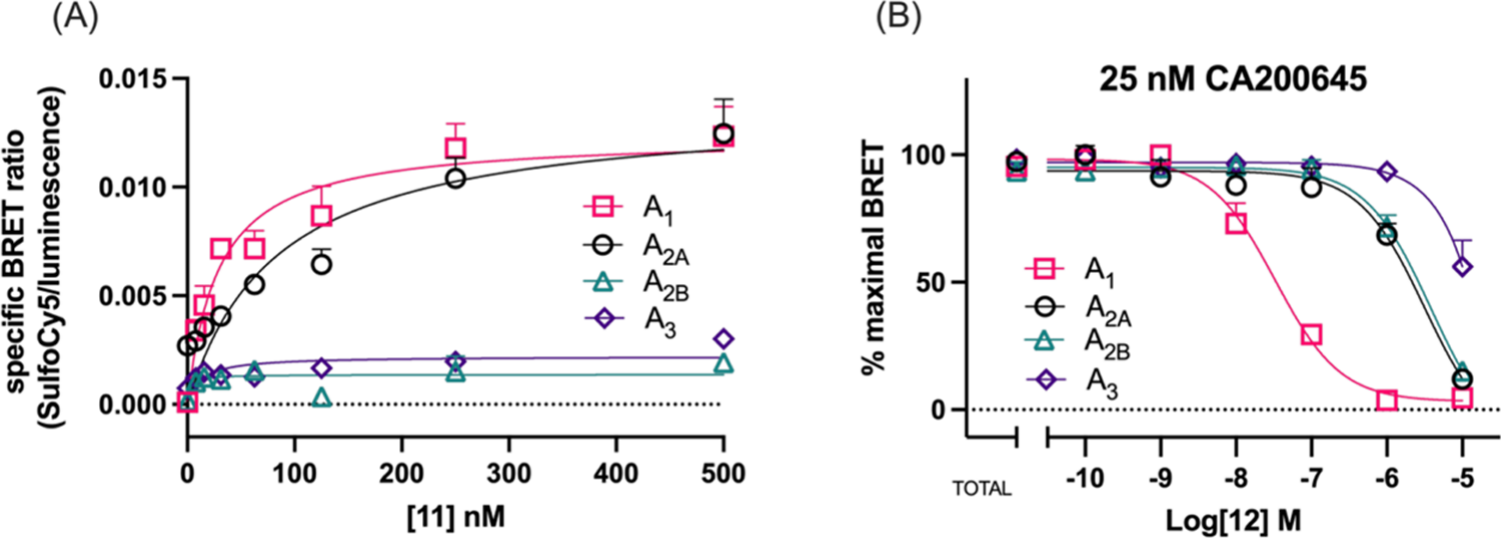
NanoBRET ligand-binding curves obtained by treating HEK293 cells expressing each adenosine receptor subtype with **11** (A) or **12** (B). (A) Specific binding of **11** was measured from saturation binding curves after subtraction of the nonspecific binding component from the total binding. Nonspecific binding was determined in the presence of 10 μM ZM241385 for NL-hA_2A_AR, 10 μM PSB603 for NL-hA_2B_AR, or 10 μM MRS1220 for NL-hA_3_AR. (B) Inhibition of CA200645 specific binding to each subtype of adenosine receptor in the presence of increasing concentrations of **12**. Data were normalized to the maximal BRET signal in the absence of unlabeled competing ligands (total binding, TB). Data points represent the combined mean ± SEM from *n* = 4 (NL-hA_2A_AR and NL-hA_2B_AR) and *n* = 3 (NL-hA_3_AR) experiments performed in triplicate.

Probes **11** and **12** were further evaluated for their ability to bind the other three adenosine receptor subtypes (NL-A_2A_AR, NL-A_2B_AR, and NL-A_3_AR), and their ligand-binding properties were quantified in the NanoBRET saturation and inhibition binding assays, respectively (Table 1 and Figure 4). Specifically, **11** (SulfoCy5) exhibited 7-fold greater affinity for the NL-A_1_AR compared to the NL-A_2A_AR, with minimal BRET signal detected at either NL-A_2B_AR and NL-A_3_AR at concentrations tested (0–500 nM) (Table 1 and Figure 4A). Probe **12** (PEG-TCO) showed 104-fold, 61-fold and 857-fold greater affinity for NL-A_1_AR for the NL-A_2A_AR, NL-A_2B_AR, and NL-A_3_AR, respectively (Table 1 and Figure 4B).

**Figure 5:**
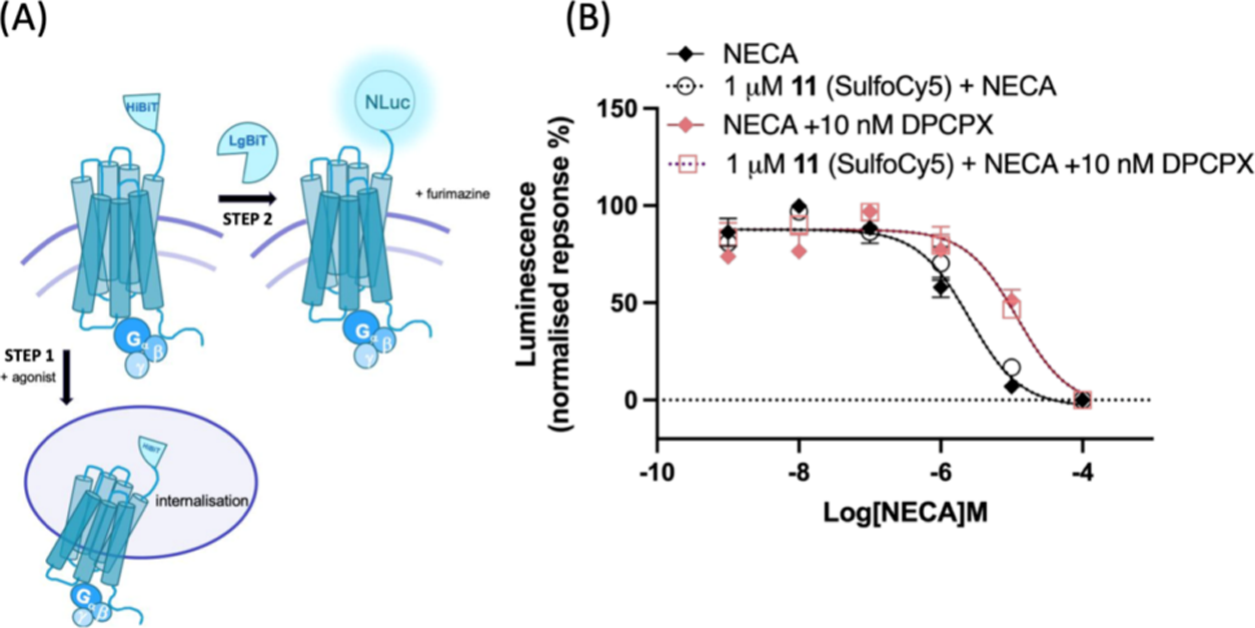
NanoBiT (NanoLuc Binary Technology) complementation assay was used to monitor agonist-induced internalization of HiBiT-tagged A_1_AR HEK293T living cells. (A) Schematic representation of the NanoBiT internalization assay. Step 1: The first step of the assay involves the addition of the agonist (NECA) to stimulate an internalization response of HiBiT-tagged A_1_AR expressed at the cell membrane, whereby HiBiT-A_1_AR is subsequently removed from the cell membrane. Step 2: Treatment of the cells with exogenous purified LgBiT (10 nM), which is cell impermeable, reconstitutes the full-length NanoLuc luciferase, following complementation with HiBiT-tagged receptors localized at the cell surface. The addition of the substrate furimazine leads to a luminescence signal, which can be quantified. The higher the amount of receptor internalized, the lower the intensity of the signal is as a result of reduced availability of receptors on the membrane. (B) Effect of A_1_AR-selective antagonist DPCPX on the NECA-mediated internalization response in HiBiT-A_1_AR HEK293T living cells. Internalization of HiBiT-A_1_AR following agonist (NECA) treatment in the presence and absence of 10 nM competitive antagonist DPCPX. Cells were treated with (open symbols) or without (closed symbols) 1 μM **11** (SulfoCy5) for 1 h. Cells were washed twice prior to the addition of the competitive antagonist DPCPX, which was incubated for 10 min, followed by the addition of increasing concentrations of NECA for 2 h. The decay of the luminescence signal as a result of the loss of the receptor from the cell surface was quantified. Data are normalized to basal (in the absence of NECA treatment). Data points represent the combined mean ± SEM from *n* = 4 separate experiments performed in triplicate.

In principle, ligand-directed probes **11** and **12** were designed to label the A_1_AR with a functional cargo (SulfoCy5 and PEG-TCO, respectively), whereby, following the labeling reaction, the resulting A_1_AR-recognition portion of the probe could freely dissociate from the ligand-binding site of the receptor, allowing A_1_-engagement by additional ligands. Nevertheless, it is possible that the covalently bound functional cargo may block the receptor binding site and hinder the subsequent binding by additional molecules.^59^ Therefore, it was important to confirm that the A_1_AR maintained its functionality when labeled. The A_1_AR primarily couples to the G_i/o_ family of heterotrimeric G proteins, resulting in adenylyl cyclase inhibition and subsequent reduction of cyclic AMP production.^11^ Previous studies using either radioligands^60^ or confocal imaging^61^ have shown that the A_1_AR undergoes ligand-induced internalization in response to agonist stimulation and, more recently, this signaling response has been monitored and quantified in real-time through the NanoBiT complementation assay.^62^ The NanoBiT (NanoLuc Binary)^63^ technology consists of a NanoLuciferase (NLuc)^64^ luciferase split into two segments, a large 18 kDa fragment, namely, LgBiT and a short peptide (11-amino-acid sequence), namely, HiBiT which displays very high affinity (*K*_D_ = 700 pM) for the LgBiT. Complementation between LgBiT and HiBiT reconstitutes the full-length NanoLuc luciferase, which is luminescent in the presence of the substrate furimazine. Owing to its large size, LgBiT is cell impermeable and, therefore, can primarily complement with HiBiT-tagged receptors expressed at the cell membrane, thereby enabling the monitoring of the decay in the luminescence signal as a result of the loss of receptors (HiBiT-tagged A_1_ARs) from the cell surface. Accordingly, to assess that the LD labeling reaction did not prevent access to the ligand-binding site of the A_1_AR and the functional response of the receptor was unaltered, we monitored the NECA-stimulated internalization response, following fluorophore transfer, of HiBiT-A_1_ARs expressed at the plasma membrane of living cells. Following the labeling with 1 μM of **11** (SulfoCy5) for 1 h and subsequent washing, treatment of the HiBiT-A_1_AR HEK293T cells with the agonist adenosine-5-*N*-ethylcarboxamide (NECA) produced a concentration-dependent reduction of the luminescence signal, which was consistent with an internalization response and resulted in an estimated pEC_50_ = 5.53 ± 0.15, *n* = 4 for NECA (Figure 5). Moreover, NECA-mediated HiBiT-A_1_AR internalization could be competitively inhibited by cotreatment of the cells with 10 nM DPCPX. The NECA-mediated response in the presence and absence of 10 nM DPCPX could be analyzed using the Schild equation, thereby yielding an estimated p*K*_B_ = 8.58 ± 0.13, *n* = 4 for DPCPX. The functional affinity measurements of both NECA and DPCPX were comparable to values acquired in previous studies^23,54,62^ and were not significantly different (*P* > 0.05, unpaired *t* test) from the values obtained under the same experimental conditions with unlabeled HiBiT-A_1_AR HEK293 cells (NECA pEC_50_ = 5.79 ± 0.06, *n* = 4; DPCPX p*K*_B_ = 8.97 ± 0.14, *n* = 4) (Figure 5). These results demonstrate that covalent SulfoCy5-labeling of HiBiT-A_1_ARs by **11** does not hinder the ability of additional adenosine ligands to access the A_1_AR binding site and the receptor retains its functionality following the ligand-directed labeling reaction.

**Figure 6:**
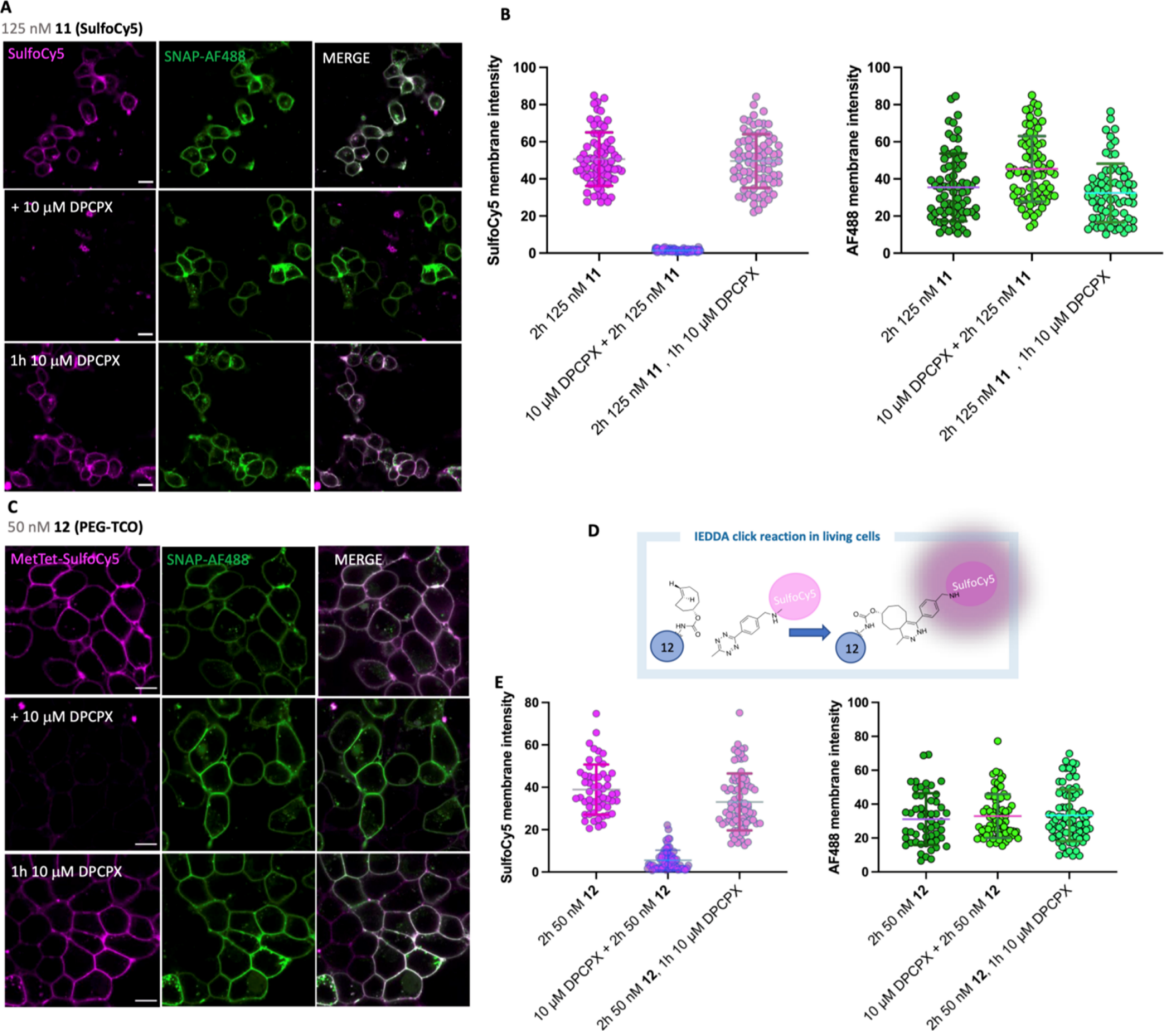
Live-cell confocal microscopy studies of (A) SulfoCy5-conjugated **11** (125 nM) and (C) PEG-TCO-conjugated **12** (50 nM) labeling SNAP-hA_1_AR HEK293T cells at 37 °C in the absence (A and C, top frames) and presence (pretreatment) of (A and C, middle frames) 10 μM DPCPX. Cells were labeled with membrane impermeable SNAP-AF488 for 30 min prior to treatment with **11** and **12** for 2 h, respectively, with the latter followed by the addition of 1 μM methyltetrazine (Met-Tet) SulfoCy5 for 5 min prior to the acquisition of the single equatorial images. (A) and (C) (bottom frames) cells were treated for 1 h with 10 μM DPCPX after the ligand-directed labeling reactions with **11** and **12** had occurred (2 h). For all the conditions, left-hand frames represent the SulfoCy5 channel (magenta), middle frames are the SNAP-hA_1_AR AF488 (green) channel, and right-hand frames represent merged images of both channels, with white indicating the overlap of magenta and green. (B, E) Fluorescence intensity plots (16-bit) were generated for both SulfoCy5 and AF488 channels by hand drawing the region of interest (ROI) in images obtained as indicated in (A) and (C) using ImageJ (FIJI). Data points represent membrane fluorescence signals measured from ROI drawn in each cell, and error bars represent mean ± standard deviation (SD). Images are representative of images obtained from three independent experiments from which 72 to 83 cells were analyzed. Scale bars are = 13 μm for (A) and 10 μm for (C). (D) Schematic representation of the biorthogonal IEDDA reaction between the TCO group (covalently attached to the hA_1_AR by ligand-directed chemistry) and SulfoCy5-conjugated methyltetrazine (MetTet-SulfoCy5). Following the click IEDDA reaction, the formation of the 4,5-dihydropyridazine cycloadduct increases the fluorescence intensity of the fluorophore conjugated to the tetrazine moiety.

Having investigated the molecular pharmacology of **11** and **12** in a series of in vitro assays, we sought to apply the LD probes for studying A_1_AR localization and dynamics in living cells. To this end, LD probes **11** and **12** were initially used in confocal microscopy studies to test their ability to label and allow visualization of the SNAP-hA_1_AR in living HEK293T cells (Figure 6). Clear fluorescent labeling of SNAP-hA_1_AR localized at the plasma membrane was observed upon treatment with 125 nM **11** (SulfoCy5) and 50 nM **12** (PEG-TCO) for 2 h, and the latter reacted with SulfoCy5-conjugated methyltetrazine (MetTet) by click IEDDA reaction to allow fluorescence detection.^38^ With both probes, the fluorescence labeling colocalized with SNAP-hA_1_AR labeled with AF488 (Figure 6A,C top frames). Pretreatment of the cells with 10 μM DPCPX prevented the labeling of SNAP-hA_1_AR by **11** and **12**, demonstrating that the labeling reaction was specific for SNAP-hA_1_AR (Figure 6A,C middle frames). The addition of 10 μM DPCPX to SNAP-hA_1_AR HEK293T cells prelabeled with **11** and **12** for 2 h, did not change their fluorescence intensities (Figure 6A,C, bottom frames and Figure 6B,E, *P* > 0.05 comparing the measured fluorescence intensities of **11** and **12** with or without 10 μM DPCPX for 1 h, two-way analysis of variance (ANOVA)). Moreover, treatment of nontransfected HEK293 cells (which do not express the A_1_AR) with **11** did not produce any detectable fluorescent signal, further demonstrating the specificity of the probe for labeling the hA_1_AR and its low level of nonspecific binding and nonspecific fluorophore transfer to bystander proteins (Figure S2). Taken together, these results showed that **11** and **12** allowed covalent labeling and visualization of SNAP-hA_1_AR expressed in living HEK293T cells.

**Figure 7:**
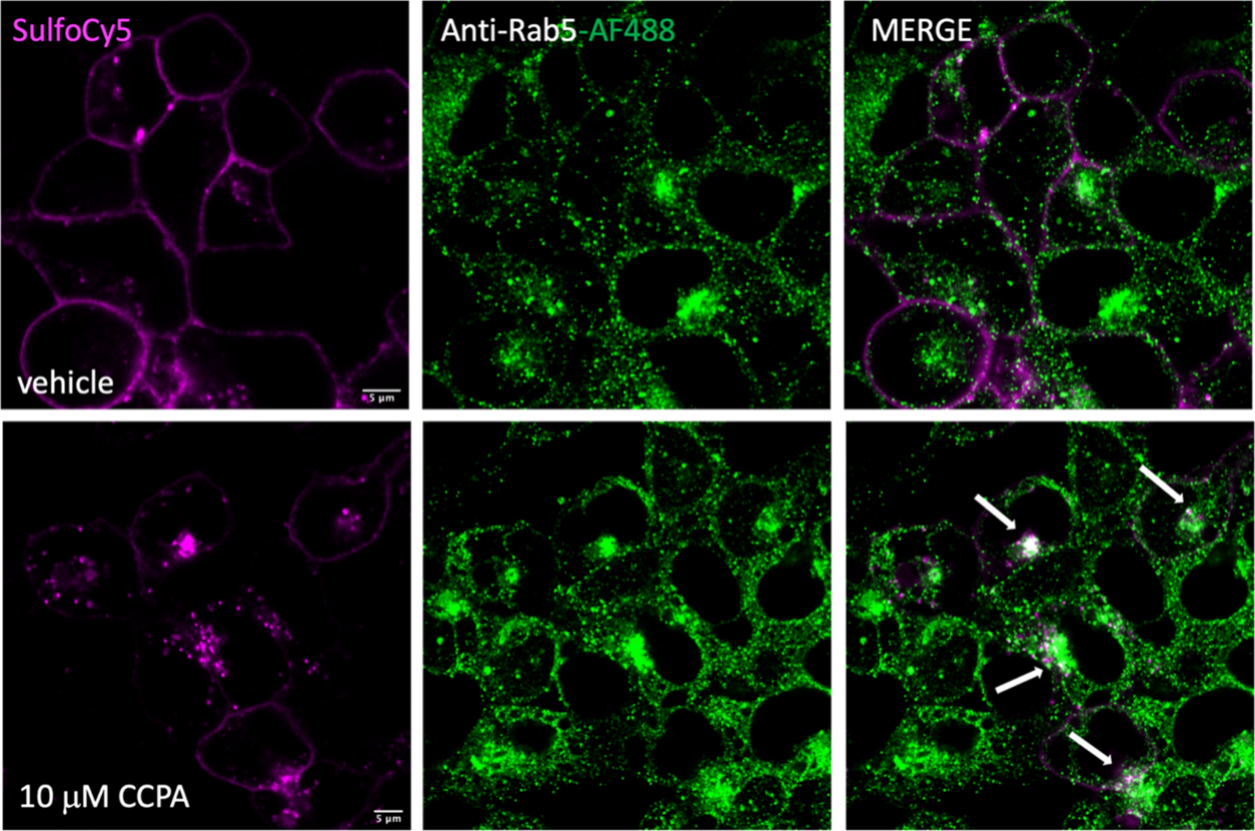
Confocal microscopy images of SulfoCy5-bioconjugated SNAP-A_1_AR HEK293T cells by probe **11** treated with the A_1_AR-selective agonist CCPA (bottom frames) or vehicle (top frames) for 2 h. Cells were immunolabeled with the Anti-Rab5 monoclonal antibody and visualized by treatment with AF488-conjugated secondary antibody (middle frames, green channel). Right-hand frames show merged images of both magenta and green channels. The white arrows in the bottom right-hand side frame show areas where colocalization of intracellular SulfoCy5-labeled A_1_AR populations with endosomal Rab5 compartments was observed. The confocal images are representative of images acquired from three independent experiments. Scale bar: 5 μm.

A natural concern would be the potential for the covalently transferred fluorophore to prevent access of ligands to the orthosteric binding site of the labeled receptor. To address this question, we employed probe **11** to visualize the internalization pathway of the hA_1_AR by confocal microscopy. To this end, SNAP-hA_1_AR HEK293T cells were treated with **11** (30 nM) for 2 h, then washed and treated with vehicle (Hank’s balanced salt solution, HBSS) or with the A_1_AR-selective agonist 2-chloro-*N*^6^-cyclopentyladenosine (2-CCPA) (10 μM) for 2 h. Confocal images revealed that, under basal conditions (vehicle), SulfoCy5-labeled SNAP-A_1_AR remained primarily localized at the plasma membrane (Figure 7, top left-hand frame). Upon stimulation of the SNAP-A_1_AR with 10 μM CCPA, localization of SNAP-A_1_AR at the plasma membrane was markedly reduced and high levels of fluorescence signals were detected in intracellular compartments (Figure 7, bottom left-hand frame). Indeed, areas of internalized SulfoCy5-labeled SNAP-A_1_AR colocalized with intracellular Rab5-positive early endosomes (Figure 7, bottom right-hand frame). These results were consistent with previous studies which provided insights into the trafficking mechanisms of the A_1_ARs in different cell types.^65,66^ Moreover, these data further confirmed results obtained from the NanoBiT internalization assay (Figure 5), supporting the theory that the SulfoCy5 labeling of the A_1_AR by probe **11** did not alter the functionality of the receptor. Taken together, these data suggest that the ligand-directed bioconjugation of A_1_AR with SulfoCy5 by **11** could represent a useful technology to probe the molecular mechanisms of the internalization pathway of the A_1_AR following agonist treatment.

**Figure 8:**
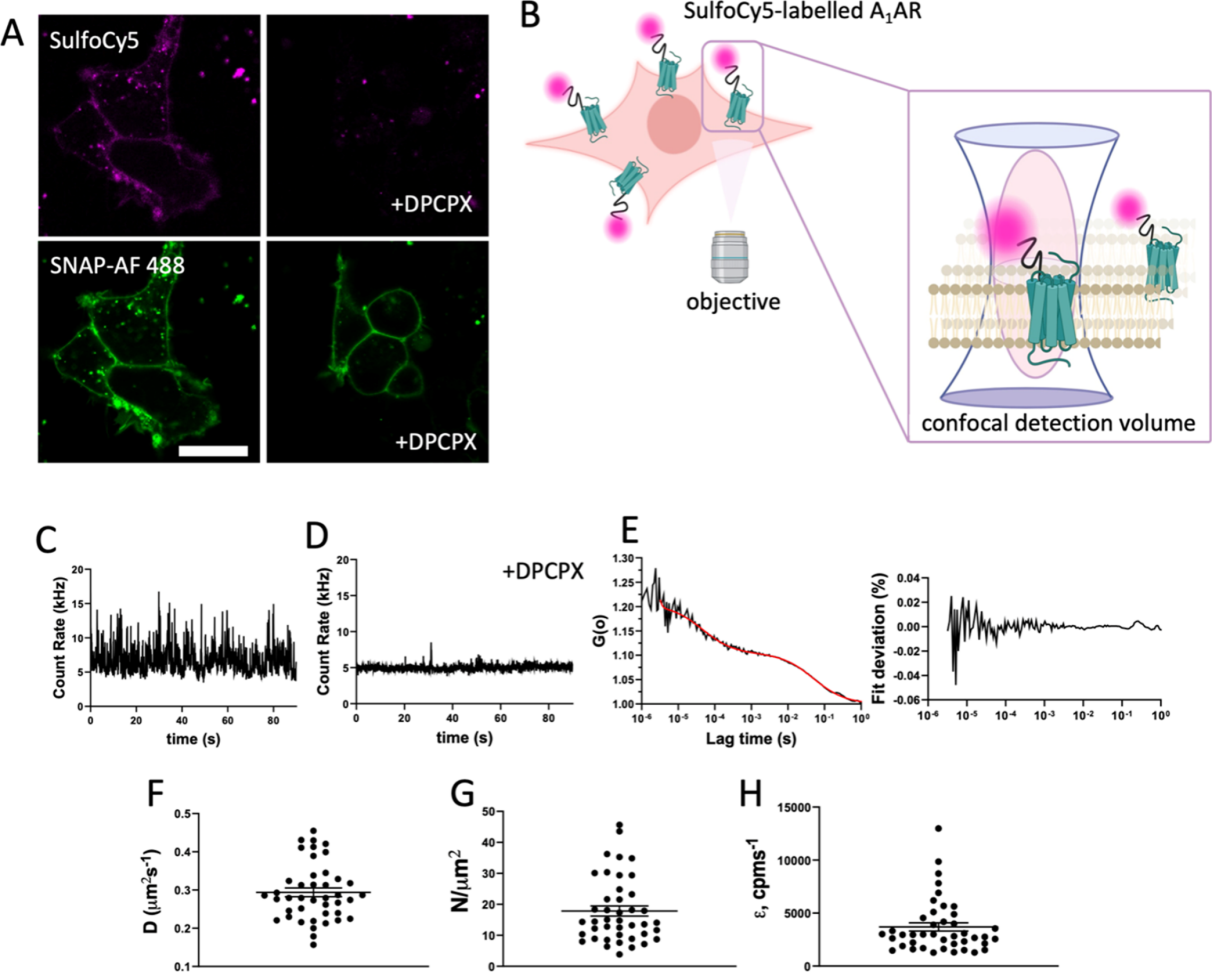
Fluorescence correlation spectroscopy studies of SulfoCy5-bioconjugated A_1_AR **11** on SNAP-A_1_AR HEK293 cells. (A) Confocal imaging of probe **11** (100 nM) and SNAP-AF488 (100 nM), used to aid membrane placement, scale bar represents 20 μm. (B) Illustration of the placement of the confocal observation volume used to record fluorescence fluctuations as SulfoCy5-labeled species diffuse within the lipid bilayer of the cell membrane; figures are prepared in BioRender (www.biorender.com). (C) Fluorescence fluctuation trace recorded on the apical membrane of SNAP-A_1_AR HEK293T cells treated with **11** alone and after preincubation with DPCPX (D, 10 μM). (E) Autocorrelation curves constructed from fluorescence traces (C, D), fit with a 1 × 3D 1 × 2D diffusion model (red line), with deviation from fit (right). (F) Diffusion coefficients (*D*, μm^2^/s), (G) particle number (*N*, μm^2^) and (H) molecular brightness (ε, cpm/s) from individual FCS measurements from 42 individual cells taken over 5 independent experiments. Mean ± SEM values of all measurements are shown by line and error bars.

In order to study receptor dynamics within their native cellular environment, where physiological expression levels can be very low, it is often necessary to employ sensitive advanced microscopy applications that maximize the detected signal-to-noise of the probe against background fluorescence.^8^ To this end, we employed fluorescence correlation spectroscopy (FCS) to investigate the dynamics of SulfoCy5-bioconjugated A_1_AR (Figure 8). FCS measures fluorescent fluctuations within a diffraction-limited confocal observation volume, ∼0.25 fL, which can be placed on the cell membrane (Figure 8B). Autocorrelation analysis of these fluctuations can be used to resolve fluorescently diffusing species and determine their average diffusion coefficient and concentration.^67,68^ Average molecular brightness can also be determined through analysis of the fluctuation traces with a photon counting histogram (PCH) analysis. FCS is especially sensitive to low expression systems as the amplitude of the autocorrelation curve (Figure 8E) is inversely proportional to the species concentration and the small observation volume maximizes the signal-to-noise ratio.

The suitability of probe **11** for use with FCS was initially tested by recording 10 × 10 s trace reads in solution (100 nM in HBSS) (Figure S4). Autocorrelation curves constructed from trace reads were fitted with a single component three-dimensional (3D) diffusion model, giving a dwell time of 117.6 ± 17.6 μs, which, after calibration of the observation volume with Cy5, gives a diffusion coefficient, *D*, of 142.7 ± 17.3 μm^2^/s. PCH analysis could also be performed with a 1-component fit confirming that probe **11** in solution exists as a single species with no aggregation.

SNAP-hA_1_AR HEK293T cells were treated with **11** (100 nM) for 90 min, then washed extensively in buffer (HBSS) to minimize unbound probe (Figure 8A). Paired cells were preincubated in 10 μM DPCPX for 30 min prior to the addition of the probe. Membrane placement of the confocal observation volume was performed using the A_1_AR-bound surface SNAP-AF488, exploiting the fact that the A_1_ receptor is largely localized in the cell membrane, which can be localized by the peak of an intensity point Z-scan. This workflow allowed us to normalize placement where no **11** binding could be observed by standard confocal imaging in the DPCPX-treated cells. Sequential 3 × 30 s trace reads taken on the apical membrane of cells treated with **11** alone displayed clear fluorescence fluctuations on the apical membrane, which represented SulfoCy5 species diffusing through the confocal observation volume (Figure 8C). Autocorrelation analysis of each summed trace (Figure 8E, black line) was fitted with a diffusion model (red line) that described a fast 3D component, confined to 20–200 μs, representing free unbound **11** and a slower 2D component (55.4 ± 1.93 ms), which represented SulfoCy5 bound A_1_AR. FCS reads from cells treated with DPCPX prior to the addition of **11** displayed much reduced fluorescence intensities of ∼5 kHz (Figure 8D). The signal here was not significantly above the background, and an autocorrelation curve could not be defined (Figure S4). As SulfoCy5 is a hydrophilic fluorophore, it would be unlikely to persist in lipid-rich membranes, unless bound to a constituent component of the membrane. Calibration of the beam path with Cy5 in solution provided the radius of the diffraction-limited observation volume on each experimental day, allowing the determination of the diffusion coefficient of SulfoCy5-A_1_AR as 0.29 ± 0.01 μm^2^/s (Figure 8F). The measured diffusion coefficient (*D*) for SulfoCy5-A_1_AR is typical of FCS-based measurements of GPCRs expressed in HEK293 cell lines that we have previously studied, such as the β_2_AR^69^ and μ-opioid receptors.^70^ Average expression level detected in cells labeled with **11** was 17.9 ± 1.62 particles per μm^2^ (range 4–45), illustrating the low levels of detection possible with FCS to explore dynamics on the nanoscale (Figure 8G). The average molecular brightness as determined by a 1-component PCH fit was 3692 ± 390 counts per molecule per second (Figure 8H). Diffusion speed, expression level, and molecular brightness all displayed a level of heterogeneity typical of the heterogeneity within a cell population.

Coupling the SulfoCy5-bioconjugation of A_1_AR with FCS provides a sensitive technique to assess the spatiotemporal dynamics of this membrane protein in real-time in live cells and at physiologically relevant expression levels.

**Figure 9:**
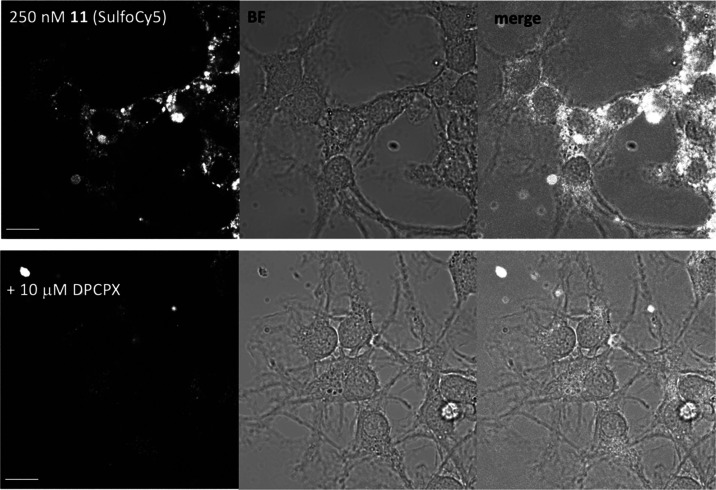
Live-cell confocal imaging of dorsal root ganglion (DRG) neurons labeled with SulfoCy5 by ligand-directed chemistry using probe **11** (250 nM). Cells were labeled in the presence (bottom frames) or absence (top frames) of 10 μM DPCPX. Left-hand side frames represent the SulfoCy5 channel, middle frames represent brightfield and right-hand side frames represent merged images from both channels. Images are representative of images acquired in four independent experiments. Scale bar: 10 μm.

Finally, we evaluated the utility of ligand-directed probe **11** to selectively label and visualize the localization and distribution of endogenously expressed A_1_AR in a physiologically relevant system. Studies have shown that activation of A_1_ARs expressed on peripheral nociceptive neurons produces analgesic effects,^14,17,71^ thereby supporting the clinical potential of targeting the A_1_ARs for the treatment of neuropathic pain. Dorsal root ganglion (DRG) neurons are sensory cells involved in the transmission of pain and they are reported to express the A_1_AR^72−75^ along with its cognate adenosine A_2A_AR^76−78^ and A_3_AR^74,79^ receptor subtypes. Accordingly, live DRG neurons isolated from adult (10–12 weeks) Sprague-Dawley rats were treated with probe **11** in the presence or absence of the A_1_AR-selective antagonist DPCPX (10 μM) (Figure 9). Confocal imaging of live DRGs showed SulfoCy5-labeling, with negligible fluorescence signal detected following pretreatment of the cells with DPCPX under the same experimental conditions. In parallel experiments, pretreatment of the cells with 10 μM A_2A_AR-selective antagonist ZM241385 did not prevent the SulfoCy5 labeling of the neural cells (Figure S5), thereby supporting the potential of the probe for selectively labeling endogenously expressed A_1_ARs.

## Discussion and Conclusions

The work presented herein described the rational design and development of a ligand-directed chemistry approach for the bioconjugation of the adenosine A_1_AR receptor with functional probes in living cells. The combination of cocrystal structures of xanthines-A_1_AR complexes with molecular modeling studies, as well as previously reported SAR profiles of xanthine-based A_1_ ligands^25,58^ and fluorescent probes,^23^ underpinned the rational design of two ligand-directed (LD) covalent probes encompassing 8-bicyclo[2.2.2]octylxanthine based 3-fluorophenyl esters. These were conjugated with a SulfoCy5 fluorophore (**11**) and, to expand the scope of application of the probes, a polyethylene glycol-linked *trans*-cyclooctene (PEG-TCO, **12**) clickable handle that could undergo the IEDDA reaction with a different range of fluorophore-functionalized tetrazines. The molecular pharmacology of phenyl esters **11** and **12** against the A_1_AR, of both human and rat species, was quantified in living cells through NanoBRET ligand-binding assays, thereby revealing that both probes displayed a high A_1_AR ligand-binding affinity (Figure 3 and Table 1). Moreover, specific labeling of human and rat NL-tagged A_1_AR by **11** (SulfoCy5) could not be displaced following the addition of high concentrations of the competitive A_1_AR antagonist DPCPX (10 μM), suggesting covalent transfer of the fluorophore to a nucleophilic residue in proximity to the ligand-binding site of A_1_AR (Figure 3C,F). Specifically, covalent labeling of A_1_AR by **11** and **12** could be confirmed by SDS-PAGE experiments.

The ligand-binding properties of **11** and **12** against the other three adenosine receptor subtypes (NL-A_2A_AR, NL-A_2B_AR, and NL-A_3_AR) was also quantified by NanoBRET ligand-binding assays, revealing that, overall, both probes displayed a reasonable selectivity profile for binding NL-A_1_AR (Figure 4 and Table 1). Importantly, the ligand-directed labeling of the A_1_AR by **11** did not affect the normal functionality of the receptor as demonstrated by the NanoBiT internalization assay. Indeed, results showed that additional adenosine ligands could access the ligand-binding site of A_1_AR as their ligand-binding properties could be quantified and were in agreement with those measured in wild-type (unlabeled) receptors (Figure 5).

LD probes **11** and **12** were successfully applied in confocal microscopy studies for the specific labeling of SNAP-hA_1_AR in living cells. Specifically, upon treatment of the cells with **12**, the corresponding covalently transferred PEG-TCO handle reacted with SulfoCy5-substituted methyltetrazine (SulfoCy5-MetTet) following the IEDDA reaction (Figure 6D), thereby allowing visualization of SNAP-hA_1_AR by a two-step mechanism. In principle, a two-step labeling approach allows for a single ligand to direct covalent transfer of a small reactive clickable handle to the receptor (first step). This reactive handle can then specifically react with a different range of tetrazine-conjugated probes depending on the final application (second step). With both **11** and **12**, the labeling reaction was prevented by pretreatment of the cells with a high concentration (10 μM) DPCPX demonstrating that the probes necessitated to specifically engage with the SNAP-hA_1_AR to promote covalent transfer of the functional probes. Moreover, following the labeling reaction, the SulfoCy5 fluorescent signal remained unaffected upon treatment with 10 μM DPCPX for 1 h (Figure 6).

Moreover, LD probe **11** was successfully applied in FCS studies, thereby enabling the real-time quantification of receptor spatiotemporal dynamics, such as changes in the movement (diffusion coefficient; *D*), number (particle number; *N*), and clustering (molecular brightness; ε) of SulfoCy5-bioconjugated SNAP-hA_1_AR within small microdomains of the plasma membrane. Hence, this data demonstrated the suitability of the probe to be coupled with more advanced and sensitive imaging techniques.

Understanding the molecular mechanisms and differences underlying the physiological effects mediated by proteins in their native, live-cell environment is an essential prerequisite for the successful development of better-targeted and efficacious therapeutics. Accordingly, the availability of technologies, which allow the monitoring of proteins’ native localization, functions, and dynamics is of great utility as they may provide new research avenues and opportunities for progressing fundamental science particularly those that bypass the need to genetically modify the protein of interest.^1,6^ The adenosine A_1_AR receptor is a membrane-bound receptor that is thoroughly distributed in the human body. Particularly, the activation of A_1_AR expressed in nociceptive neurons (e.g., DRGs) has been reported to produce “potent” analgesic effects, hence targeting the A_1_AR provides potential therapeutic opportunities for treating neuropathic pain, encompassing a possible and safer alternative to opioid-based treatments. Notwithstanding, the cellular mechanisms underlying these A_1_AR-mediated functional responses are poorly understood. The ligand-directed labeling technology designed here successfully enabled the monitoring of the localization of the A_1_AR endogenously expressed in living DRG neurons. Accordingly, by allowing the permanent bioconjugation of the A_1_AR with functional probes in its native live-cell environment, this A_1_LD labeling platform may shed new light into the underlying molecular mechanisms of the A_1_AR-mediated analgesic effect in nociceptive neurons, thereby providing opportunities to study ligand-binding, receptor trafficking, and functional response in a clinically relevant system and aid into the discovery of new nonopioids analgesics.

The new probes reported herein were thoroughly characterized and were amenable to a broad range of fluorescence-based techniques. Moreover, the new ligand-directed technology developed for the A_1_AR will be a valuable research platform for the wider scientific community to aid in understanding of function and spatiotemporal dynamics of A_1_AR in physiologically relevant systems and identify new mechanisms for therapeutic intervention.

## Experimental Section

### General Methods and Chemistry

Chemicals and solvents of analytical and HPLC grade were purchased from commercial suppliers and used without further purification. SulfoCyanine5-CO_2_H, and SulfoCyanine5 tetrazine (Met-TetCy5) were purchased from Lumiprobe (Germany). *trans*-Cyclooctene-NHS (TCO-NHS) ester was obtained from Jena Biosciences (Germany). All reactions were carried out at ambient temperature, unless otherwise stated. Reactions were monitored by thin-layer chromatography on commercially available silica-precoated aluminum-backed plates (Merck Kieselgel 60 F^254^). Visualization was under UV light (254 and 366 nm), followed by staining with iodine, ninhydrin, or KMnO_4_ dips. Flash column chromatography was performed using silica gel 60, 230–400 mesh particle size (Sigma-Aldrich). Automated flash column chromatography was performed on a InterchimPuriflash 4100 system (PF4100-250) equipped with a dual-wavelength DAD UV detector (200–600 nm) using either silica high-performance (HP) 50 μm, or C18-HP (30 μm) cartridges. Methods were developed and run using Interchim Flash (ver:V5.1c.09) software. NMR spectra were recorded on a Bruker-AV 400. ^1^H NMR spectra were recorded at 400.13 MHz and ^13^C NMR spectra at 101.62 MHz. All ^13^C NMR spectra are ^1^H broadband decoupled. Solvents used for NMR analysis (reference peaks listed) were CDCl_3_ (δH = 7.26 ppm, δC = 77.16), MeOD_4_ (δH = 3.34 ppm, δC = 49.86), and DMSO-*d*_6_ (δH = 2.50 ppm, δC = 40.45) supplied by Sigma-Aldrich (U.K.). Chemical shifts (δ) are recorded in parts per million (ppm), and coupling constants are recorded in Hz. The following abbreviations are used to describe signal shapes and multiplicities; singlet (s), doublet (d), triplet (t), quadruplet (q), broad (br), dd (doublet of doublets), ddd (double doublet of doublets), dtd (double triplet of doublets), and multiplet (m). Processing of the NMR data was carried out using NMR software Mnova 12.0.4. Liquid chromatography-mass spectrometry (LC-MS) spectra were recorded on a Shimadzu UFLCXR system coupled to an Applied Biosystems API2000 and visualized at 254 nm (channel 1) and 220 nm (channel 2). LC-MS was carried out using a Phenomenex Gemini-NX-C18 110A column (50 mm × 2 mm × 3 μm) at a flow rate 0.5 mL/min over a 5 min period (Method A). High-resolution mass spectra (HRMS) were recorded on a Bruker microTOF mass spectrometer using MS electrospray ionization (ESI) operating in positive ion mode or on an Agilent 6624 TOF LC-MS spectrometer coupled to an Agilent 1290 Infinity system (Agilent, Palo Alto, CA). All data were acquired and reference mass corrected via a dual-spray electrospray ionization (ESI) source. Acquisition was performed using Agilent Mass Hunter Data Acquisition software version B.05.00 Build 5.0.5042.2, and analysis was performed using Mass Hunter Qualitative Analysis version B.05.00 Build 5.0.519.13. RP-HPLC was performed on a Waters 515 LC system and monitored using a Waters 996 photodiode array detector at wavelengths between 190 and 800 nm. Spectra were analyzed using Millenium 32 software. Semipreparative HPLC was performed using YMC-Pack C8 column (150 mm × 10 mm × 5 μm) at a flow rate of 5.0 mL/min using a gradient method of 30–95% B over 24 min (solvent A = 0.01% formic acid in H_2_O, solvent B = 0.01% formic acid in CH_3_CN) (Method B). Analytical RP-HPLC was performed using a YMC-Pack C8 column (150 mm × 4.6 mm × 5 μm) and a Phenomenex Gemini NX-C18 column (250 mm × 4.6 mm × 5 μm) at a flow rate of 1.0 mL/min. The retention time of the final product is reported using a gradient method of 10–90% solvent B in solvent A over 30 min. (Solvent A = 0.01% formic acid in H_2_O, solvent B = 0.01% formic acid in CH_3_CN) (Method C). The final products were one single peak and >95% pure.

### General Procedure A: Amide Coupling

A solution of respective carboxylic acid (1.0 equiv) in anhydrous DMF (0.5 mL) was stirred with DIPEA (1.10 equiv) and COMU (1.10 equiv) for 5 min at rt. A solution of respective amine (1.10 equiv) was added to the reaction mixture, and the resulting solution was stirred at rt for 15–60 min. Upon completion of the reaction, the solvent was evaporated to dryness. The residue was taken up in EtOAc and washed with 1 M aq. HCl, sat. NaHCO_3_, and brine. The organic layer was collected, dried over anhydrous MgSO_4_, filtered, and evaporated to dryness. The resulting residue was purified by flash column chromatography, as indicated.

### General Procedure B: Phenyl Ester Synthesis

To a solution of respective carboxylic acid (1.0 equiv) in anhydrous DMF (0.25 mL), was added a solution of 2-bromo-1-ethyl-pyridinium tetrafluoroborate (BEP) (0.8 equiv) and DIPEA (10 mg) in anhydrous DMF (0.25 mL). After stirring for 15 min in the dark, this solution was added to a solution of respective 3-fluoro-4-hydroxybenzamido congener (1.0 equiv) in anhydrous DMF (0.3 mL). After stirring under the exclusion of light for 16 h, the solvent was evaporated to dryness under high vacuum. Purification by semipreparative RP-HPLC and subsequent lyophilization yielded the pure ligand-directed label as a bright blue or off-white solid (for all evaporation steps the water bath was set to <32 °C).

### General Procedure C: Deprotection of *tert*-Butyl Carbamate (Boc-Group)

To the respective Boc-protected amine (1 equiv) was added excess 4 M HCl in dioxane (1 mL). The resulting mixture was stirred at rt for 30 min. Upon completion of the reaction, monitored by thin layer chromatography (TLC) and LC/MS, the solvent was evaporated to dryness to give the corresponding amine as its HCl salt, which was used for the subsequent step without further purification.

#### Preparation of 1,3-Dibutylurea (**1**)

Butylamine (7.68 mL, 78 mmol, 1.10 equiv) was dissolved in anhydrous tetrahydrofuran (THF) (20 mL) and the solution was cooled to 0 °C. Butyl isocyanate (7.95 mL, 71 mmol, 1.0 equiv) was added dropwise, and the reaction was stirred at rt. The resulting mixture was stirred at rt for 4 h. Evaporation of the solvent in vacuo yielded the title product as an off-white solid (12 g, quantitative). ^1^H NMR (400 MHz, DMSO-*d*_6_) δ 5.72 (t, *J* = 5.7 Hz, 2H), 2.95 (td, *J* = 6.8, 5.6 Hz, 4H), 1.37–1.19 (m, 8H), 0.85 (t, *J* = 7.2 Hz, 6H).^13^C NMR (101 MHz, DMSO-*d*_6_) δ 158.1, 38.9, 32.2, 19.5, 13.7.

#### Preparation of 6-Amino-1,3-dibutylpyrimidine-2,4(1*H*,3*H*)-dione (**2**)

1,3-Dibutylurea (6 g, 35 mmol, 1 equiv) was reacted with cyanoacetic acid (3.26 g, 38 mmol, 1.10 equiv) in Ac_2_O (20 mL). The resulting mixture was heated to 85 °C for 2 h. Afterward, the solution was concentrated to dryness (water bath set to 80 °C) until a brown syrup appeared. 3× 4 mL of H_2_O were added to remove excess of Ac_2_O. 10 mL of H_2_O was added and the residue was basified with 70% NaOH (four drops) to obtain a precipitate which was filtered off. The filtrate was collected and concentrated to dryness. The resulting residue was recrystallized from hot EtOH/H_2_O to obtain fine pale-yellow crystals (5.332 g, 64%). LC-MS *m*/*z* calcd for C_12_H_22_N_3_O_2_ [MH]^+^: 240.17, found 240.7 *t*_R_ = 2.65 min (Method A). ^1^H NMR (400 MHz, DMSO-*d*_6_) δ 6.75 (s, 2H), 4.64 (s, 1H), 3.76 (t, *J* = 7.2 Hz, 2H), 3.69 (t, *J* = 7.2 Hz, 2H), 1.52–1.38 (m, 4H), 1.25 (m, *J* = 20.6, 7.4 Hz, 4H), 0.87 (q, *J* = 7.4 Hz).^13^C NMR (101 MHz, DMSO-*d*_6_) δ 161, 154.2, 151.2, 75.1, 41.5, 29.7, 29.7, 19.6, 19.3, 13.7, 13.7.

#### Preparation of 6-Amino-1,3-dibutyl-5-nitrosopyrimidine-2,4(1*H*,3*H*)-dione (**3**)

1,3-Diisobutyl-6-amino uracil (3.577 mg, 15 mmol, 1.0 equiv) was dissolved in 50% acetic acid (50 mL) and the resulting solution was heated to 57 °C. To the mixture was added NaNO_2_ (1.238 g, 18 mmol, 1.20 equiv) over 15 min. The solution from colorless turned pink and a pink precipitate formed. The reaction mixture was stirred at rt for 1 h. The precipitate was collected, washed with cold water, and dried to get the pure product as a pink solid (1.625 mg, 41%). LC-MS *m*/*z* calcd for C_12_H_21_N_4_O_3_ [MH]^+^: 269.16, found 269.10 *t*_R_ = 2.71 min (Method A).^1^H NMR (400 MHz, DMSO-*d*_6_) δ 13.17 (s, 1H), 9.10 (s, 1H), 3.89 (t, *J* = 7.3 Hz, 2H), 3.81 (t, *J* = 7.7 Hz, 2H), 1.61–1.52 (m, 2H), 1.51–1.43 (m, 2H), 1.38–1.26 (m, 4H), 0.90 (dt, *J* = 11.4, 7.3 Hz, 6H).^13^C NMR (101 MHz, DMSO-*d*_6_) δ 159.9, 149.0, 145.4, 138.9, 41.0, 40.6, 29.5, 28.4, 19.6, 19.2, 13.7, 13.6.

#### Preparation of 5,6-Diamino-1,3-dibutylpyrimidine-2,4(1*H*,3*H*)-dione (**4**)

To a stirring solution of 6-amino-5-nitroso-1,3-dibutylpyrimidine-2,4(1*H*,3*H*)-dione (0.700 g, 2.61 mmol, 1 equiv) in 12.5% aqueous ammonium hydroxide solution (25 mL) at 60 °C was added sodium dithionite (3 equiv) until a colorless solution formed (30 min). After cooling to rt, the product was extracted with dichloromethane (DCM) (4× 20 mL). The combined organics were dried over anhydrous Na_2_SO_4_, filtered, and evaporated to dryness to give the title product as a pale-yellow solid/wax (0.529 g, 80%). LC-MS *m*/*z* calcd for C_12_H_23_N_4_O_2_ [MH]^+^: 255.18, found 255.20 *t*_R_ = 2.20 min (Method A). ^1^H NMR (400 MHz, CDCl_3_) δ 5.16 (s, 2H), 3.91 (t, *J* = 7.4 Hz, 2H), 3.87 (t, *J* = 8.4, 6.8 Hz, 2H), 3.17 (s, 2H), 1.65 (p, *J* = 7.3 Hz, 2H), 1.58 (p, *J* = 7.3 Hz, 2H), 1.41 (h, *J* = 7.1 Hz, 2H), 1.35 (h, *J* = 7.1 Hz, 2H), 0.94 (dt, *J* = 15.6, 7.3 Hz, 6H).^13^C NMR (101 MHz, CDCl_3_) δ 161.5, 150.4, 148.6, 94.7, 43.2, 41.6, 30.5, 30.2, 20.4, 20.2, 14.0, 13.8.

#### Preparation of 4-(1,3-Dibutyl-2,6-dioxo-2,3,6,7-tetrahydro-1*H*-purin-8-yl)bicyclo[2.2.2]octane-1-carboxylic Acid (**5**)

To a solution of 5,6-diamino-1,3-diisobutylpyrimidine-2,4(1*H*,3*H*)-dione (0.297 g, 1.10 mmol, 1.10 equiv) (**4**) in DMF (5 mL) was added a solution of 4-(methoxycarbonyl)bicyclo[2.2.2]octane-1-carboxylic acid (0.225 g, 1.06 mmol, 1.0 equiv), DIPEA (0.37 mL, 2 equiv), and COMU (0.499 g, 1.10 mmol, 1.10 equiv). The resulting mixture was stirred at rt for 15 min. The mixture was diluted with cold H_2_O and extracted with EtOAc. The organic layer was separated and washed with 10% citric acid. The organics were collected and washed with sat. NaHCO_3_, brine, dried over MgSO_4_, filtered, and concentrated in vacuo to give methyl 4-((6-amino-2,4-dioxo-1,3-dibutyl-1,2,3,4-tetrahydropyrimidin-5-yl)carbamoyl)bicyclo[2.2.2]octane-1-carboxylate intermediate, which was used for the next step without further purification. The residue was dissolved in a mixture of propan-2-ol and 1 M KOH (2.5 mL, 4 equiv) and heated to reflux (91 °C) for 2 h. The reaction mixture was then cooled to rt and the solvent was concentrated to dryness. The yellow residue was taken up in 4 mL of water and extracted with DCM. The aqueous layer was collected and acidified with conc HCl (until pH = 3–4) and a white precipitate formed, which was collected by suction filtration and oven-dried. (0.300 g, 68% over two steps). LC-MS *m*/*z* calcd for C_22_H_33_N_4_O_4_ [MH]^+^: 417.10, found 417.10 *t*_R_ = 3.08 min (Method A). ^1^H NMR (400 MHz, DMSO-*d*_6_) δ 12.73 (br s, 1H), 3.95 (t, *J* = 7.1 Hz, 2H), 3.85 (t, *J* = 6.9 Hz, 2H), 1.91–1.83 (m, 6H), 1.79–1.72 (m, 6H), 1.62 (p, *J* = 6.9 Hz, 2H), 1.49 (p, *J* = 6.9 Hz, 2H), 1.27 (h, *J* = 7.4 Hz, 4H), 0.89 (dt, *J* = 7.3, 4.8 Hz, 6H).^13^C NMR (101 MHz, DMSO-*d*_6_) δ 178.5, 160.3, 153.9, 150.6, 147.3, 106.6, 42.3, 37.8, 33.2, 29.7, 29.6, 29.5, 27.7, 19.6, 19.3, 13.7, 13.6.

#### Preparation of *tert*-Butyl (2-(4-(1,3-Dibutyl-2,6-dioxo-2,3,6,7-tetrahydro-1*H*-purin-8-yl)bicyclo[2.2.2]octane-1-carboxamido)ethyl)carbamate (**6**)

Following general procedure A, to a stirring solution of (2,6-dioxo-1,3-dibutyl-2,3,6,9-tetrahydro-1*H*-purin-8-yl)bicyclo [2.2.2]octane-1-carboxylic acid (**5**) (300 mg, 720 μmol, 1 equiv), DIPEA (138 μL, 792 μmol, 1.10 equiv), COMU (339 mg, 792 μmol, 1.10 equiv), and DMF (1.5 mL) was added *tert*-butyl(3-aminopropyl)carbamate (0.125 mL, 792 μmol, 1.10 equiv). Purification by automated flash column chromatography using a gradient of 98:2 to 90:10 DCM/MeOH gave the title pure compound as an off-white solid (284 mg, 71%). LC-MS *m*/*z* calcd for C_29_H_47_N_6_O_5_ [MH]^+^: 599.30, found 599.30 *t*_R_ = 3.22 min (Method A). ^1^H NMR (400 MHz, MeOD_4_) δ 4.12 (t, *J* = 7.3 Hz, 2H), 3.99 (t, *J* = 7.3 Hz, 2H), 3.26 (t, *J* = 5.8 Hz, 2H), 3.19 (t, *J* = 6.0 Hz, 2H), 2.05–1.98 (m, 6H), 1.94–1.86 (m, 6H), 1.75 (p, *J* = 6.4 Hz, 2H), 1.62 (p, *J* = 6.6 Hz, 2H), 1.47 (s, 9H), 1.39 (hd, *J* = 7.3, 3.6 Hz, 4H), 0.99 (dt, *J* = 7.3, 7.3 Hz, 6H).^13^C NMR (101 MHz, MeOD_4_) δ 180.4, 162.1, 158.8, 156.0, 152.8, 149.4, 108.2, 80.2, 44.2, 42.1, 41.3, 40.7, 40.1, 34.9, 31.2, 31.0, 29.2, 28.8, 21.1, 20.8, 14.2, 14.1.

#### Preparation of *tert*-Butyl (3-((2-(4-(1,3-Dibutyl-2,6-dioxo-2,3,6,7-tetrahydro-1*H*-purin-8-yl)bicyclo[2.2.2]octane-1-carboxamido)ethyl)amino)-3-oxopropyl)carbamate (**8**)

Compound **6** (274 mg, 1.0 equiv) was treated with 4 M HCl in dioxane (1 mL) following general procedure C to yield the corresponding 3-((2-(4-(2,6-dioxo-1,3-dibutyl-2,3,6,7-tetrahydro-1*H*-purin-8-yl)bicyclo[2.2.2]octane-1-carboxamido)ethyl)amino)-3-oxopropan-1-aminium chloride intermediate (**7**) (quantitative), which was used for the next step without further purification. Following general procedure A, 3-((2-(4-(2,6-dioxo-1,3-dibutyl-2,3,6,7-tetrahydro-1*H*-purin-8-yl)bicyclo[2.2.2]octane-1-carboxamido)ethyl)amino)-3-oxo-propan-1-aminium chloride intermediate (**7**) (225 mg, 1.0 equiv) was treated with DIPEA (234 μL, 1.4 mmol, 10.0 equiv) and reacted with a solution of Boc-β-Ala-OH (85 mg, 449 μmol, 1.0 equiv) and COMU (211 mg, 494 μmol, 1.10 equiv) in DMF (1 mL). Purification by automated flash column chromatography using a gradient of 97:3 to 90:10 DCM/MeOH gave the title compound as an off-white solid (250 mg, 88%). LC-MS *m*/*z* calcd for C_32_H_52_N_7_O_6_ [MH]^+^: 630.30, found 630.30 *t*_R_ = 3.13 min (Method A). ^1^H NMR (400 MHz, MeOD_4_) δ 4.11 (t, *J* = 7.3 Hz, 2H), 3.99 (t, *J* = 6.1 Hz, 2H), 3.30 (s, 4H), 2.37 (t, *J* = 6.7 Hz, 2H), 2.05–1.98 (m, 6H), 1.94–1.87 (m, 6H), 1.74 (p, *J* = 5.4 Hz, 2H), 1.62 (p, *J* = 4.9 Hz, 2H), 1.45 (s, 9H), 1.38 (hd, *J* = 7.4, 2.5 Hz, 4H), 0.98 (q, *J* = 7.2 Hz, 6H).^13^C NMR (101 MHz, MeOD_4_) δ 179.1, 173.1, 160.8, 157.0, 154.6, 151.4, 148.1, 106.8, 78.8, 42.8, 40.7, 39.2, 38.7, 38.6, 36.7, 36.2, 33.5, 29.8, 29.8, 29.6, 27.8, 27.4, 19.8, 19.4, 12.8, 12.7.

#### Preparation of 3-((2-(4-(1,3-Dibutyl-2,6-dioxo-2,3,6,7-tetrahydro-1*H*-purin-8-yl)bicyclo[2.2.2]octane-1-carboxamido)ethyl)amino)-3-oxopropan-1-aminium Chloride (**9**)

Compound **8** (70 mg, 0.11 mmol, 1.0 equiv) was treated with 4 M HCl in dioxane (1 mL) following general procedure C to yield the title intermediate as a HCl salt (quantitative), which was used for the next step without further purification. LC-MS *m*/*z* calcd for C_27_H_44_N_7_O_4_ [MH]^+^: 530.30, found 530.30 *t*_R_ = 2.47 min (Method A).

#### Preparation of 4-(1,3-Dibutyl-2,6-dioxo-2,3,6,7-tetrahydro-1*H*-purin-8-yl)-*N*-(2-(3-(3-fluoro-4-hydroxybenzamido)propanamido)ethyl)bicyclo[2.2.2]octane-1-carboxamide (**10**)

3-Fluoro-4-hydroxybenzoic acid (16 mg, 102 μmol, 1.01 equiv) was dissolved in DMF (0.5 mL) and reacted with COMU (53 mg, 123 μmol, 1.10 equiv) in the presence of DIPEA (54 μL, 307 μmol, 3 equiv). To the resulting mixture was added a solution of *N*-(2-(3-aminopropanamido)ethyl)-4-(1,3-dibutyl-2,6-dioxo-2,3,4,5,6,7-hexahydro-1*H*-purin-8-yl)bicyclo[2.2.2]octane-1-carboxamide hydrochloride (**9**) (59 mg, 111 μmol, 1.0 equiv) in DMF (0.5 mL). The resulting solution was stirred at 90 °C for 1 h. Upon completion of the reaction monitored by LC-MS, the solvent was removed under reduced pressure, and the resulting residue was dissolved in EtOAc and washed with water (2× 10 mL). The organics were collected, dried over anhydrous MgSO_4_, filtered, and evaporated to dryness. The resulting residue was purified by automated flash column chromatography with DCM/MeOH on a gradient of 94:6 to 90:10%. The title compound was obtained as an off-white solid (39 mg, 53%). LC-MS *m*/*z* calcd for C_34_H_47_FN_7_O_6_ [MH]^+^: 668.30, found 668.30 *t*_R_ = 2.96 min, purity >99% (Method A). ^1^H NMR (400 MHz, MeOD_4_) δ 7.90, (s, 1H), 7.58 (dd, *J* = 11.9, 2.1 Hz, 1H), 7.53 (dd, *J* = 8.4, 2.2 Hz, 1H), 6.95 (t, *J* = 8.5 Hz, 1H), 4.10 (t, *J* = 7.3 Hz, 2H), 3.97 (t, *J* = 6.9 Hz, 2H), 3.62 (t, *J* = 6.7 Hz, 2H), 3.30 (s, 4H), 2.50 (t, *J* = 6.7 Hz, 2H), 2.01–1.93 (m, 6H), 1.89–1.82 (m, 6H), 1.73 (p, *J* = 6.8 Hz, 2H), 1.60 (p, *J* = 6.2 Hz, 2H), 1.37 (hd, *J* = 7.4, 3.3 Hz), 0.97 (q, *J* = 7.3 Hz).^13^C NMR (101 MHz, MeOD_4_) δ 180.5, 174.5, 168.73 (d, *J* = 2.2 Hz), 162.1, 156.0, 152.8, 152.4 (d, *J* = 241.5 Hz), 149.86 (d, *J* = 13.0 Hz), 149.4, 126.88 (d, *J* = 5.4 Hz), 125.15 (d, *J* = 3.1 Hz), 118.43 (d, *J* = 3.0 Hz), 116.32 (d, *J* = 20.1 Hz), 108.2, 79.5, 44.2, 42.1, 40.6, 40.1, 37.7, 36.9, 34.9, 31.2, 31.1, 31.0, 29.2, 21.2, 20.8, 14.2, 14.1.^19^F NMR (377 MHz, MeOD_4_) δ −138.6.

#### Preparation of 2,2-Dimethyl-4,15-dioxo-3,8,11-trioxa-5,14-diazaoctadecan-18-oic Acid

At 0 °C, to the mono-protected amine (0.200 g, 0.8 mmol, 1 equiv) in chloroform (7 mL) was added succinic anhydride (81 mg, 1 equiv). The resulting solution was warmed to rt and stirred for 12 h. The solvent was removed under reduced pressure to afford the crude product as a pale-yellow oil. Purification by automated flash column chromatography using a linear gradient of cyclohexane/(3:1 EtoAC/IPA) 65:35 to 25:75 afforded the title product as a colorless oil (120 mg, 43%). ^1^H NMR (400 MHz, CDCl_3_) δ 7.45 (s, 1H), 6.90 (s, 1H), 3.61 (s, 4H), 3.54 (q, *J* = 5.1 Hz, 4H), 3.44 (q, *J* = 5.1 Hz, 2H), 3.32 (t, *J* = 5.1 Hz, 2H), 2.70–2.63 (t, *J* = 5 Hz, 2H), 2.50 (t, *J* = 4.5 Hz, 2H), 1.45 (s, 9H).

#### Preparation of (*R*,*E*)-1-(Cyclooct-4-en-1-yloxy)-1,12-dioxo-5,8-dioxa-2,11-diazapentadecan-15-oic Acid^1^

2,2-Dimethyl-4,15-dioxo-3,8,11-trioxa-5,14-diazaoctadecan-18-oic acid was treated with 4 M HCl in dioxane (1 mL) at rt for 1 h. Removal of the solvent with high vacuum, followed by three cycles of Et_2_O evaporation, afforded the title amine as its HCl salt, which was used for the subsequent step without further purification. *trans*-Cyclooctene-NHS (TCO-NHS) (11 mg, 47 μmol, 1.0 equiv) was added to a flame-dried round-bottom flask (RBF) and was diluted with anhydrous DMF (0.7 mL) while under a stream of N_2_. To the solution was added NEt_3_ (8 equiv) followed by 2-(2-(2-(3-carboxypropanamido)ethoxy)ethoxy)ethan-1-aminium chloride (30 mg, 1.10 equiv) added in one portion. The flask was wrapped in foil and stirred under N_2_ at room temperature for 20 h. After that time the solution was diluted with H_2_O and extracted with EtOAc (2× 5 mL) in a pear-shaped flask. The aqueous layer was acidified with 6% acetic acid (until pH = 3–4) and extracted with DCM (3× 7 mL). The organic layers were combined and concentrated to dryness. Purification by automated column chromatography using a linear gradient of cyclohexane/(3:1 EtoAC/IPA) 60:40 to 15:85 afforded the title product as a colorless oil (6 mg, 36%). ^1^H NMR (400 MHz, MeOD_4_) δ 5.62 (ddd, *J* = 16.7, 10.0, 4.7 Hz, 1H), 5.49 (ddd, *J* = 16.4, 7.9, 3.2 Hz, 1H), 4.38–4.29 (m, 1H), 3.65–3.60 (m, 4H), 3.57–3.51 (m, 4H), 3.37 (t, *J* = 5.5 Hz, 2H), 3.27 (q, *J* = 5.1 Hz, 2H), 2.60 (t, *J* = 6.8 Hz, 2H), 2.49 (t, *J* = 6.9 Hz, 2H), 2.35 (dq, *J* = 9.7, 4.9 Hz, 3H), 2.04–1.88 (m, 4H), 1.80–1.68 (m, 2H), 1.66–1.55 (m, 1H).

#### Preparation of 1-(6-(4-((3-((2-(4-(1,3-Dibutyl-2,6-dioxo-2,3,6,7-tetrahydro-1*H*-purin-8-yl)bicyclo[2.2.2]octane-1-carboxamido)ethyl)amino)-3-oxopropyl)carbamoyl)-2-fluorophenoxy)-6-oxohexyl)-3,3-dimethyl-2-((1*E*,3*E*)-5-((*E*)-1,3,3-trimethyl-5-sulfoindolin-2-ylidene)penta-1,3-dien-1-yl)-3*H*-indol-1-ium-5-sulfonate (**11**)

Following general procedure B, 3-fluoro-4-hydroxybenzamido congener (**10**) (0.98 mg, 1.47 μmol, 1 equiv) was converted to the SulfoCy5 conjugate **11**. Purification by RP-HPLC (Method B) gave, after lyophilization, the title compound as a bright blue fluffy solid (1.42 mg, 75%). HRMS (TOF ES^–^) calcd for C_66_H_81_FN_9_O_13_S_2_ [MH]^−^: 1290.5385, found 1290.5345. Analytical RP-HPLC *t*_R_ = 15.72 min, purity >98% (Method C).

#### Preparation of 4-((3-((2-(4-(1,3-Dibutyl-2,6-dioxo-2,3,6,7-tetrahydro-1*H*-purin-8-yl)bicyclo[2.2.2]octane-1-carboxamido)ethyl)amino)-3-oxopropyl)carbamoyl)-2-fluorophenyl (*R*,*E*)-1-(Cyclooct-4-en-1-yloxy)-1,12-dioxo-5,8-dioxa-2,11-diazapentadecan-15-oate (**12**)

Following general procedure B, 3-fluoro-4-hydroxybenzamido congener (**10**) (6.67 mg, 9.99 μmol, 1 equiv) was converted to the PEG-TCO conjugate **12**. Purification by RP-HPLC (Method B) gave, after lyophilization, the title compound as an off-white fluffy solid (3.02 mg, 29%). LC-MS *m*/*z* calcd for C_53_H_77_FN_9_O_12_ [MH]^+^: 1050.57, found 1050.80 *t*_R_ = 3.12 min (Method A). ^1^H NMR (400 MHz, CDCl_3_) δ 7.68 (d, *J* = 2.0 Hz, 1H), 7.61 (d, *J* = 8.6 Hz, 1H), 7.54 (t, *J* = 6.0 Hz, 1H), 7.21 (t, *J* = 7.4 Hz, 1H), 6.67 (br s, 1H), 6.52 (br s, 1H), 6.32 (br s, 1H), 5.58 (ddd, *J* = 17.7, 8.5, 4.2 Hz, 1H), 5.48 (ddd, *J* = 15.6, 9.6, 3.3 Hz, 1H), 5.12 (br s, 1H), 4.33 (br s, 1H), 4.09 (t, *J* = 7.3 Hz, 2H), 4.00 (t, *J* = 7.5 Hz, 2H), 3.73 (q, *J* = 5.8 Hz, 2H), 3.61–3.49 (m, 7H), 3.48–3.39 (m, 6H), 3.35–3.27 (m, 2H), 3.04–2.94 (m, 2H), 2.65 (t, *J* = 6.7 Hz, 2H), 2.51 (t, *J* = 5.8 Hz, 2H), 2.38–2.29 (m, 2H), 2.03–1.94 (m, 2H), 1.93–1.81 (m, 8H), 1.77–1.71 (m, 8H), 1.67–1.57 (m, 8H), 1.37 (hd, *J* = 7.4, 2.7 Hz, 4H), 0.95 (dt, *J* = 9.4, 7.3 Hz, 6H). HRMS (TOF ES^+^) calcd for C_53_H_77_FN_9_O_12_ [MH]^+^: 1050.5670, found 1050.5656; calcd for C_53_H_76_FN_9_NaO_12_ [MH]^+^: 1072.5490, found 1072.5491. Analytical RP-HPLC *t*_R_ = 17.24 min, purity >98% (Method C).

### Molecular Modeling Studies

Molecular docking simulation of DITC-XAC, LD probe **11**, and LD probe **12** to the 3.2 Å resolution A_1_AR crystal structure was performed using the Schrodinger software suite (release 2019-2). The 3.2 Å A_1_AR crystal structure in complex with the irreversible ligand DU172 was retrieved from the Protein Data Bank (PDB 5UEN) depository and was first prepared using PyMOL (2.5.4) as follows: one copy of the A_1_AR-dimer crystal structure was removed and the covalent bond between the Y271 and the irreversible antagonist DU172 was broken to facilitate the definition of the docking site during the Grid generation step. This structure was subsequently imported into Maestro and was prepared with the Protein Preparation Wizard tool. Hydrogen atoms were added. The H-bonding network was optimized using PROPKA at pH = 7.0. The structures of the proteins were energy-minimized using the OPLS3 force field. The docking site was defined using Glide Grid generation with the barycenter of the cocrystallized DU172 representing the center of the grid. DITC-XAC, LD probe **11**, and LD probe **12** were prepared for docking using LigPrep tool. Molecular docking of these ligands was performed using Glide with the XP (extra precision) mode and flexible ligand sampling with no restriction applied. For all ligands, the highest docking scoring pose was selected and depicted using PyMOL to include key binding interactions and distance measurements.

## Supporting Information Available

Detailed pharmacology methods, supporting figures, and characterization data of the compounds described (PDF)
Homology (docking) models including ligand bound docking—glide-dock_XP_pDITC-XAC_5UEN (PDB)
glide-dock_XP_probe11_5UEN (PDB)
glide-dock_XP_probe12_5UEN (PDB)
Molecular formula strings of tested compounds (CSV)

## Supplementary Material

jm4c00835_si_001.pdf

jm4c00835_si_002.pdb

jm4c00835_si_003.pdb

jm4c00835_si_004.pdb

## Abbreviations Used

A_2A_R: adenosine A_2A_ receptor
A_1_R: adenosine A_1_ receptor
LDL: ligand-directed labeling
FCS: fluorescence correlation spectroscopy
NL: nanoluciferase
SulfoCy5: sulfonated-cyanine5
PEG-TCO: polyethylene glycol *trans*-cyclooctene
GPCR: G-protein-coupled receptor
NanoBRET: nanoluciferase bioluminescence resonance energy transfer
NanoBiT: nanoLuc binary technology
HRMS: high-resolution mass spectrometry
HPLC: high-performance liquid chromatography
COMU: (1-cyano-2-ethoxy-2-oxoethylidenaminooxy)dimethylamino-morpholino-carbenium hexafluorophosphate
BEP: 2-bromo-1-ethylpyridinium tetrafluoroborate
