# Supplementary material for: Ligand-Directed Labeling of the Adenosine A1 Receptor in Living Cells
Source: J Med Chem. 2024 Jul 12;67(14):12099–117. doi: 10.1021/acs.jmedchem.4c00835 (PMC11284787; doi:10.1021/acs.jmedchem.4c00835)
Supplement: Supplementary file 1 — jm4c00835_si_001.pdf [file jm4c00835_si_001.pdf]

Supporting Information

Ligand-Directed Labelling of the Adenosine A_1 Receptor in Living Cells

*Eleonora Comeo,[1,3,4] Joëlle Goulding,[2,3] Chia-Yang Lin,[1,3] Marleen Groenen,[2,3] Jeanette Woolard,[2,3] Nicholas D. Kindon,[1,3] Clare R. Harwood,[2,3] Simon Platt,[2,3] Stephen J. Briddon,[2,3] Laura E. Kilpatrick,[1,3] Peter J. Scammells,[4] Stephen J. Hill[2,*3] and Barrie Kellam[1,3*]*

[1] Division of Biomolecular Sciences and Medicinal Chemistry, School of Pharmacy, Biodiscovery Institute, University of Nottingham, Nottingham, NG7 2RD, United Kingdom

[2] Division of Physiology, Pharmacology and Neuroscience, School of Life Sciences, Queens Medical Centre, University of Nottingham, Nottingham, NG7 2UH, United Kingdom

[3] Centre of Membrane Proteins and Receptors (COMPARE), University of Birmingham and University of Nottingham, The Midlands, NG7 2UH, United Kingdom

[4] Medicinal Chemistry, Monash Institute of Pharmaceutical Sciences, Monash University, Parkville, Victoria 3052, Australia

AUTHOR INFORMATION

Corresponding Authors

barrie.kellam@nottingham.ac.uk and stephen.hill@nottingham.ac.uk

Table of Contents

General Methods Pharmacology S3

Experiments..S6

Data Analysis S12

Figure S1. Fluorescence spectra of SulfoCy5-conjugated methyltetrazine in the absence or in the presence of probe 12 (PEG-TCO)..S14

Figure S2**.** Assessment of **11** non-specific labelling performed in non-transfected live HEK293T cells...S14

Figure S3. Control experiments of secondary antibody only labelling of Rab 5 early endosomes S15

Figure S4. Fluorescence Correlation Spectroscopy analysis of probe 11 ....S15

Figure S5**.** Live-cell confocal imaging of Dorsal Root Ganglion (DRG) neurons labelled with **11** (250 nM) following A_{2A}-selective antagonist treatment S16

Figure S6 HRMS and Analytical RP-HPLC Chromatogram of probe **11** (SulfoCy5)....17

Figure S7 HRMS and Analytical RP-HPLC Chromatogram of probe **12** (PEG-TCO) S18

NMR SPECTRA S19

REFERENCES..S29

General Methods Pharmacology

Cell culture reagent were purchased from Sigma Chemicals (Pool, Dorset, UK) except Fetal Calf Serum (FCS) which was provided by PAA Laboratories (Teddington, Middlesex, UK). G418 and Optimem were obtained from Life Technologies (Paisley, UK). All plates were obtained from Corning Costar (Corning Incorporated, Corning, NY, USA) unless otherwise stated. FuGENE® transfection reagent and furimazine were purchased from Promega (Southampton, UK). Adenosine receptor ligands, 8-(4-(4-(4-chlorophenyl)piperazide-1-sulfonyl)phenyl)-1-propylxanthine (PSB-603), *N*-[9-chloro-2-(2-furanyl)[1,2,4]-triazolo[1,5-*c*]quinazolin-5-yl]benzene acetamide (MRS1220), 4-(2-[7-Amino-2-(2-furyl)[1,2,4]triazolo[2,3-*a*][1,3,5]triazin-5-ylamino]ethyl)phenol (ZM241385), and *trans*-4-((2-phenyl-7*H*-pyrrolo[2,3-d]pyrimidin-4-yl)amino)cyclohexanol (SLV320) were purchased from Tocris Bioscience (Bristol, UK). 8-Cyclopentyl-1,3-dipropylxanthine (DPCPX), 5'-(*N*-ethylcarboxamido)adenosine (NECA) and all other reagents were purchased from Sigma-Aldrich (UK) and were of analytical quality.

Cell culture. HEK293T cells stably expressing NanoLuc-A_1(human and rat), NanoLuc-A_3 and NanoLuc-A_{2B} were generated as described previously.[2–4] HiBiT-A_1 stable cell line was generated as reported in Soave, M. et al.[5] DNA encoding the TwinStrep tag[6] in frame with a SNAP tag, separated by a glycine-serine-serine-glycine linker (with the SNAP start codon mutated to leucine), was purchased from Twist Bioscience (San Francisco, CA, USA). The Twin-Strep-SNAP DNA was ligated in frame into a pcDNA3.1+ Neo A_1 plasmid, following restriction with enzymes KpnI and BamHI (Promega, Wisconsin, USA)[2], resulting in an expression construct of TwinStrep-SNAP-A_1, preceded by the signal peptide of the murine 5HT3A receptor (pcDNA3.1+ Neo TwinStrep-SNAP-A_1).

Human embryonic kidney 293 (HEK293) cells expressing the GloSensor cAMP biosensor (HEKG) were obtained from Promega (Southampton, UK). The HEK293 cell lines were maintained in DMEM containing 10% FCS and 4 mM L-glutamine and were grown to 70−80% confluency in 75 cm^2 tissue culture flask before splitting. All cell colonies were incubated at 37 °C, 5% CO_2, and the tissue culture procedures were performed in a class II laminar flow hood using sterile conditions. *Transient Transfection of SNAP-tagged hA_1AR in HEK293T cells.* Cells were seeded in 8-well

plates (Nunc Lab-Tek, Thermo Fischer Scientific), pre-coated with Poly-D-Lysine, at 20,000 cells per well in DMEM/10% FCS. 24 h post seeding, cells were transiently transfected with human SNAP-A_1. Transient transfections were carried out following manufacturer's protocol: used FuGENE (Promega) HD at 3:1 ratio of reagent to cDNA with a total of 300 ng cDNA per well. Transfection solutions were made up in Gibco Opti-MEM (Thermo Fischer Scientific), incubated at RT for 5 min and added as 11 µL per well. Cells were incubated for additional 24 h at 37 ^{O}C, 5% CO_2. *Transient Transfection* of NanoLuc-tagged human A_{2A}AR in HEK293T cell line was carried out as follows: 72 h prior to the experiment cells were removed from a confluent T75 flask and centrifuged. The pellet of cells was resuspended in 10 mL of fresh complete DMEM. Afterwards, cells were seeded in a 6-well plate at a density of $5x10^5$ cells/well (final volume/well is 2 mL).Then 48 h prior the experiment transfection of the NanoLuc-tagged human A_{2A}AR in HEK 293T cell line was carried out using a mixture of 0.25 µg DNA and 0.75 µL of FuGENE (1:3 ratio) in 98.6 µL of OptiMEM (per well), followed by 10 min incubation at room temperature to allow the DNA:FuGENE complex to form. After that time, the entire mixture (100 µL/well) was carefully added to the cells, mixed thoroughly by rocking the plate and cells were incubated at 37 °C, 5%CO_2 overnight. 24 h post transfection, media was removed and cells were carefully washed with PBS, followed by the addition of trypsin (500 µL/well). Cells were centrifuged and seeded in white flat bottomed 96-well Greiner plates (Bio One, UK) precoated with Poly-D-Lysine.

Adult Rat Dorsal Root Ganglion (DRG) Preparation

Dorsal Root Ganglion (DRG) neurons were dissected from adult (10-12 weeks) male Sprague-Dawley rats (Charles River Laboratories, UK). Animals were housed and cared as reported in Cooper, S. et al.[7] according to the requirements of the UK Animals (Scientific Procedures) Act 1986 (ASPA) draft Code of Practise for the care and accommodation of animals (February 2013). The procedures of dissecting tissues from rats (non-licensed study pro-forma involving animals) were approved by the University of Nottingham Animal Welfare and Ethical Review Board (AWERB). Dissection of the spinal column and DRGs was adapted from reported procedures.[8,9] Briefly, DRGs were placed in PBS and washed once by gravity. A solution of collagenase (5 mL) was added to the cells, which were then incubated for 90 min at 37 ^{O}C/ 5% CO_2. The DRGs were

gently washed in PBS (3x) by gravity. Then 2 mL of a solution of trypsin was added to the DRGs, which were then incubated for 15 min at 37 ^{O}C/ 5% CO_2. The DRGs were allowed to settle so that 1.5 mL of the trypsin solution could be removed from the DRGs. Then, 1 mL of the 16% BSA solution was added to the DRGs and triturated to totally dissociate the DRGs (8-12 passes up and down with a 1 mL tip). Taking care to not produce excessive amounts of bubbles the pipette tip was placed below the fluid surface. The cell suspension was carefully layered on top of 7 mL of the BSA solution in a 30 mL universal. Cells were centrifuged at 1200 rpm for 10 min. The top layer was carefully removed as much as possible with a pipette. Then 700 μL of complete Primary Neuron Basal Medium (PNBM), supplemented with InSolution^{TM} Aphidicolin (4 μM) and Penicillin-Streptomycin (200 I.U./mL), was added to the cell pellet, which was gently resuspended. To each well of an 8-well chamber plate were added 20 μL of cell suspension. Then the plates were incubated for 20-25 min at 37 ^{O}C/ 5% CO_2. Lastly, 300 μL of complete PNBM media were added per well and plates were incubated for 24/48 h prior imaging. *Coating of 8-well plates*: 300 μL of a solution of Poly-D-Lysine and Laminin was added to each well. This was prepared by adding 40 μL of Poly-D-Lysine at 5 mg/mL in 20 mL PBS (0.22 μm filtered sterilized) and 10 μL laminin at 1 mg/mL. Following coating, the plates were incubated 24 h at 4 ^{O}C, then washed with PBS and dried in the TC hood prior to DRGs seeding. *DRGs culture reagents* were prepared as follows. Complete growth Primary Neuron Basal Medium (PNBM) (Lonza Biosciences, UK) was prepared by the addition of Primary Neuron Growth Medium (PNGM) SingleQuots supplements and growth factors (cat # CC-4462): CC-4459HH-NSF-1 (4 mL), CC-4460HH L-glutamine (2 mL) and PT-4505HH GA (0.2 mL). The collagenase solution was prepared with 5 mL of complete PNBM and 12.5 mg of collagenase (Sigma cat # C5138). The trypsin solution from porcine pancreas was from Sigma (cat# T4549). Laminin (1 mg/mL) was from Sigma (cat # L2020). BSA solution (16 % w/v) was prepared by dissolving 8 g of Bovine Albumen in 40 mL HBSS-HEPES to final pH = 7.4. HBSS-HEPES (pH = 7.4) solution was prepared by mixing 50 mL of 10x HBSS (Sigma cat # H1641) and 1.21 g HEPES (Sigma cat # H9136) and double distilled H_2O.

Experiments

NanoBRET binding assay. All NanoBRET experiments were performed in white flat bottomed 96-well Greiner plates (Bio One, UK) precoated with Poly-D-Lysine and were read on a PheraSTAR FS plate reader (BMG Labtech) at room temperature or at 37 ^{o}C for kinetic binding experiments. Emissions were read at 420 nm (80 nm bandpass; donor NanoLuc emission) and > 610 nm (longapass; fluorescent probe emission) for the SulfoCy5-labelled probe. The raw BRET ratio was calculated by dividing the > 610 nm emission by the 420 nm emission. The term "raw BRET ratio" refers to the unprocessed data as no background ratio has been subtracted. In all NanoBRET binding experiments, non-specific binding was determined in the presence of 10 μM competitive antagonist selective for the adenosine receptor under investigation (DPCPX for the A_1AR, ZM214385 for the A_{2A}AR, PSB603 for the A_{2B}AR and MRS1220 for the A_3AR). HEK293T cells stably/transiently expressing Nluc-AR were seeded in white 96-well plates Greiner plates (Bio One, UK) at 32000 cells/well and grown 24 h prior the day of the experiment in normal growth medium. Before the experiment, DMEM was replaced with warm HEPES buffered saline solution (HBSS: 145 mmol/L NaCl, 5 mmol/L KCl, 1.7 mmol/L $CaCl_2$, 1 mmol/L $MgSO_4$, 10 mmol/L HEPES, 2 mmol/L sodium pyruvate, 1.5 mmol/L $NaHCO_3$, 10 mmol/L D-glucose, pH 7.4). For saturation and competition assays the required concentration of ligand-directed probe and competing ligand was added at the same time. Thereafter, plates were incubated for 1 h at 37 °C. After 1 h, 10 μM furimazine (1:400 dilution; Promega) was added to each well and plates were incubated for further 5 min at 37 °C. For dissociation kinetic experiments, stable human and rat NanoLuc-A_1AR HEK293T cells were incubated with compounds for 5 h or 2 h for CA200645 at 37 °C in 96-well plates. After that time, 10 μM furimazine was added to each well and incubated at 37 ^{o}C for 15 min in the dark to allow the luminescence signal to reach equilibrium. Basal BRET readings were acquired on the PheraSTAR every 30 s for 5 min before 10 μM DPCPX was added manually in a 1:10 ratio and BRET readings were taken every 30 s for 60 min at 37°C.

NanoBiT complementation assay. HEK293T cells stably expressing HiBiT–A_1AR were seeded onto white 96-well plates (Greiner Bio-One, Monroe, NC), pre-coated with Poly-D-Lysine (10 μg/mL), at 30,000 cells per well in DMEM/10% FCS and the plate incubated at 37 °C in 5% CO_2 . 24 h post seeding, the medium was removed from each

well and replaced with 50 μL Serum-free DMEM (SFM) with or without 1 μM **11** (SulfoCy5) and cells were incubated for 1 h at 37 °C in a 5% CO_2. Thereafter, SFM was removed and cells were washed twice with warm HBSS. The A_1AR-selective antagonist DPCPX was added to the appropriate well at 10 nM concentration and the plates were incubated for 10 min at 37 °C. After 10 min, NECA was added at increasing concentrations and the plates were incubated for further 2 h at 37 °C. Following 2 h incubation, purified LgBiT was diluted in HBSS and added to each well (final concentration 10 nM). Cells were incubated 20 min at 37 °C, allowing complementation to occur and furimazine was added to each well (1:400 final concentration, 10 μM). The plate was incubated for 15 min in the dark at 37 °C. Luminescence was measured on the PHERAstar FS plate reader (BMG Labtech, Offenburg, Germany) using the LUM Plus module.

A_1AR internalisation and Rab 5 Immunolabelling. On the day of the experiment, transiently transfected SNAP-tagged hA_1AR HEK293T cells were washed with warm SFM and labelled with 30 nM ligand-directed probe **11** (SulfoCy5) in SFM for 2 h at 37 °C 5% CO_2. Media was removed and cells were washed 3x5 min with warm SFM. Vehicle or a solution of A_1AR-selective agonist CCPA (10 μM) was added to the required wells to stimulate an internalisation response and cells were incubated for 2 h at 37 °C, 5% CO_2. Thereafter, cells were washed once with PBS and were fixed with 3% paraformaldehyde (PFA) in PBS for 20 min at room temperature. Cells were washed 3x 5 min with PBS (200 μL/well) and permeabilised with 0.025% Triton-X-100 for 10 min at room temperature, followed by 3x 5 min wash with PBS. Then, to minimize cross-linking and non-specific antibody binding, cells were incubated with 3% BSA/1% glycine in PBS for 30 min at room temperature and then washed 3x5 min with PBS. Next, cells were blocked by the addition of 10% chicken serum in PBS for 30 min at room temperature. Cells were washed 3x 5 min with PBS and labelled with the primary rabbit Anti-Rab5 monoclonal antibody (Cell Signalling Technology, Leiden, The Netherlands) diluted in 10 % secondary antibody serum (1:100 dilution) at 4 °C overnight. The following day, cells were washed with 5x 5 min PBS and incubated with the AF488-labelled secondary antibody anti Rabbit IgG (1:500 dilution) diluted in 10% chicken serum in PBS for 1 h in the dark at room temperature. Cells were washed 3x 5 min with PBS. PBS was added in each well (200 μL/well) and cells were imaged

using a Zeiss LSM880 confocal microscope as described in the confocal imaging section.

Confocal Imaging Studies

HEK293T cells were seeded in 8-well plates (Nunc Lab-Tek, Thermo Fischer Scientific), pre-coated with Poly-D-Lysine, at 20,000 cells/well in DMEM/10% FCS. 24 h post seeding, cells were transiently transfected with human SNAP-A_1. Transient transfections were carried out following manufacturer's protocol: used FuGENE HD at 3:1 ratio of reagent to cDNA with a total of 300 ng cDNA per well. Transfection solutions were made up in Gibco Opti-MEM (Thermo Fischer Scientific), incubated at RT for 5 min and added as 11 µL per well. Cells were incubated for additional 24 h at 37 ^{O}C, 5% CO_2. On the day of the experiment medium was removed and the cells were labelled for 30 min at 37 °C with 0.2 µM SNAP-surface AF488 in SFM. After washing twice with SFM the cells were treated with or without 10 µM competitive A_1AR-antagonist DPCPX for 30 min at 37 °C, 5% CO_2. Following the incubation step, the ligand-directed probe under investigation (SulfoCy5 or PEG-TCO) (**11** or **12**) was added to the appropriate wells at the required concentrations and incubated for 2 h. After that time, cells were washed 4x with warm HBSS, followed by the addition of 300 µL warm HBSS and imaged. For click-chemistry reactions, after washing 4x with warm HBSS, cells pre-labelled with the *trans*-cyclooctene-conjugated **12** (PEG-TCO) at 50 nM were subsequently treated with 1 µM Methyl-TetrazineSulfoCy5 (Lumiprobe, Germany) for 5 min at RT in the dark. Cells were washed 2x with warm HBSS and imaged. When required, 10 µM DPCPX was added after 2 h and cells were incubated for and additional 1 h before imaging. DRG neurons were seeded in 8-well plates pre-coated with a solution of Poly-D-Lysine and Laminin and were incubated in complete growth PNBM media for 48 h at 37 ^{O}C, 5% CO_2. On the day of the experiment, media was removed and the DRGs were treated with or without a solution of 10 µM DPCPX in serum free PNBM for 30 min. Thereafter, DRGs were labelled with 250 nM **11** (SulfoCy5) and incubated for 2 h at 37 ^{O}C, 5% CO_2. Cells were washed 1x with HBSS and imaged.

Live cell imaging was performed using either a Zeiss LSM 710 or LSM880 laser scanning confocal microscope fitted with a Zeiss C-Apochromat 40 × 1.2 NA water immersion objective. A 633 nm HeNe laser was used for the excitation of SulfoCy5 fluorophore and a 488/561/633 dichroic, and the emission was detected between 634

and 755 nm. A 488 nm HeNe laser was used to excite SNAP-AF488 and the emission was detected between 493 and 586 nm. The pinhole diameter (1 Airy Unit; 1.1 μm optical slice), laser power and gain was kept constant in all the experiments. Images were acquired as 16-bit depth, 1024x1024 pixel resolution with a line averaging of 8 (or 2) and a pixel dwell time 4.12 μs (set to 5). Images were either processed in Zeiss ZEN 2 (blue edition) or FIJI (ImageJ) version 2.0.0 software, and linear adjustments to the brightness and contrast have been applied equally across all the images. To obtain membrane intensity values, regions of interest were drawn by hand around regions of cell membranes in FIJI (ImageJ)[10] version 2.0.0 software.

Fluorescence Correlation Spectroscopy. HEK293T cells were seeded in 8-well plates (No. 1 borosilicate glass bottomed Nunc Lab-Tek, Thermo Fisher Scientific), pre-coated with Poly-D-Lysine, at 10,000 cells/well in DMEM/10% FBS. 24 h post seeding, cells were transiently transfected with human SNAP-A_1. Transient transfections were carried out following manufacturer's protocol: used FuGENE HD at 3:1 ratio of reagent to cDNA with a total of 300 ng cDNA per well. Transfection solutions were made up in Gibco Opti-MEM (Thermo Fisher Scientific), incubated at rt for 5 min and added as 11 μL per well. Cells were incubated for additional 24 h at 37 ^{O}C, 5% CO_2. On the day of the experiment medium was removed and the cells were labelled for 30 min at 37 °C with 0.1 μM SNAP-surface AF488 in SFM. After washing with SFM the cells were treated with or without 10 μM competitive A_1AR-antagonist DPCPX for 30 min at 37 °C, 5% CO_2. Following the incubation step, probe **11** was added to the appropriate wells at 100 nM and incubated for 90 min. After that time, cells were washed 3x with warm HBSS over the course of 20 min, followed by the addition of 300 μL HBSS, left to cool to 24 ^{O}C and used in Fluorescence Correlation Spectroscopy (FCS) studies.

FCS was performed on a Zeiss LSM880 microscope on a Zeiss Axio Observer Z1 stand fitted with 40X c-Aprochromat 1.2 NA water immersion objective as previously described.[11] The beam path utilised a HeNe 633 nm laser excitation, a 488/561/633 multi beam splitter, with emission collected through a 639-691 nm filter and the pinhole set to 1 Airy Unit. For solution-based reads 10 x 10s trace measurements were taken within 200 μL sample with the confocal observation volume placed 200 μm above the coverslip. For apical membrane reads, 3 x 30s trace measurements at ~0.5 kW/cm^2 laser power were taken on the apical membrane with its position determined as the peak of a point intensity

Z-scan utilising an argon 488 nm laser excitation, at ~0.5kW/cm^2 laser power, detecting the SNAP-surface AF488-labelled Adenosine A_1AR. The placement was lowered by 0.2 µm to account for the optical chromatic offset between the 488 nm and 633 nm detection previously determined for our microscope set-up.

Autocorrelation curves (AC) were constructed from fluorescence fluctuation traces and analysed within Zen Black (2012) software. Solution based reads employed a 1x3D diffusion model and membrane reads used a diffusion model that combined a 3D component confined to 20-200 µs to account for free probe and a 2D component to account for diffusion of a membrane protein.[11] The diffusion model also describes a pre-exponential factor to account for fluorophore photophysics, an offset factor to allow the asymptote to plateau >1 and the structural parameter fixed at 5.[11] The radial and vertical axes of the confocal observation volume were determined on each experimental day by measuring the diffusion of 10 nM Cy5, as described previously[12] where the diffusion coefficient (D) of Cy5 was taken as 3.15 × 10–6 cm^{2}s-1.

Fluorescence fluctuation traces were reanalysed with Photon Counting Histogram analysis to determine the average molecular brightness of detected species. A time bin of 100 ms (for membrane traces) or 20 µs (for solution traces) was applied to all traces, with the system-dependent first order correction, to account for photons detected outside of a true Gaussian detection volume, determined on each experimental date from the Cy5 calibration data.

Labelling of TS-SNAP-A_1 with compound 11 and compound 12 for purification and in gel fluorescence. For SDS-PAGE and in gel fluorescence assays HEKG-TS-SNAP-A_1 cells were grown in T175 tissue culture flasks and 1 flask was used per purification sample. Growth media was aspirated and replaced with 10mL labelling solution. Labelling solution consisted of 300nM compound 11 or 130 nM compound 12 in serum free media. Labelling took place for 2 hrs at 37 ^{o}C and 5% CO_2. Labelling solution was removed from the compound 12 flask and immediately replaced with 10mL of serum free media containing 1µM Met-Tet-SulfoCy5 and incubated at 37°C and 5% CO_2 for a further 15 mins. All cells were then wash x2 with 10mL Phosphate Buffered Saline (PBS), before cells were detached from flasks using cell dissociation solution non-enzymatic (sigma). Cells were washed off with PBS. Cells were pelleted at 200*xg* for 3 mins. Supernatant was aspirated and cell pellets frozen at -80 ^{o}C until use.

Extraction and purification of compound 11 and compound 12 labelled TS-SNAP-A_1. Cell pellets were thawed on ice, weighed and resuspended in solubilisation buffer (0.5% (w/v) Lauryl Maltose Neopentyl Glycol (LMNG) (ThermoScientific, U.K), 0.01% (w/v) Cholesteryl Hemisuccinate Tris salt (Anatrace, O.H, U.S.A), 20mM HEPES, 10% (v/v) glycerol, 150mM NaCl, complete protease inhibitors (Roche, U.K), pH7.5) at a ratio of 1:10 (w/v) of cell pellet to solubilisation buffer. Pellets were solubilised for 1hr on DigiRoller 6 roller (SLS, U.K) at 80RPM and 4°C. Samples were clarified by centrifugation at 16,000*xg* for 20min at RT. Purification of TS-SNAP-A_1 was achieved by the use of MagStrep "type3" XT magnetic beads (IBA, Göttingen, Germany). Beads were prepared by removal of supernatant using a magnetic separator (IBA, Göttingen, Germany) and then they were washed twice in solubilisation buffer before being added to samples. Samples were incubated with beads overnight on head-to-head shaker. The following morning supernatant was removed from beads using the magnetic separator and beads were washed twice with solubilisation buffer, before resuspension in 30 μL elution buffer (1:9 solution of 10x buffer BXT (IBA) and solubilisation buffer). Elution took place for 3 h on a DigiRoll 6 roller set to 80 RPM at 4 °C. Samples were then separated from beads using magnetic separator and then immediately processed for electrophoresis.

SDS-PAGE gel electrophoresis and in gel fluorescence. Fifteen microlitre of samples containing purified TS-SNAP-A_1 were mixed with 5 μL NuPAGE™ LDS sample buffer and resolved on a NuPage™ 4–12% Bis-Tris 15 × 1.0 mm well gel using NuPage™ MES SDS running buffer. Gels were run for 50 min at 200 V. Samples were not boiled prior to gel electrophoresis. 5 μL PageRuler™ Prestained Protein Ladder was used as the ladder. Gels were scanned on an Amersham Typhoon imaging system (GE Healthcare Life Sciences, Pittsburgh, PA) using Fluorstage and Cy5 670BP30 filter sets with PMT set to auto and pixel size to 200 μm.

Data Analysis

All data are represented as mean ± SEM of *n* experiments performed in triplicate. The *n* refers to the number of separate experiments. A separate experiment required a separate flask of cells and a separate drug dilution used throughout the experiment. The data were presented and analysed using Prism software (GraphPad Prism 9.0 and 9.5, San Diego, CA) and Excel.

NanoBiT internalisation assay Antagonism by DPCPX of the HiBiT-A_1AR internalisation response to NECA in HEK293 cells expressing the human A_1AR was analysed. For the single concentration of the antagonist, the ratio (DR, dose ratio) of NECA concentrations required to produce the same sized response in the presence and absence of the antagonist was determined. The antagonist dissociation constant (K_B), was determined by fitting the data to the Gaddum equation:

$$\text{Log (DR-1)} = \text{Log([B])} - \text{Log}K_B$$

Where DR (dose ratio) is the ratio of the agonist concentration required to stimulate an identical response in the presence or absence of antagonist, [B]. K_B represents the equilibrium dissociation constant of the antagonist.

NanoBRET saturation binding assay. Total and nonspecific saturation binding curves were fit simultaneously using the following equation:

$$BRET\ ratio = \frac{B_{max}[B]}{B + (K_D)} + M(B) + C$$

Where B_{max} is the maximal specific binding, [B] is the concentration of fluorescent ligand in nM, K_D is the equilibrium dissociation constant in nM, M is the slope of the nonspecific binding component, C is the intercept with the y axis.

NanoBRET competition binding assay. The competition binding curves were fit to the following equation:

$$Uninhibited\ specific\ binding = 100 - \frac{100\ x\ [A]}{[A] + (IC_{50})}$$

Where [A] is the concentration of competing drug and the IC_{50} is the molar concentration of ligand required to inhibit 50% of the specific binding of specified concentration (25 nM) of fluorescent ligand CA200625.

The Cheng-Prusoff equation was used to correct fitted IC_{50} values to K_i values:

$$K_i = \frac{IC_{50}}{1+\frac{L}{K_D}}$$

Where L is the concentration of fluorescent ligand in nM and K_D is the dissociation constant of fluorescent ligand in nM (18.5 nM). The K_D values used were calculated from the saturation binding experiments.

Supplementary Figures: Pharmacology

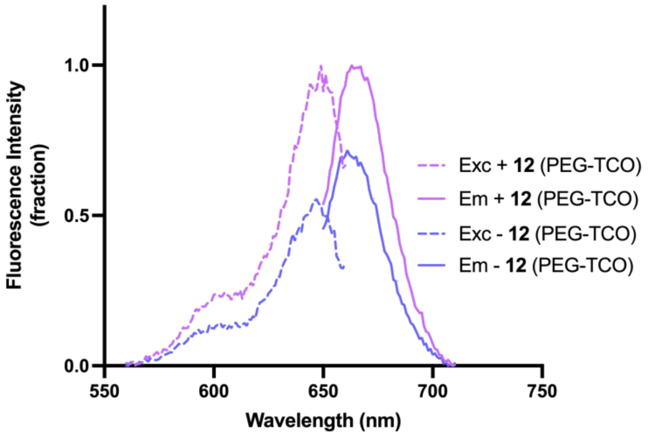

Figure S1. Fluorescence spectra of SulfoCy5-conjugated methyltetrazine in the absence or in the presence of probe 12 (PEG-TCO). "Turn-on" type reaction of tetrazine (Met-TetSulfoCy5) with PEGylated *trans*-cyclooctene (PEG-TCO)-labelled **12**. Fluorescence excitation and emission spectra measured in HBSS with the corresponding tetrazineSulfoCy5 [100 nM] without, then with **12** (PEG-TCO) [1 µM] (allowed to react for 10 min prior detection). Exc maxima (Met-TetSulfoCy5 + PEG-TCO) = 1.80-fold increase compared to Met-TetSulfoCy5 alone; Em maxima (Met-TetSulfoCy5 + TCO) = 1.40-fold; Excitation and emission fluorescence spectra (1 nm step width) were measured were recorded on a CLARIOstar plate reader (BMG Labtech).

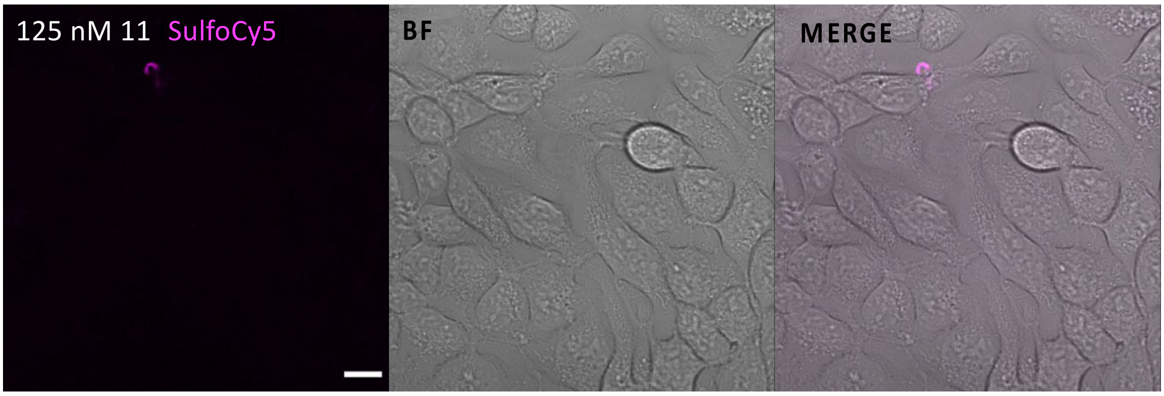

Figure S2. Assessment of **11** non-specific labelling performed in non-transfected live HEK293T cells. Left hand frame corresponds to SulfoCy5 channel. Middle frame is Bright field. Right hand frame is merge image of the two channels. Images are representative of images acquired from $n = 2$ independent experiments. Scale bar = 10 µm

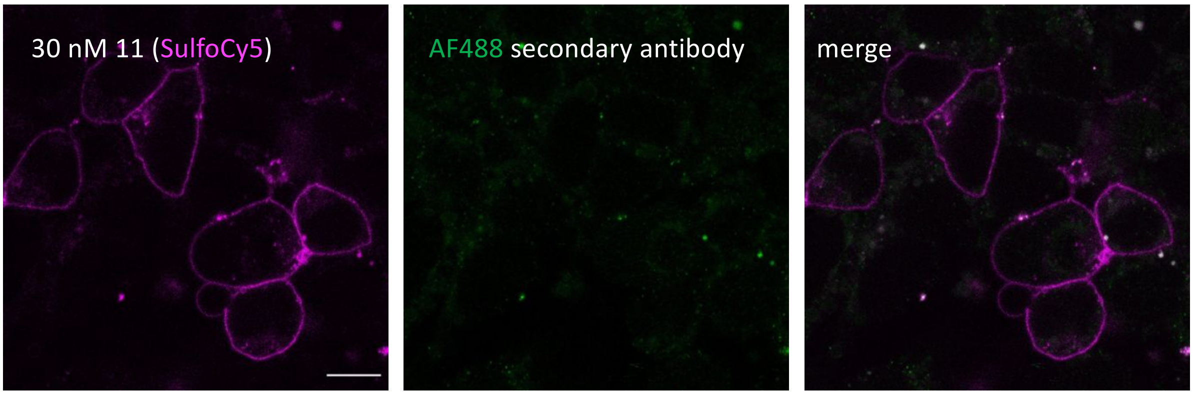

Figure S3. Control experiments of secondary antibody only labelling of Rab 5 early endosomes. Following the labelling by probe **11**, SulfoCy5-bioconjugated SNAP-A_1AR HEK293 cells treated with vehicle (HBSS, under unstimulated conditions). Cells were fixed using 3% PFA/PBS, permeabilised using Triton-X-100 (0.025% in PBS). Cells were then incubated with a secondary antibody (anti rabbit AlexaFluor 488) in the absence of the primary antibody specific for Rab 5 endosomal compartments (rabbit anti Rab 5 monoclonal antibody). The merge image of the SulfoCy5 channel (lefthand frame) and AF488 channel labelling is shown in the right-hand frame Images are Data are representative of 3 individual experiments and were performed in parallel with those in **Figure 7.** Scale bar = 10 µm

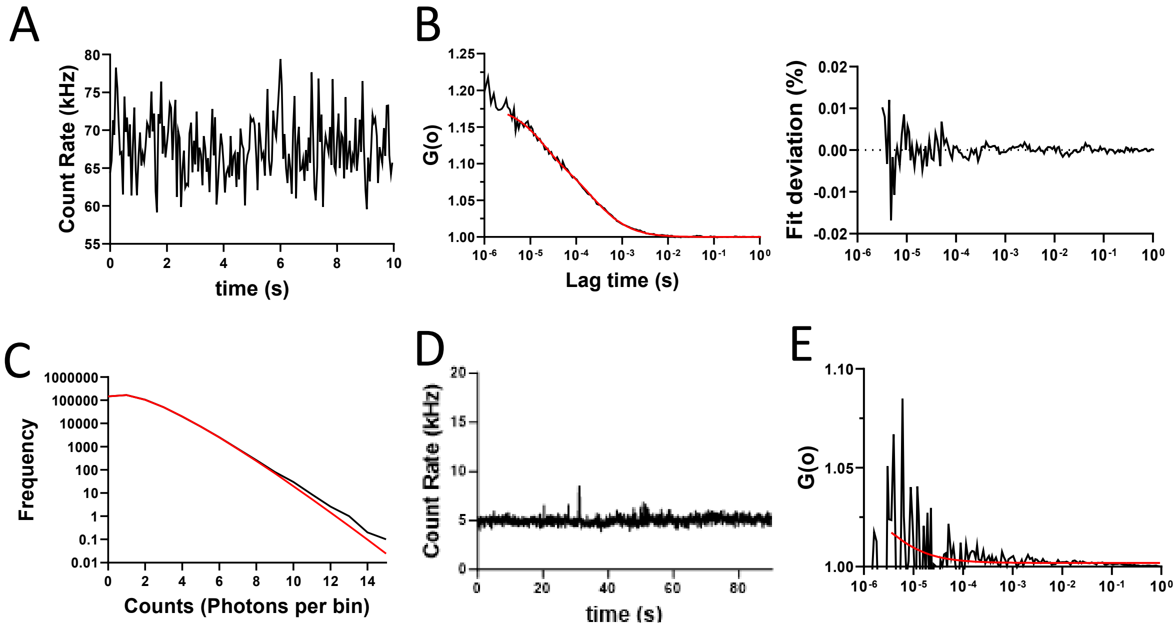

Figure S4. Fluorescence Correlation Spectroscopy analysis of probe 11; (A) Fluorescence fluctuation trace recorded from free diffusion of probe **11** (100 nM) in HBSS (solution-based FCS). (B) Constructed autocorrelation curve from (A), fit with a 1x3D diffusion model (red

line), with deviation from fit displayed next. (C) Photon Counting Histogram analysis of 10x 10 s probe **11** trace reads in HBSS. Data are fit with a 1-component PCH model (red line). (D) Fluorescence fluctuation trace recorded on the apical membrane of SNAP-A_1AR HEK293 cells treated with **11** after pre-incubation with DPCPX (10 μM). (E) Constructed autocorrelation curve from (D), fit with a 1x3D 1x2D diffusion model (red line). Signal is too low resulting in a poor autocorrelation curve and a nonsensical fit.

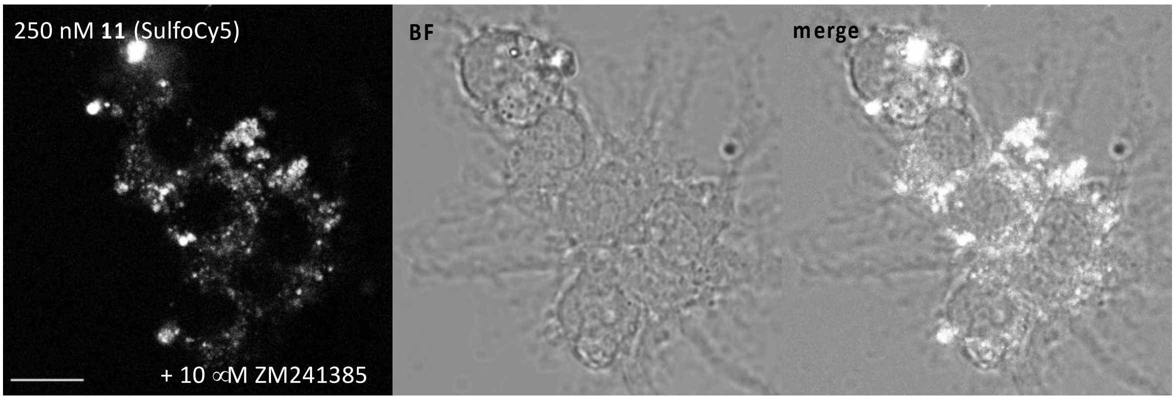

Figure S5. Live-cell confocal imaging of Dorsal Root Ganglion (DRG) neurons labelled **11** (250 nM) following A_{2A}-selective antagonist treatment. Cells were labelled in the presence of 10 μM ZM241385 (A_{2A}AR-selective antagonist). Left-hand frame represents the SulfoCy5 channel, middle frame represents brightfield and right-hand frame represents merged images from both channels. Images are representative of images acquired in four independent experiments. Scale bar = 10 μm.

Supplementary Figures: Chemistry

Sample-ID	e_com_EC-229-2-063-1_SulfoCy5-2	Lab	C13
Submitter	Eleonora Comeo	Supervisor	Barrie Kellam
Analysis Name	e_com_EC-229-2-063-1_SulfoCy5-2_20_01_98043.d	Acquisition Date	15/03/2021 14:48:48
Ionisation Mode	ESI Negative	Instrument	Bruker MicroTOF

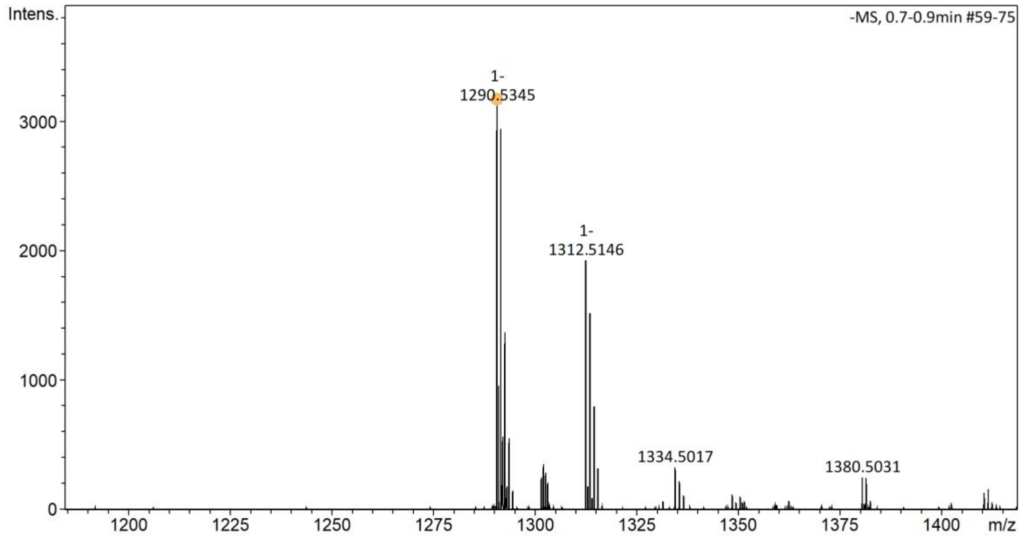

Meas. m/z	#	Ion Formula	Score	m/z	err [mDa]	err [ppm]	mSigma
1290.534484	1	C66H81FN9O13S2	100.00	1290.538477	4.0	3.1	68.5

Note: Sigma fits < 0.05 indicates high probability of correct MF

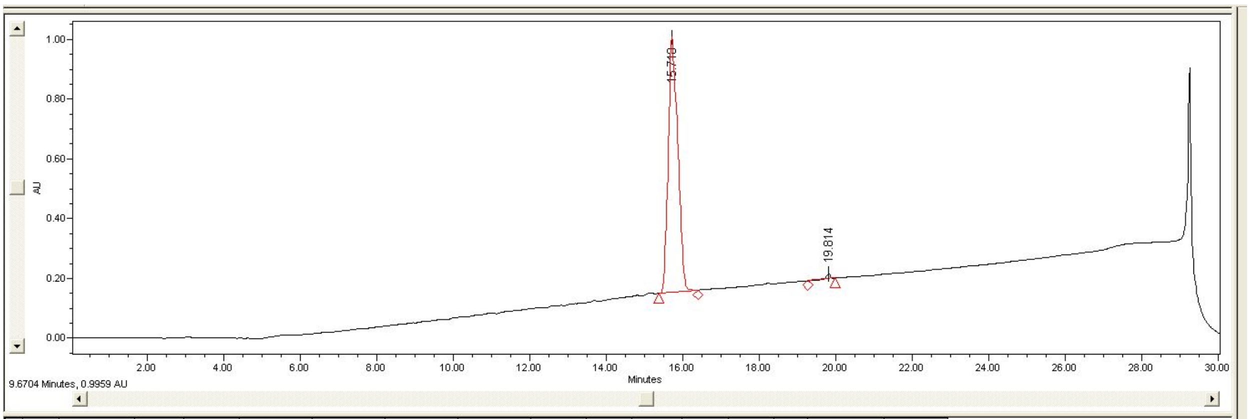

	Name	Retention Time (min)	Purity1 Angle	Purity1 Threshold	PDA Match1 Spect. Name	PDA Match1 Angle	PDA Match1 Threshold	PDA Match1 Lib. Name	Area (µV*sec)	% Area	Height (µV)	Int Type	Amount	Units	Peak Type	Peak Codes
1		15.718							14745129	98.92	855106	bv			Unknown	
2		19.814							160742	1.08	16836	vb			Unknown	

3D Channels 2D Channels Peaks

Figure S6 HRMS (top) and Analytical RP-HPLC Chromatogram (bottom) of probe **11** (SulfoCy5)

Sample-ID	e_com_EC-229-2-081-1_TCOpeg	Lab	C13
Submitter	Eleonora Comeo	Supervisor	Barrie Kellam
Analysis Name	e_com_EC-229-2-081-1_TCOpeg_616_23_01_102316.d	Acquisition Date	6/7/2021 4:19:05 PM
Ionisation Mode	ESI – Negative	Instrument	Bruker MicroTOF

-MS, 0.7-0.9min #59-75

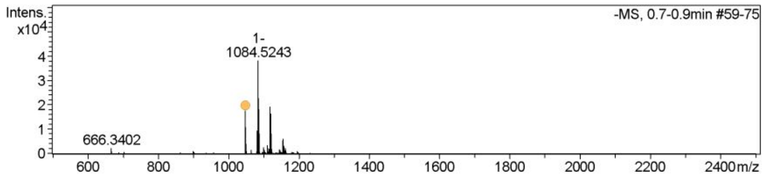

#	m/z	I %
1	1048.5494	46.4
2	1049.5514	28.9
3	1050.5549	10.4
4	1082.5094	25.0
5	1083.5146	16.0
6	1084.5243	100.0
7	1085.5292	60.0
8	1086.5250	47.7
9	1087.5280	21.9
10	1111.5460	9.0
11	1118.4864	50.8
12	1119.4906	31.1
13	1120.4853	43.4
14	1121.4874	21.8
15	1122.4871	12.6
16	1154.4642	14.6
17	1155.4664	9.5
18	1156.4616	16.3
19	1157.4642	8.8
20	1158.4614	7.9

Generate Molecular Formula Parameters

Charge	Tolerance	sigma limit	H/C Ratio	Electron Conf.	Nitrogen Rule	Chrom.BackGround	Calibration
-1	6 ppm	0.08	3 - 0	both	false	false	TRUE

Expected Formula C53 H76 F1 N9 O12 **Adduct(s):** H, radical

#	meas. m/z	theo. m/z	|Err|[ppm]	Sigma	Formula	Adduct	Adduct Mass
1	1 048.5494	1 048.5525	2.90	0.0058	C53H75FN9O12	M- H	1.0078

Note: Sigma fits < 0.05 indicates high probability of correct MF

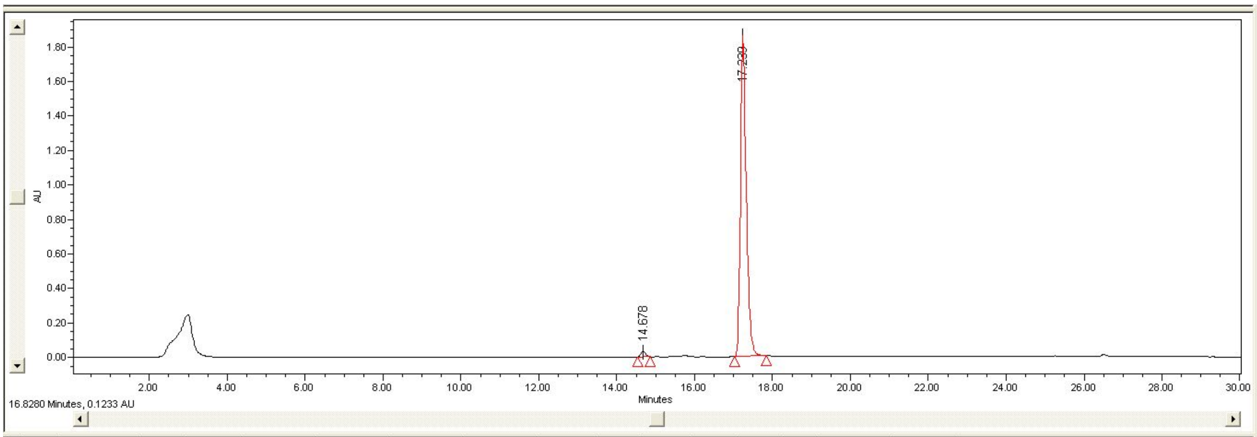

	Name	Retention Time (min)	Purity1 Angle	Purity1 Threshold	PDA Match1 Spect. Name	PDA Match1 Angle	PDA Match1 Threshold	PDA Match1 Lib. Name	Area (µV*sec)	% Area	Height (µV)	Int Type	Amount	Units	Peak Type	Peak Codes
1		14.678							249821	1.27	30040	bb			Unknown	
2		17.239							19494728	98.73	1855591	bb			Unknown	

Figure S7 HRMS (top) and Analytical RP-HPLC Chromatogram (bottom) of probe **12** (PEG-TCO)

NMR SPECTRA

^{1}H NMR (400 MHz, DMSO-d_6) of **1**

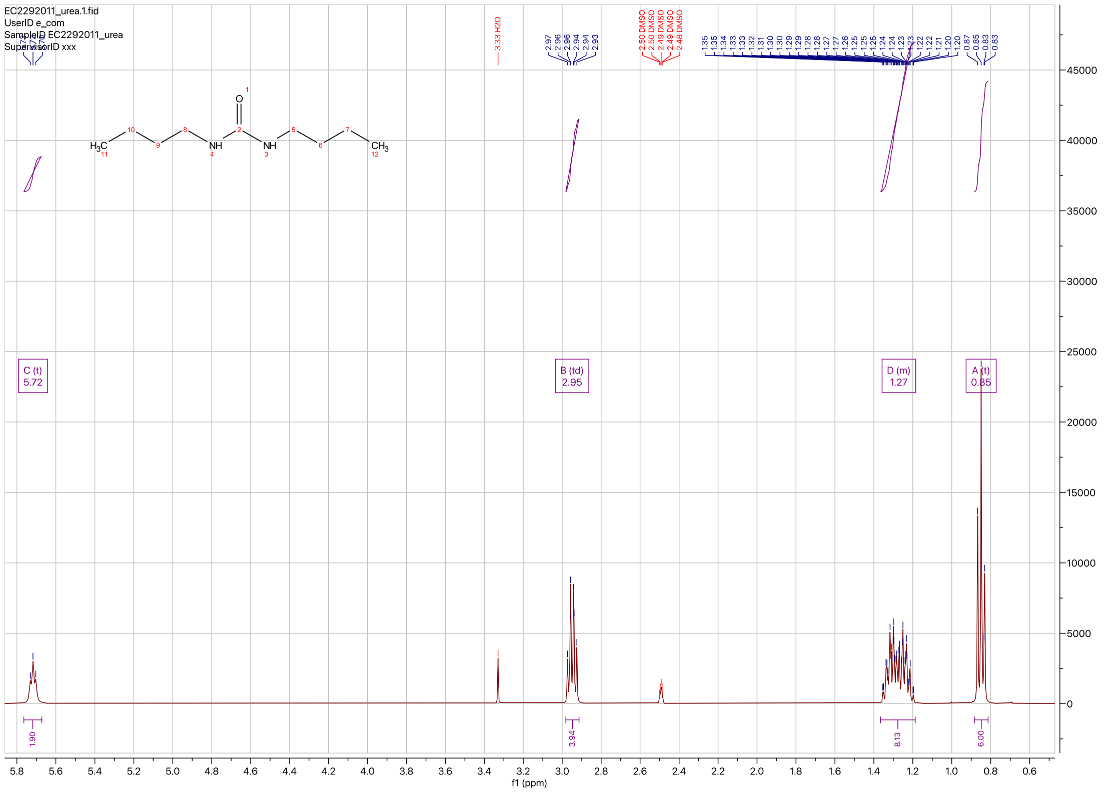

^{13}CNMR (101.62 MHz, DMSO-d_6) of **1**

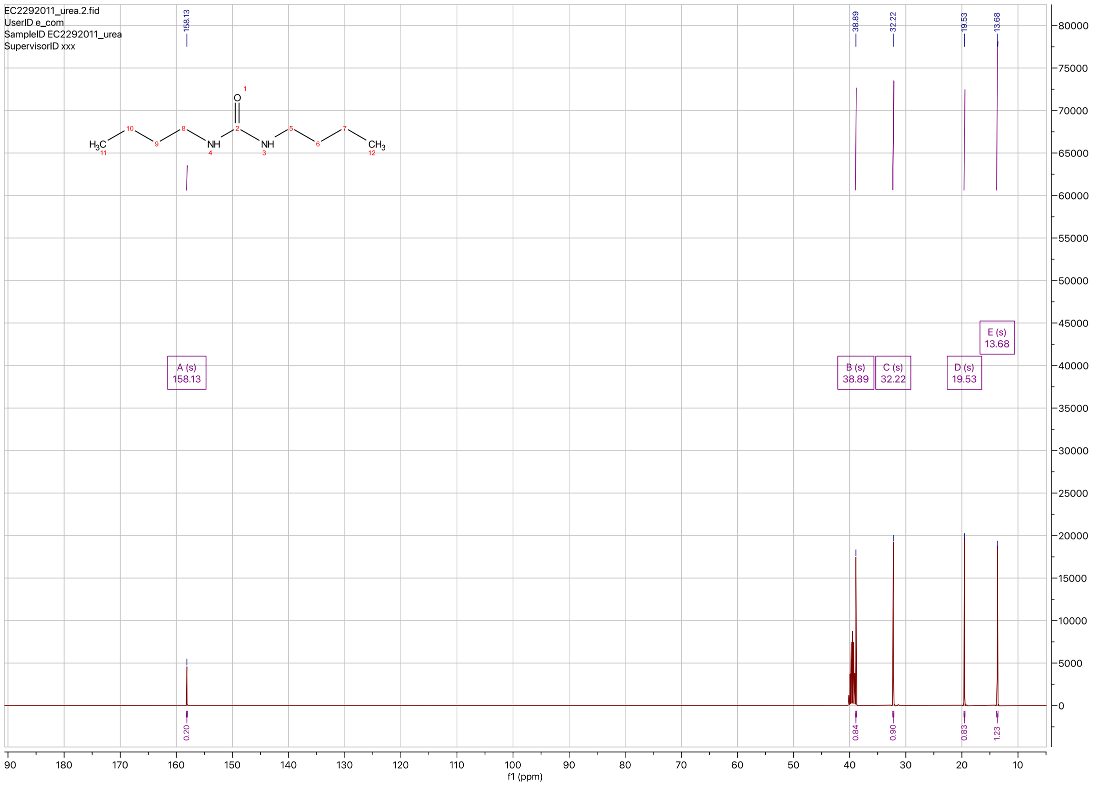

^{1}H NMR (400 MHz, DMSO-d_6) a of **2**

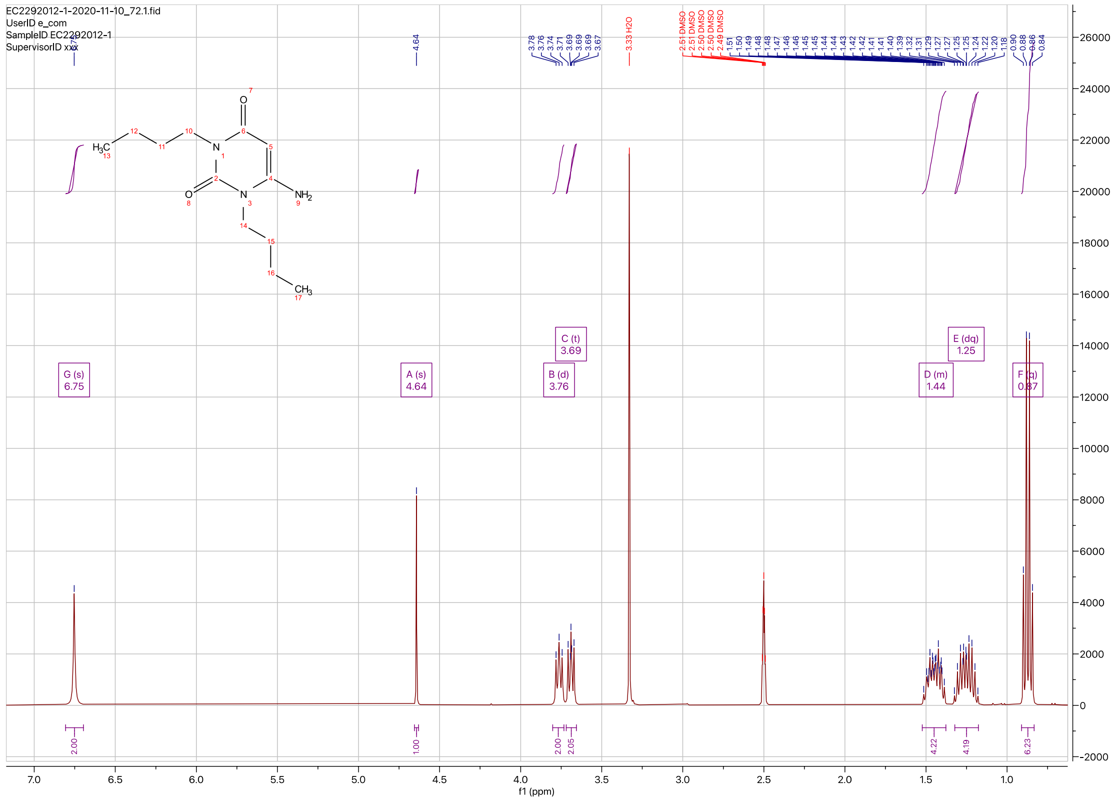

^{13}CNMR (101.62 MHz, DMSO-d_6) of **2**

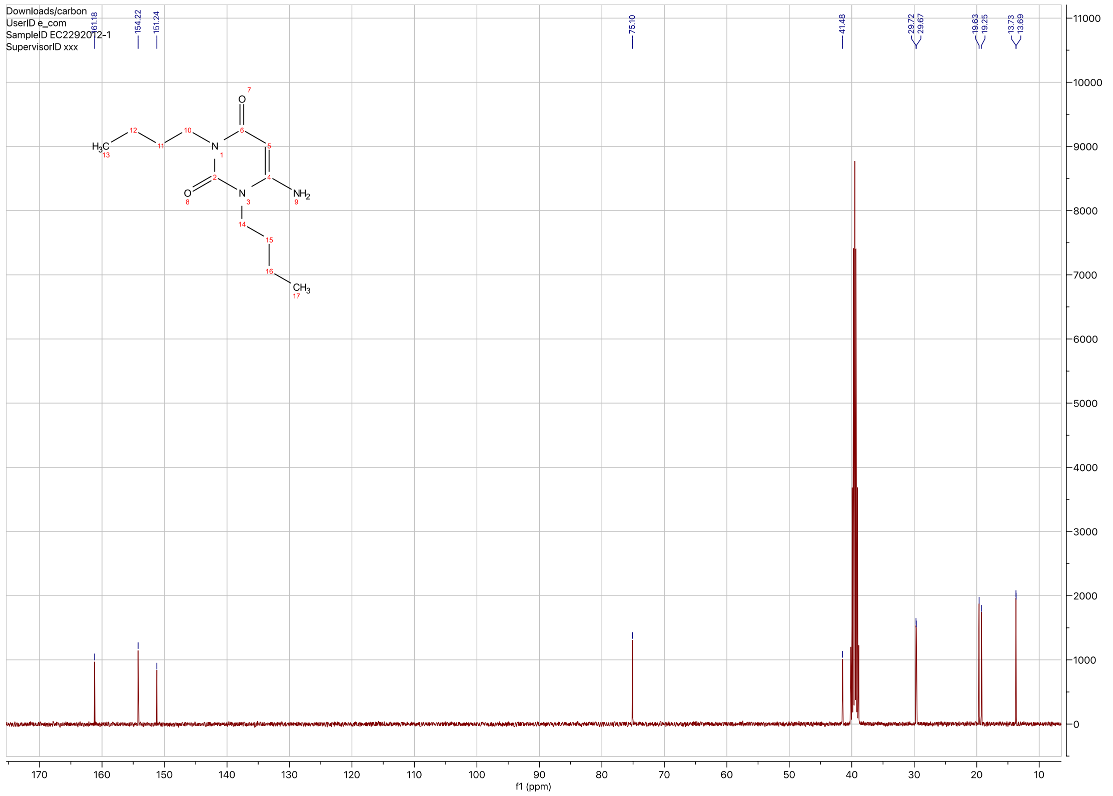

^{1}H NMR (400 MHz, DMSO-d_6) a of **3**

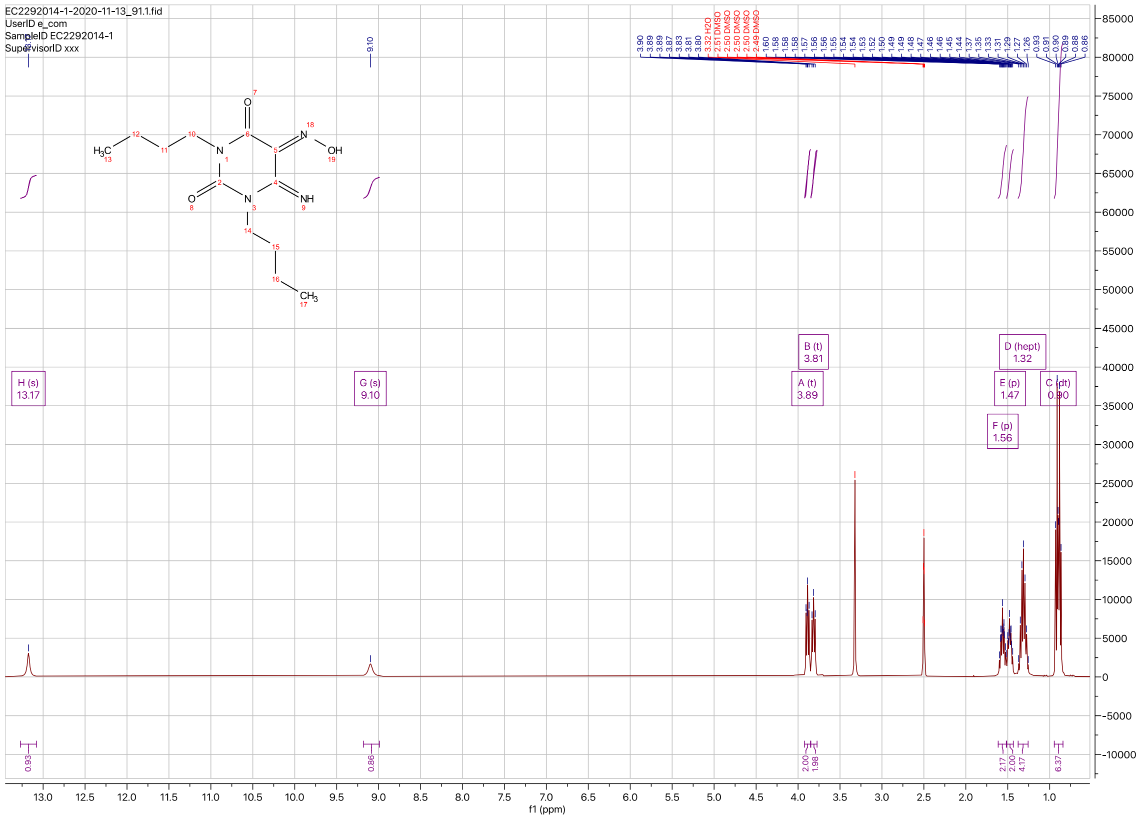

^{13}CNMR (101.62 MHz, DMSO-d_6) of **3**

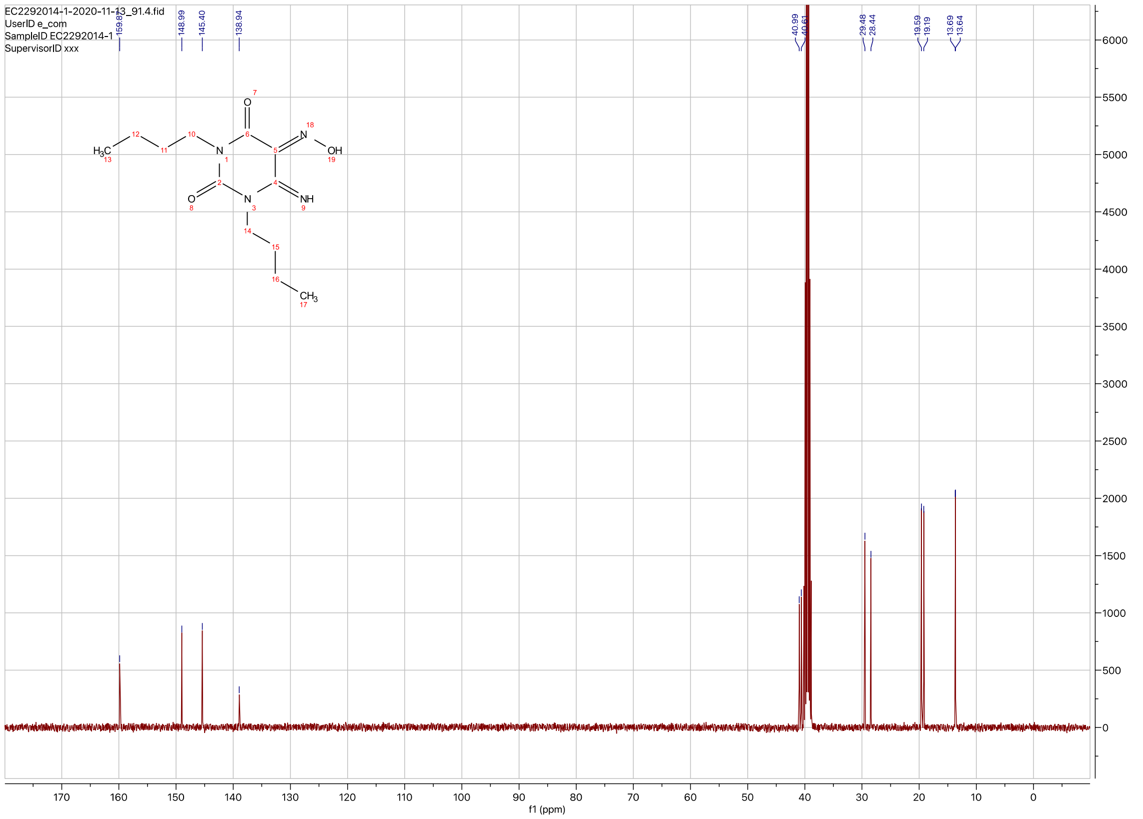

^{1}H NMR (400 MHz, $CDCl_3$) a of **4**

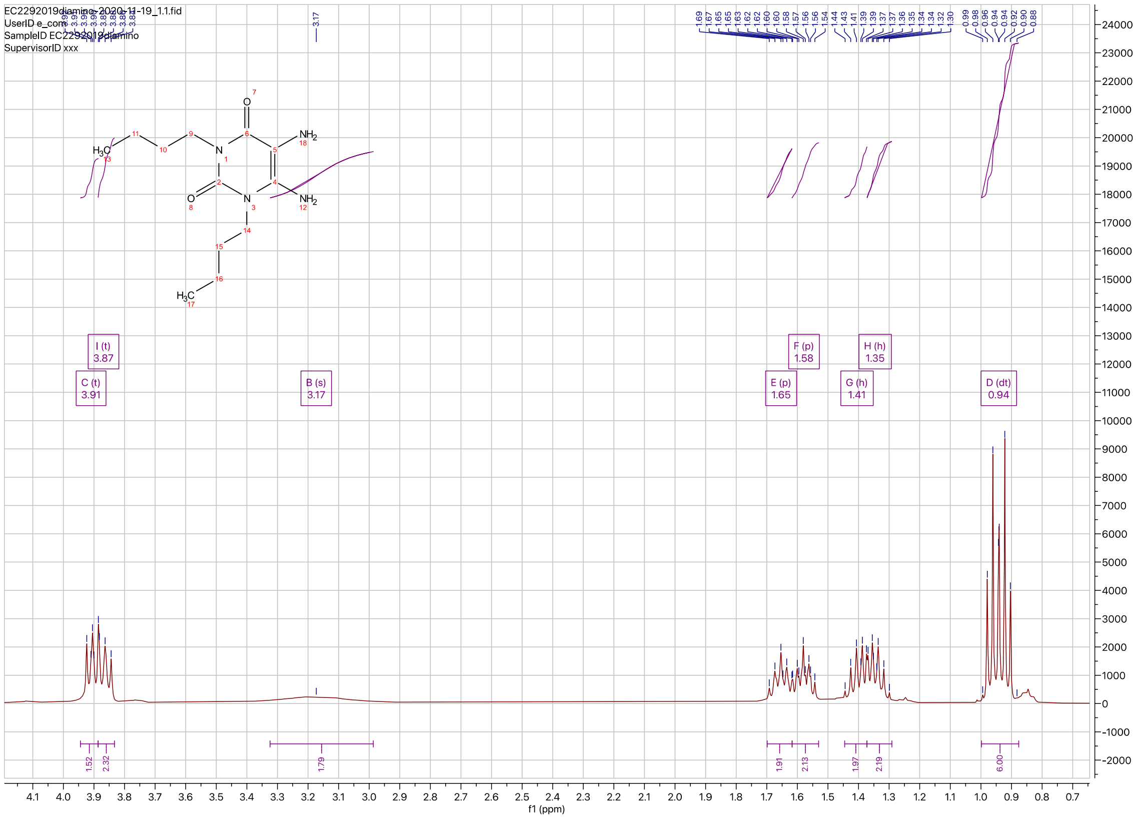

^{13}CNMR (101.62 MHz, $CDCl_3$) a of **4**

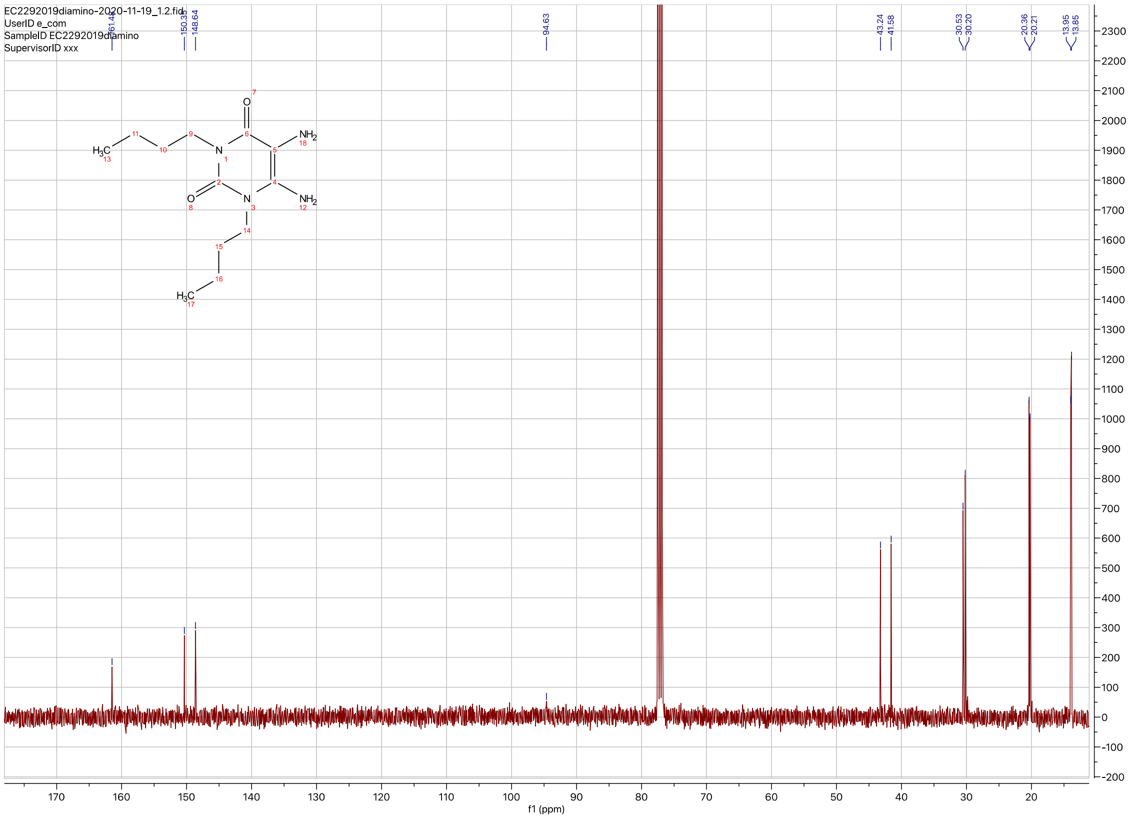

^{1}H NMR (400 MHz, DMSO-d_6) a of **5**

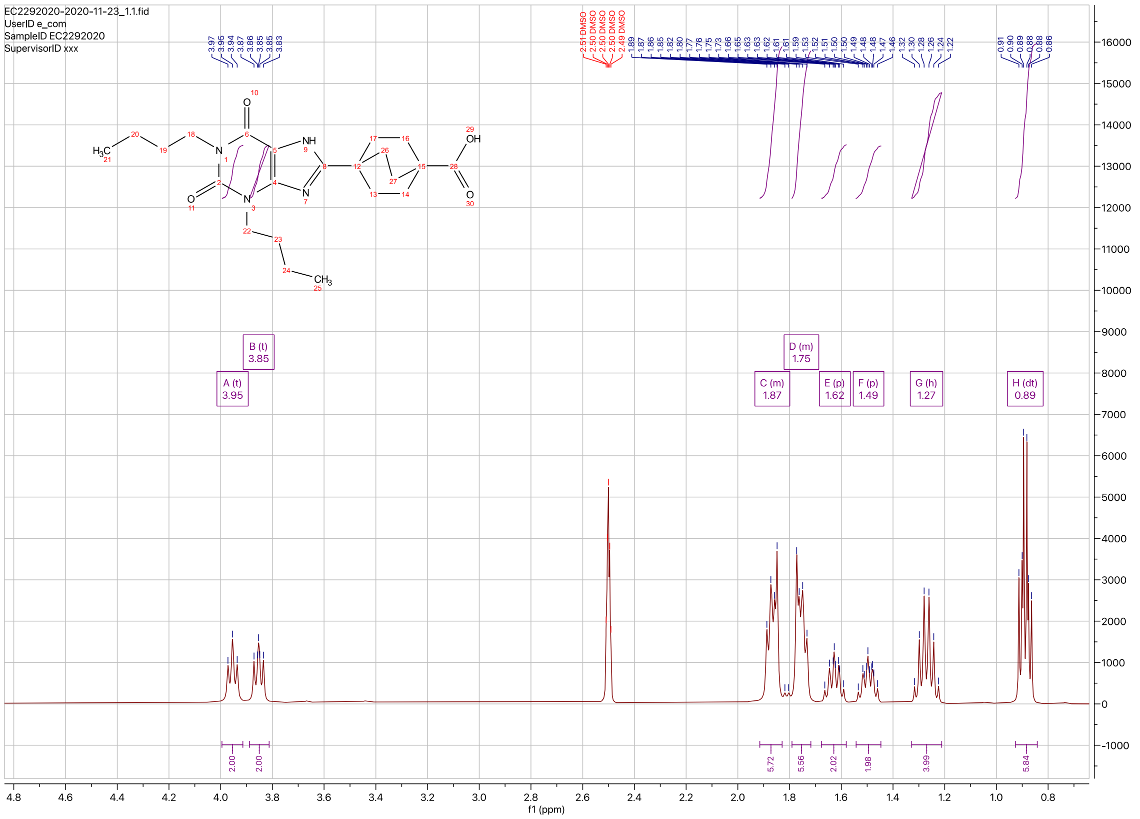

^{13}CNMR (101.62 MHz, DMSO-d_6) of **5**

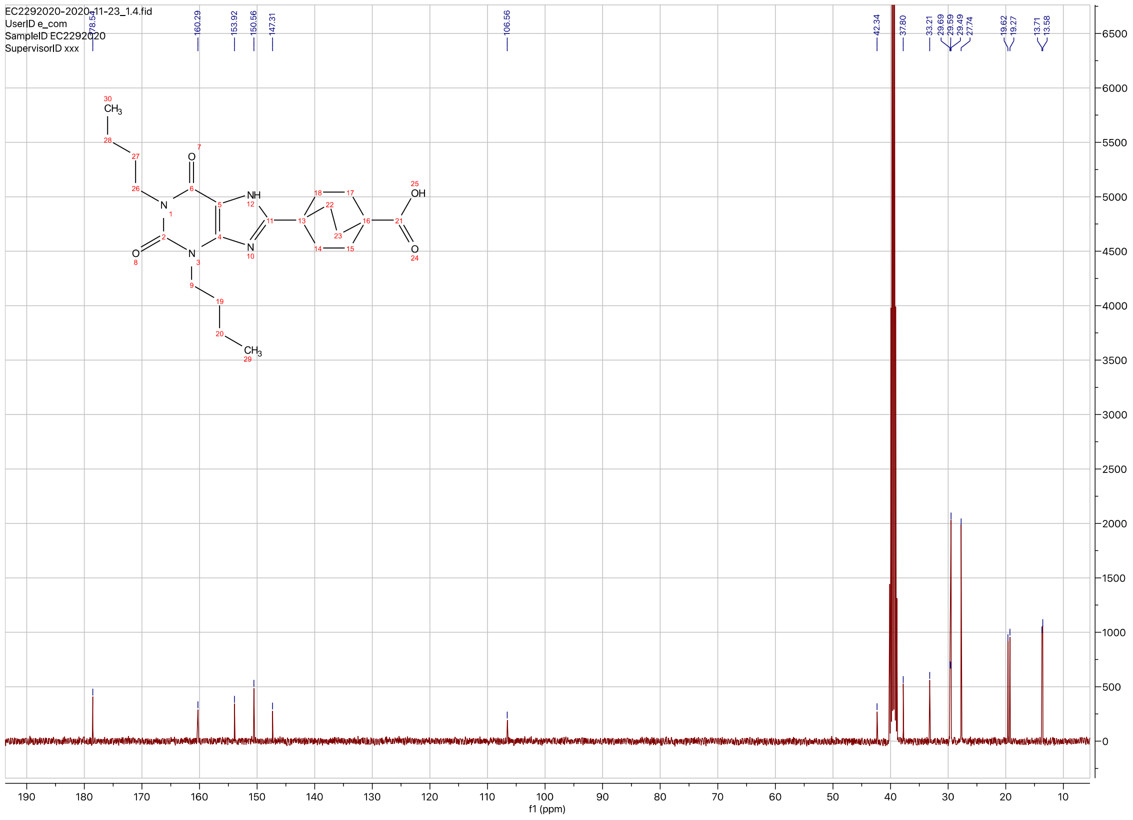

^{1}H NMR (400 MHz, MeOD$_4$) a of **6**

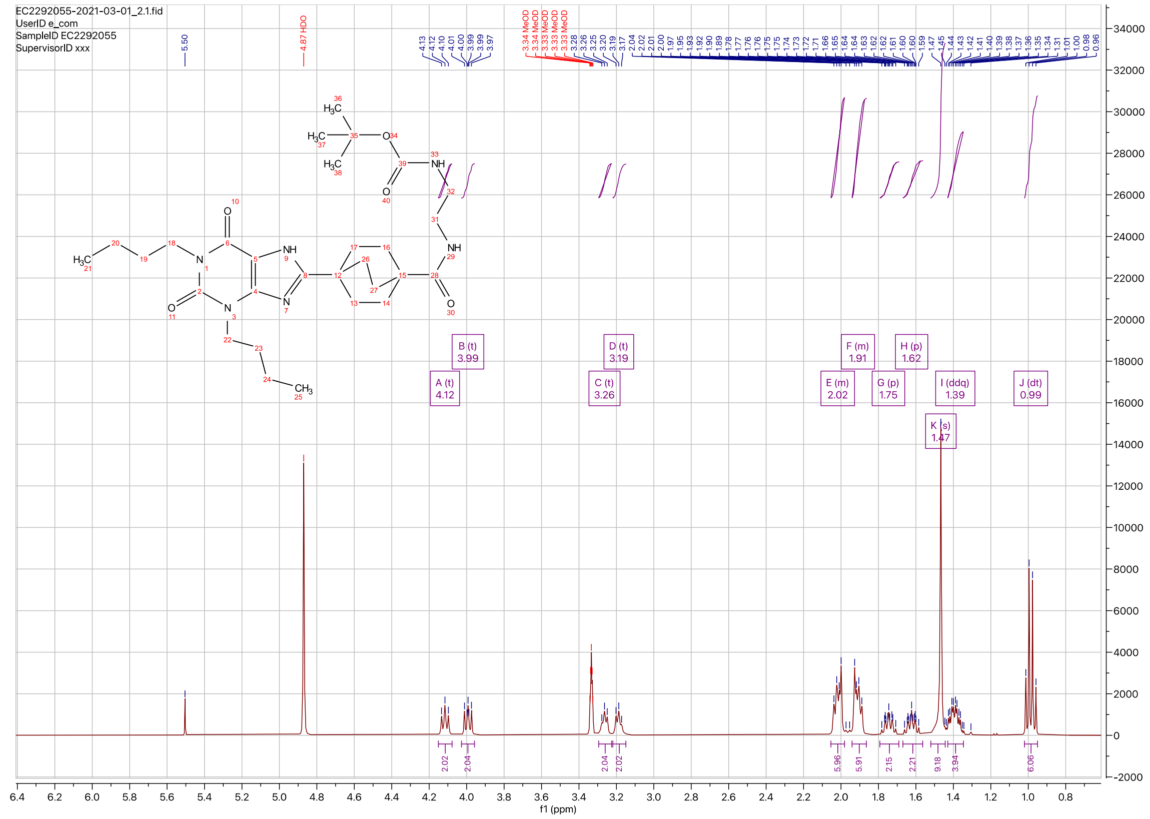

^{13}CNMR (101.62 MHz, MeOD$_4$) of **6**

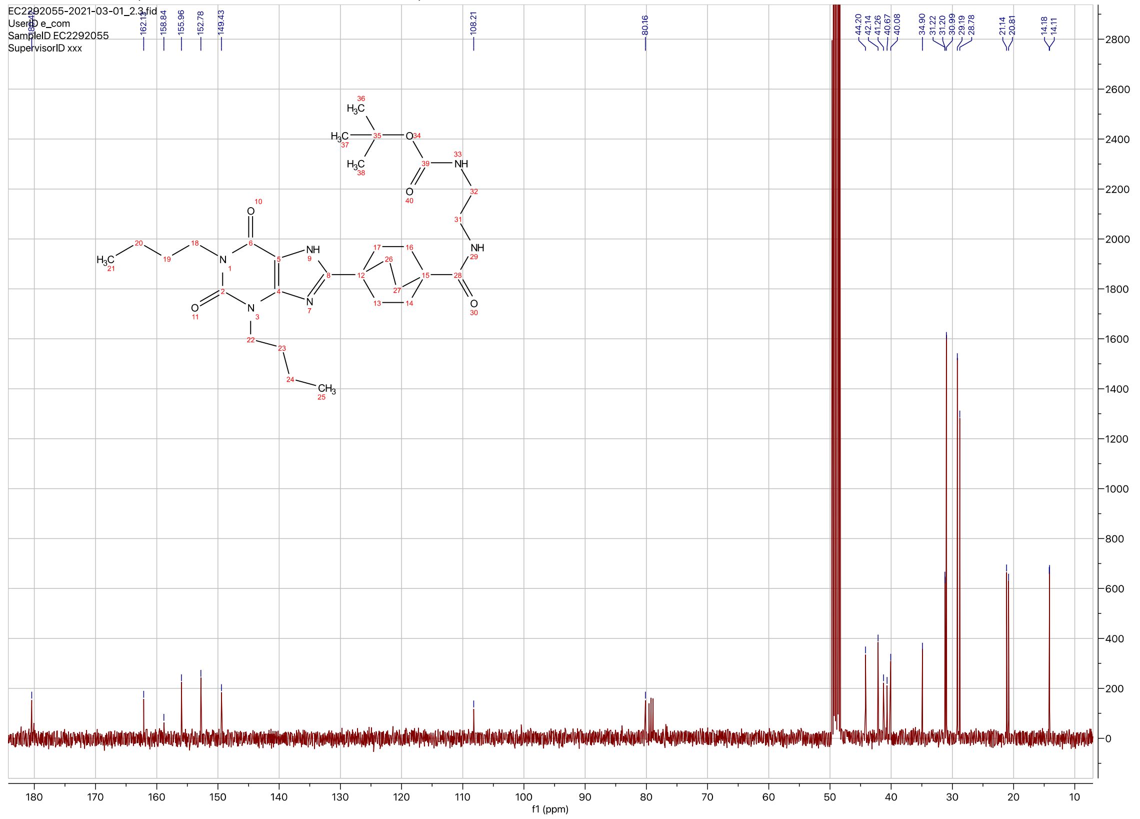

^{1}H NMR (400 MHz, MeOD$_4$) a of **8**

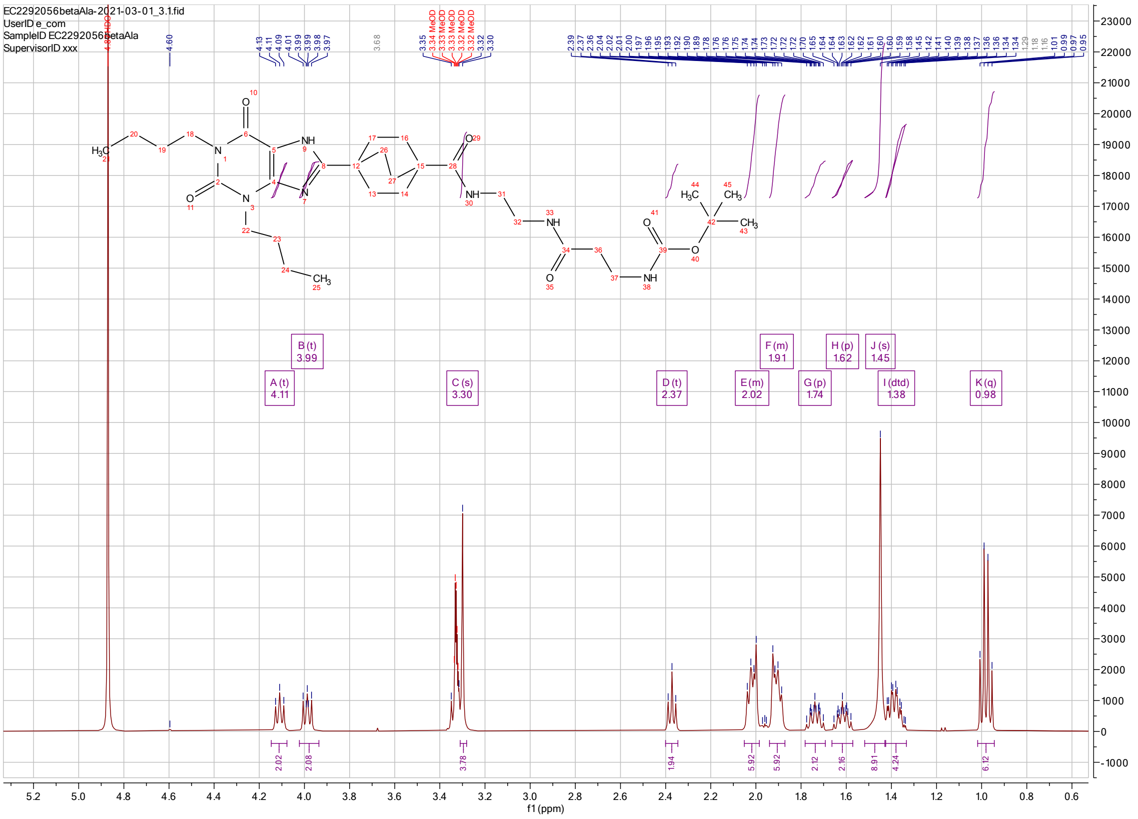

^{13}CNMR (101.62 MHz, MeOD$_4$) of **8**

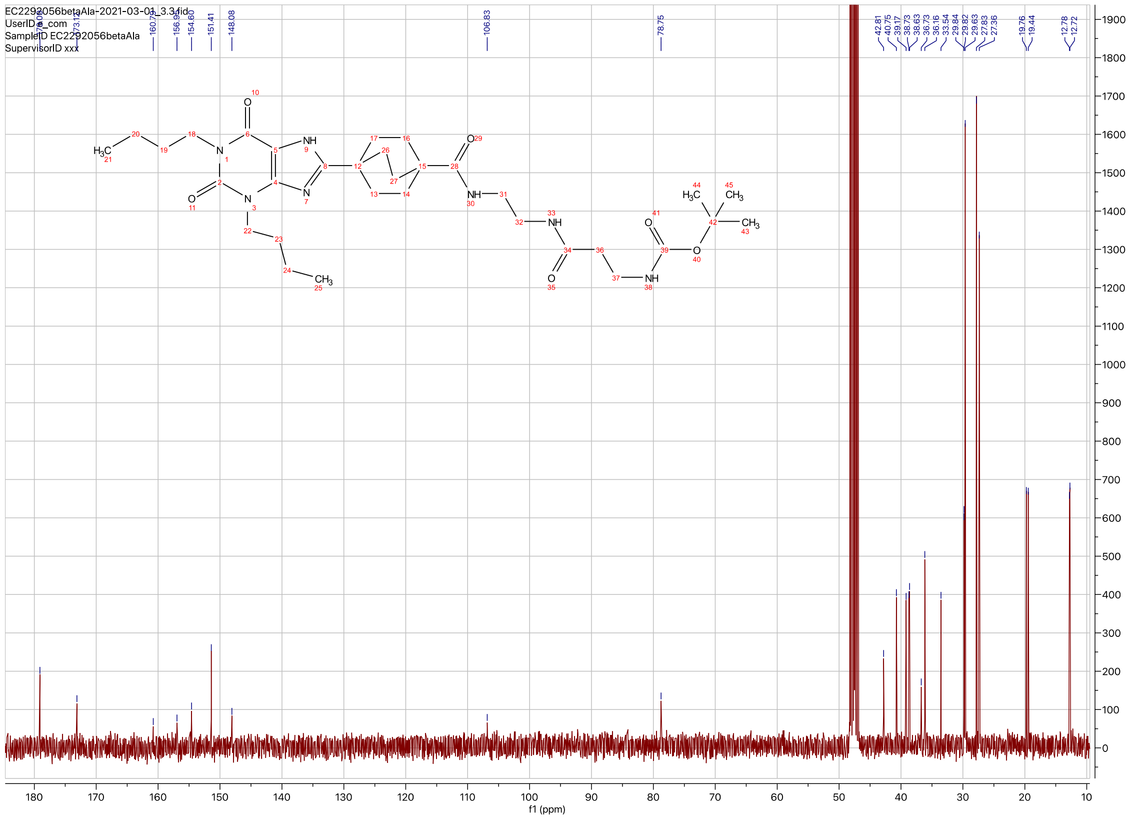

^{1}H NMR (400 MHz, $MeOD_4$) a of **10**

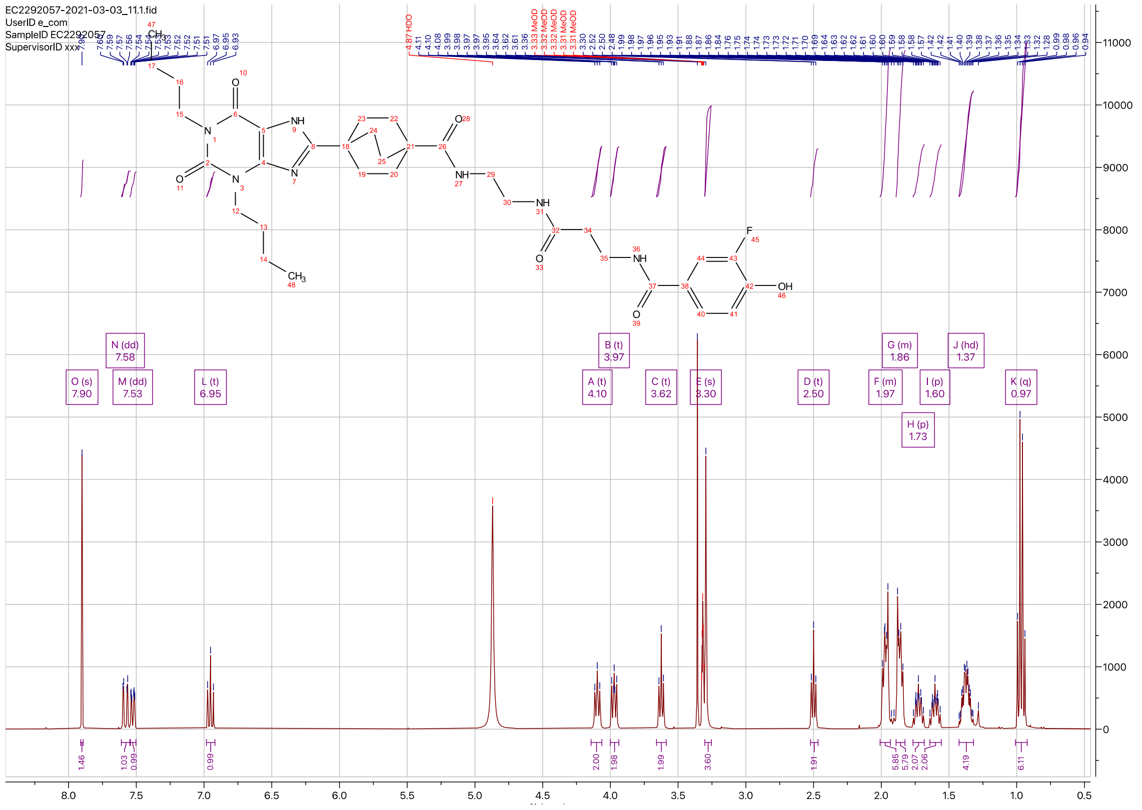

^{13}CNMR (101.62 MHz, $MeOD_4$) of **10**

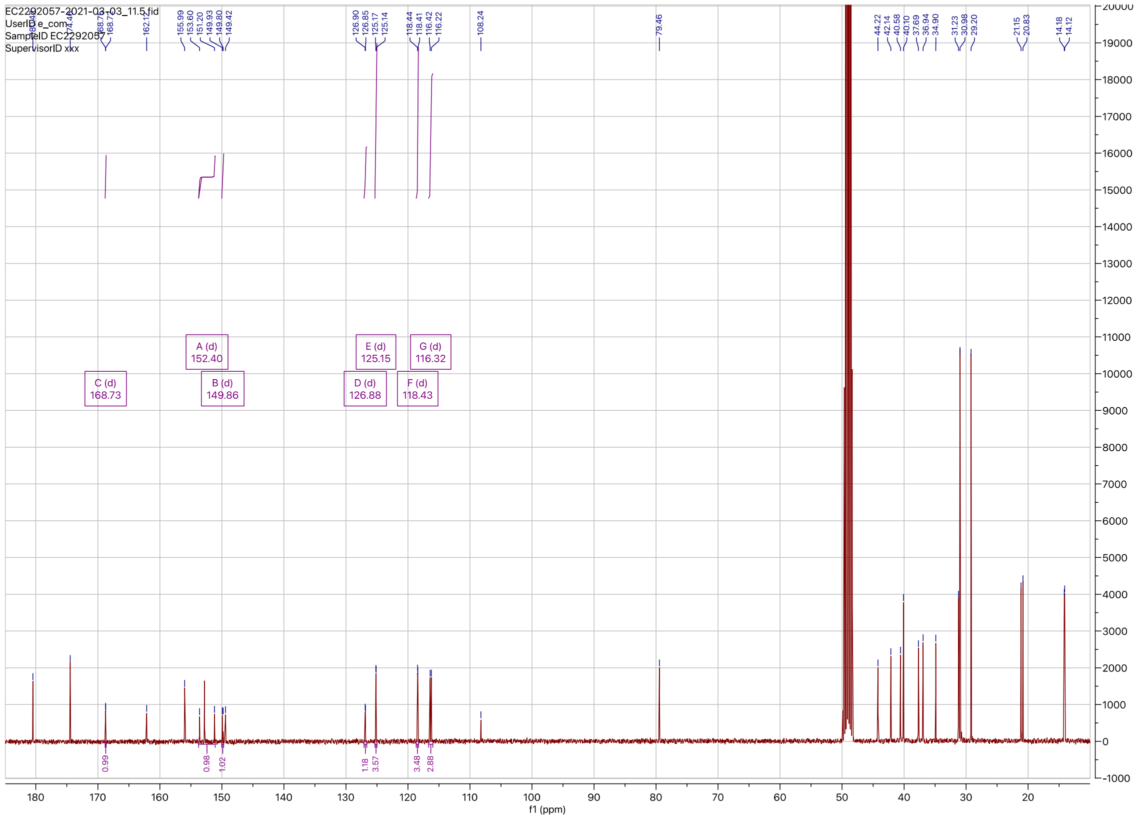

^{19}F NMR (377 MHz, $MeOD_4$) of **10**

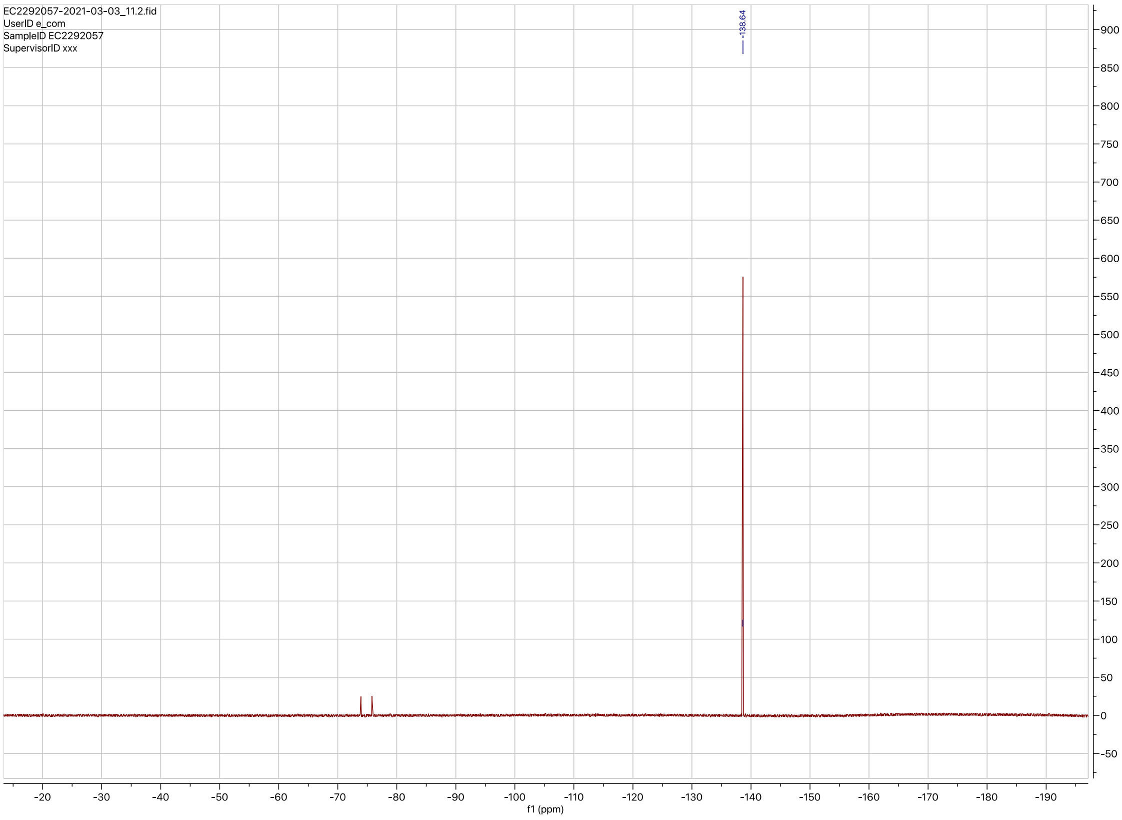

^{1}H NMR (400 MHz, $MeOD_4$) a of **PEG-TCO**

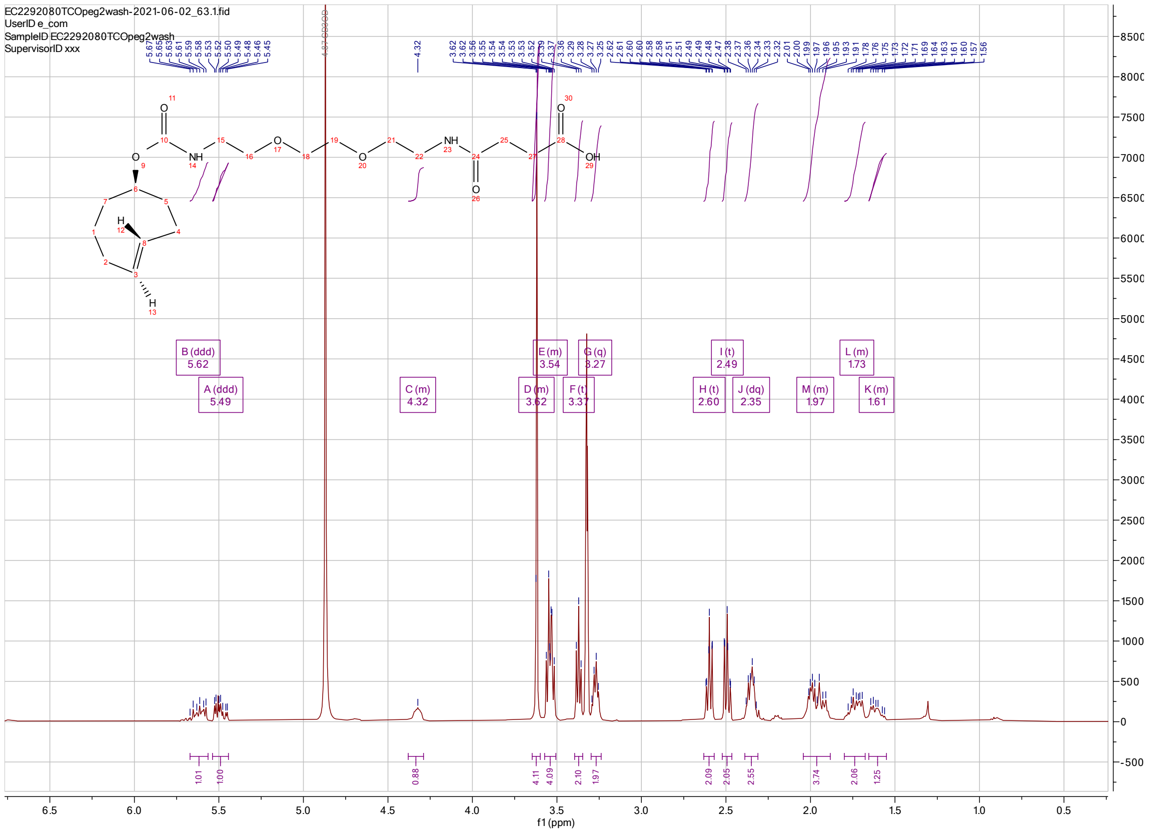

^{1}H NMR (400 MHz, $CDCl_3$) a of **12 (PEG-TCO)**

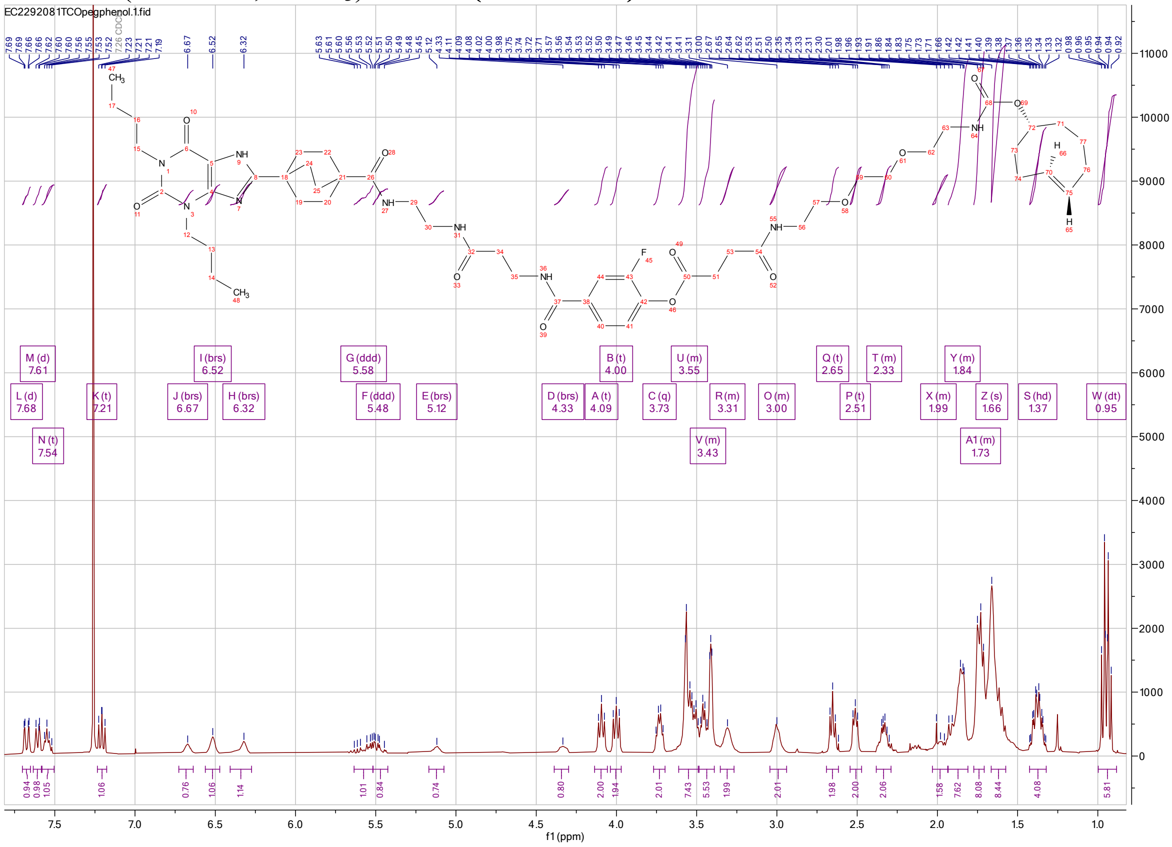

REFERENCES

(1) Liu, D. S.; Tangpeerachaikul, A.; Selvaraj, R.; Taylor, M. T.; Fox, J. M.; Ting, A. Y. Diels–Alder Cycloaddition for Fluorophore Targeting to Specific Proteins inside Living Cells. *J. Am. Chem. Soc.* **2012**, *134* (2), 792–795. https://doi.org/10.1021/ja209325n.

(2) Stoddart, L. A.; Johnstone, E. K. M.; Wheal, A. J.; Goulding, J.; Robers, M. B.; Machleidt, T.; Wood, K. V.; Hill, S. J.; Pfleger, K. D. G. Application of BRET to Monitor Ligand Binding to GPCRs. *Nat. Methods* **2015**, *12* (7), 661–663. https://doi.org/10.1038/nmeth.3398.

(3) Comeo, E.; Kindon, N. D.; Soave, M.; Stoddart, L. A.; Kilpatrick, L. E.; Scammells, P. J.; Hill, S. J.; Kellam, B. Subtype-Selective Fluorescent Ligands as Pharmacological Research Tools for the Human Adenosine A_{2A} Receptor. *J. Med. Chem.* **2020**, *63* (5), 2656–2672. https://doi.org/10.1021/acs.jmedchem.9b01856.

(4) Cooper, S. L.; Soave, M.; Jörg, M.; Scammells, P. J.; Woolard, J.; Hill, S. J. Probe Dependence of Allosteric Enhancers on the Binding Affinity of Adenosine A_1 -receptor Agonists at Rat and Human A_1 -receptors Measured Using N Ano BRET. *Br. J. Pharmacol.* **2019**, *176* (7), 864–878. https://doi.org/10.1111/bph.14575.

(5) Soave, M.; Kellam, B.; Woolard, J.; Briddon, S. J.; Hill, S. J. NanoBiT Complementation to Monitor Agonist-Induced Internalization Adenosine A1 Receptor.

(6) Schmidt, T. G. M.; Batz, L.; Bonet, L.; Carl, U.; Holzapfel, G.; Kiem, K.; Matulewicz, K.; Niermeier, D.; Schuchardt, I.; Stanar, K. Development of the Twin-Strep-Tag® and Its Application for Purification of Recombinant Proteins from Cell Culture Supernatants. *Protein Expr. Purif.* **2013**, *92* (1), 54–61. https://doi.org/10.1016/j.pep.2013.08.021.

(7) Cooper, S. L.; Wragg, E. S.; Pannucci, P.; Soave, M.; Hill, S. J.; Woolard, J. Regionally Selective Cardiovascular Responses to Adenosine A2A and A2B Receptor Activation. *FASEB J.* **2022**, *36* (4), e22214. https://doi.org/10.1096/fj.202101945R.

(8) Lindsay, R. M. Nerve Growth Factors (NGF, BDNF) Enhance Axonal Regeneration but Are Not Required for Survival of Adult Sensory Neurons. *J. Neurosci.* **1988**, *8* (7), 2394–2405. https://doi.org/10.1523/JNEUROSCI.08-07-02394.1988.

(9) Malin, S. A.; Davis, B. M.; Molliver, D. C. Production of Dissociated Sensory Neuron Cultures and Considerations for Their Use in Studying Neuronal Function and Plasticity. *Nat. Protoc.* **2007**, *2* (1), 152–160. https://doi.org/10.1038/nprot.2006.461.

(10) Schindelin, J.; Arganda-Carreras, I.; Frise, E.; Kaynig, V.; Longair, M.; Pietzsch, T.; Preibisch, S.; Rueden, C.; Saalfeld, S.; Schmid, B.; Tinevez, J.-Y.; White, D. J.; Hartenstein, V.; Eliceiri, K.; Tomancak, P.; Cardona, A. Fiji: An Open-Source Platform for Biological-Image Analysis. *Nat. Methods* **2012**, *9* (7), 676–682. https://doi.org/10.1038/nmeth.2019.

(11) Goulding, J.; Kondrashov, A.; Mistry, S. J.; Melarangi, T.; Vo, N. T. N.; Hoang, D. M.; White, C. W.; Denning, C.; Briddon, S. J.; Hill, S. J. The Use of Fluorescence Correlation Spectroscopy to Monitor Cell Surface B2-adrenoceptors at Low Expression Levels in Human Embryonic Stem Cell-derived Cardiomyocytes and Fibroblasts. *FASEB J.* **2021**, *35* (4). https://doi.org/10.1096/fj.202002268R.

(12) Corriden, R.; Kilpatrick, L. E.; Kellam, B.; Briddon, S. J.; Hill, S. J. Kinetic Analysis of Antagonist-occupied adenosine-A_3 Receptors within Membrane Microdomains of Individual Cells Provides Evidence of Receptor Dimerization and Allosterism. *FASEB J.* **2014**, *28* (10), 4211–4222. https://doi.org/10.1096/fj.13-247270.
